# Automated transition state generation for mechanistic exploration in organic synthesis

**DOI:** 10.1038/s41467-026-77230-8

**Published:** 2026-08-27

**Authors:** Li-Cheng Xu, Junyi An, Weiqi Liu, Yun-Fei Shi, Chong-Lei Ji, Fenglei Cao, Yuan Qi

**Affiliations:** 1 Shanghai Academy of Artificial Intelligence for Science, Shanghai, China; 2 Golab Inc, Shanghai, China; 3 Artificial Intelligence Innovation and Incubation Institute, Fudan University, Shanghai, China; 4 School of Physical Science and Technology, ShanghaiTech University, Shanghai, China

**Keywords:** Cheminformatics, Reaction kinetics and dynamics, Chemical physics

## Abstract

Accurate generation of intricate 3D molecular structures is a fundamental challenge in computational chemistry. Transition states (TS), the transient structures that dictate reaction kinetics, exemplify this challenge, as their calculation remains a major bottleneck in elucidating reaction mechanisms. While generative AI has shown promise in automating TS generation, existing methods are largely confined to simple systems, struggling with the complex structures like those in transition metal catalysis. Here we show a unified framework for general-purpose TS generation, comprising the UniTS-Lib library of 4,391 high-quality structures spanning 42 elements and diverse chemical transformations, coupled with the UniTS-Gen diffusion model that generates 3D TS configurations from 2D reactant graphs using a custom-designed higher-degree equivariant network. Validation demonstrates UniTS-Gen’s superior accuracy and robust generalization to unseen chemical systems. We show that UniTS-Gen provides reliable initial guesses and accelerates discovery by locating kinetically favored conformations. This work provides a scalable and transferable solution for automating mechanistic studies in organic synthesis and beyond.

## Abstract (web summary)

The authors develop UniTS, a unified framework for automated transition state generation that combines a diverse transition state dataset with equivariant diffusion learning to accelerate mechanistic exploration.

## Introduction

The transition state (TS), representing the saddle point on the reaction coordinate of highest potential energy, is an intricate and structurally sensitive species that dictates the kinetics of chemical transformations and is fundamental to elucidating reaction mechanisms^1–3^. In fields of precise synthesis, including asymmetric organometallic catalysis^4^, chiral radical chemistry^5^, and bio-orthogonal chemistry^6^, analyzing TS structures is indispensable for the rational design of effective chemical systems that optimize reactivity and selectivity^7,8^. Despite the critical importance of locating these transient structures, prevailing computational strategies remain heavily reliant on rational initial guesses^9^. Generating plausible TS initial structures typically necessitates labor-intensive trial-and-error and extensive expert supervision. This manual, expertise-dependent paradigm scales poorly and is increasingly unable to keep pace with the rapid growth of novel reaction systems.

Inspired by the transformative success of deep learning in predicting biological macromolecules^10–12^, the chemical community has begun to harness generative AI to automate the identification of transition states^13^, aiming to transcend the constraints of expert intuition. The methodology in this field has evolved rapidly, progressing from early heuristics utilizing geometric distance-based methods^14–17^ to sophisticated diffusion-based generative frameworks^18–20^. Representative studies by Kim et al.^19^ and Duan et al.^18,20^ independently established the feasibility of applying diffusion models to generate plausible TS geometries, demonstrating their utility for elementary reactions with limited elemental diversity. Subsequent work by Liu et al.^21^ extended this approach from unimolecular systems to a specific class of bimolecular systems. Collectively, these contributions have significantly advanced the automation of reaction mechanism investigation^13,22,23^.

However, current generative frameworks are confined to simplified systems, with datasets limited in elemental diversity and molecular size. Developing a general-purpose TS generation model for authentic synthetic scenarios, such as transition metal catalysis^24^, remains a formidable challenge. To deploy generative AI effectively in realistic mechanistic studies, two critical challenges must be addressed: first, the construction of a comprehensive TS dataset covering diverse reaction types, elemental compositions, and broad atom-count distributions; and second, the development of a generative architecture with sufficient expressive power to model the immense structural diversity of transition states from relatively sparse data, while maintaining robust generalization to unseen chemical manifolds.

**Fig. 1:**
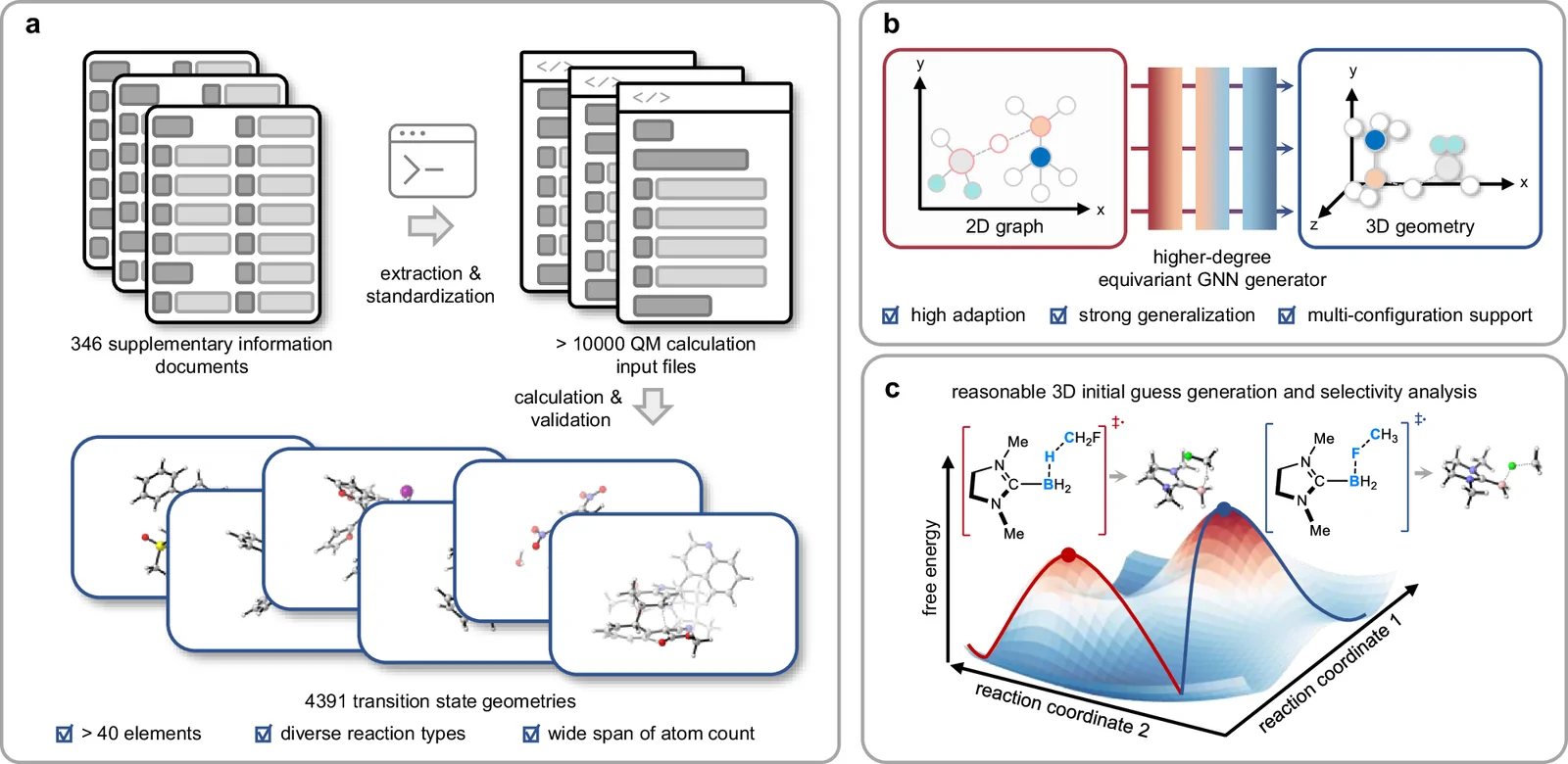
A unified framework for automated transition state discovery. **a** The automated workflow for constructing UniTS-Lib. This pipeline processes 346 Supplementary Information documents to standardize over 10,000 initial quantum mechanical (QM) inputs, which are subjected to rigorous DFT optimization and validation to yield 4391 high-quality transition state geometries. **b** The architecture of the UniTS-Gen model. The model utilizes a higher-degree equivariant GNN as its denoising kernel to generate 3D TS geometries from a 2D molecular graph input, enabling robust generalization and multi-configuration support. **c** Demonstration of UniTS-Gen in generating reasonable 3D initial guesses and its utility in subsequent selectivity analysis. QM quantum mechanical; GNN graph neural network.

Here, we bridge this gap by introducing a unified framework encompassing the automated construction of a complex TS dataset and the development of a high-accuracy, highly generalizable end-to-end generation model for predicting high-quality initial guesses of 3D TS structures from 2D molecular graphs. We first utilized the data construction workflow within this framework, a process that extracts coordinates of TS from literature Supplementary Information documents followed by meticulous density functional theory (DFT) level TS optimization and frequency-based validation, enabling us to establish UniTS-Lib, a library of 4,391 consistently optimized TS structures derived from authentic mechanistic explorations of complex organic synthesis (Fig. 1a). To address the inherent challenge posed by the rich structural diversity and comparative data sparsity of UniTS-Lib, we developed UniTS-Gen, a diffusion model with an architecture that centers on a well-designed higher-degree SE(3)-equivariant graph neural network (GNN)^25–27^ specifically designed as its denoising kernel (Fig. 1b). By carefully design, UniTS-Gen takes only the 2D molecular graph of the reactants as input and generates the corresponding 3D TS geometries exhibiting rich conformational and configurational diversity. We extensively validated UniTS-Gen across three benchmarks—the classic Transition1x^28^, a [3 + 2]-cycloaddition dataset^29^ known for modeling bio-orthogonal reactions and exhibiting wide elemental compositional variance, and our newly constructed UniTS-Lib. A series of comprehensive experiments robustly demonstrates that UniTS-Gen achieves high structural accuracy, superior generalization capability, and the ability to capture distinct TS structures critical for subsequent kinetic barrier estimation (Fig. 1c). This work thus establishes a robust and unified framework that provides a powerful solution to the multifaceted challenges inherent in automating realistic chemical mechanistic discovery.

## Results

### UniTS-Lib: a versatile and high-quality TS library

To establish a robust data foundation for training the general-purpose TS generation model, we developed an automated workflow (Fig. 1a) that efficiently extracts atomic types and coordinates from the Supplementary Information of organic synthesis literature and standardizes them into quantum mechanical (QM) input files. By processing 346 Supplementary Information documents, we successfully extracted and standardized over 10,000 initial QM calculation inputs. Notably, during the file generation process, we explicitly considered the radical, anionic, and cationic states for species that are not stable neutrals, thereby ensuring that our data broadly covers charged and radical TS structures commonly encountered in mechanistic studies. We then subjected these initial structures to rigorous DFT-level TS optimization at a consistent accuracy level, followed by frequency analysis combined with graph-matching verification to validate the saddle point identity as a reactive transition state (i.e., a first-order saddle point with bond‑forming/‑breaking events rather than simple conformational changes), yielding a final set of 4391 consistently optimized TS structures. We deliberately adhered to a standardized protocol rather than employing exhaustive, ad-hoc recovery strategies for non-converging structures. The substantial attrition rate observed from the initial pool to the final dataset highlights the intrinsic difficulty of transition state optimization, a task notoriously sensitive to both the precise initial geometry and the chosen computational methodology. Even when starting from reasonable, literature-derived coordinates, variations in basis sets or functionals can frequently impede convergence. This rigorous filtration process underscores the inherent scarcity of high-quality TS data for complex systems and illustrates the formidable challenge of locating correct saddle points.

**Fig. 2:**
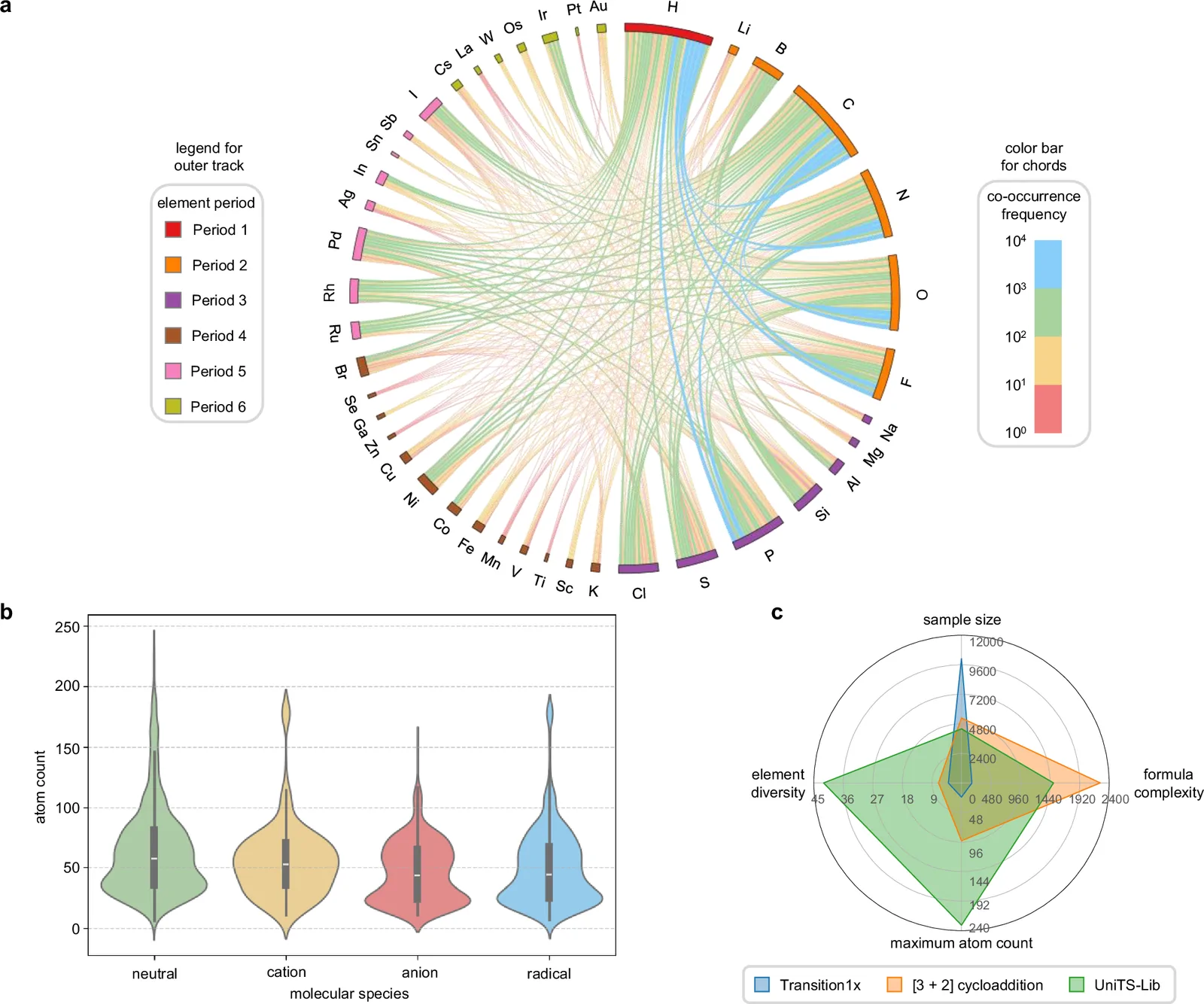
Analysis of the structural and elemental complexity of UniTS-Lib and its comparison to existing TS datasets. **a** The element co-occurrence chord diagram illustrates the elemental richness and relationships within UniTS-Lib, which features 42 elements, including main-group and transition metals essential for catalysis. The outer track length corresponds to individual element frequency, while the chord thickness and color (cooler tones indicate higher frequency) denote the volume of pairwise co-occurrence. **b** Violin plots characterize the atom count distribution across four major molecular species (neutral, cation, anion, and radical transition states). All species exhibit broad size distributions with median atom counts around 50. The violin width represents the kernel density estimate of the distribution. The embedded box indicates the interquartile range (25th–75th percentiles), and the central marker indicates the median. The distributions were calculated from all transition states in UniTS-Lib: neutral (*n* = 2264), cation (*n* = 294), anion (*n* = 596), and radical (*n* = 1237). **c** A multi-dimensional radar chart quantitatively compares UniTS-Lib (green) against the classic Transition1x (blue) and the representative [3 + 2] cycloaddition (orange) datasets across sample size, element diversity, maximum atom count, and formula complexity. Source data are provided as a Source Data file.

Based on our unbiased data curation strategy, UniTS-Lib encompasses over 10 distinct transformation types central to mechanistic investigations, including oxidative addition, transmetallation and cycloaddition (see Supplementary Fig. 2 for details). A quantitative analysis by reaction type is not provided, as many TS structures cannot be uniquely assigned to a single reaction type due to multi‑step or multi‑pathway processes. The dataset features a broad elemental distribution involving 42 elements, with the maximum atomic number being 79 (gold). As elucidated by the element co-occurrence chord diagram in Fig. 2a, the arc length of the outer track corresponds to the frequency of each element, while the thickness and color intensity (with cooler tones indicating higher frequency) of the internal chords depict the volume of pairwise co-occurrence. This visualization reveals that, beyond main-group elements, transition metals pivotal to catalysis, specifically the iron and platinum groups, are substantially represented.

Beyond elemental diversity, UniTS-Lib exhibits remarkable structural complexity. As shown in Fig. 2b, violin plots characterize the atom count distribution across four primary molecular species: neutral, cationic, anionic, and radical transition states. All species display broad distributions with medians centered around 50 atoms. Notably, neutral molecules exhibit the widest span, reaching a maximum of 231 atoms; in total, the entire dataset contains 131 structures exceeding 150 atoms. This extensive coverage across diverse electronic and spin states underscores the dataset’s capacity to represent complex chemical spaces.

Additionally, we multi-dimensionally benchmarked UniTS-Lib against the Transition1x^28^ and [3 + 2] cycloaddition^29^ datasets (Fig. 2c). UniTS-Lib leads in elemental diversity and molecular size breadth while maintaining high formula complexity, encompassing a broader range of reaction types. This makes it the most elementally and structurally complex benchmark, and its relatively smaller sample size underscores the challenge of training general-purpose models on such high-quality data.

### UniTS-Gen: a higher-degree equivariant generation model

With the UniTS-Lib dataset in hand, we next sought to develop a generative model capable of learning from such structural and elemental diversity. Recent diffusion-based TS generation models have achieved notable accuracy by using 3D reactant and product geometries as inputs^18,20^. While effective for well-defined benchmark systems, this strategy is less practical for complex, mechanistically underexplored reactions, where obtaining reliable 3D product structures is non-trivial and may inadvertently restrict the conformational and configurational exploration of transition states. We therefore designed UniTS-Gen to generate 3D TS geometries directly from 2D reactant graphs and reaction site indices. This input scheme preserves flexibility in configurational sampling and lowers the barrier to application in novel reaction systems.

**Fig. 3:**
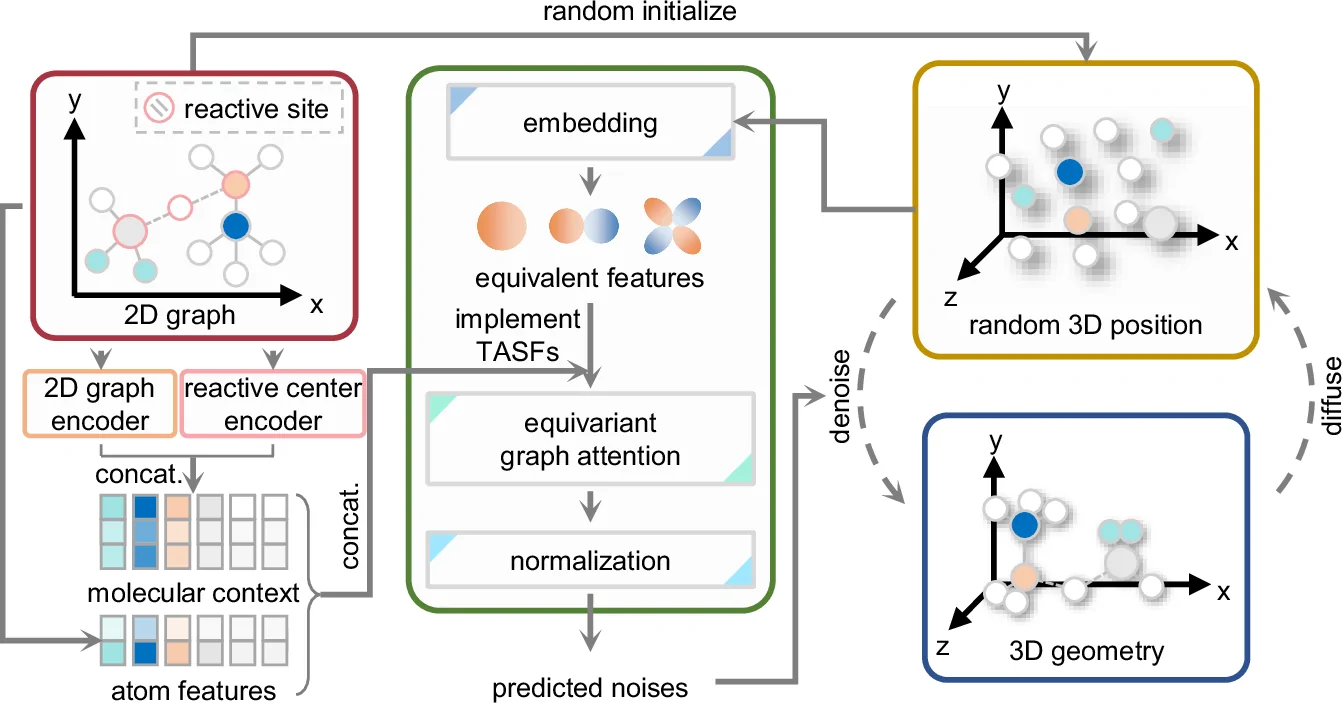
Architecture of the UniTS-Gen conditional diffusion model for 3D TS generation. The generative framework is based on the higher-degree equivariant GNN as the denoising kernel. The model is engineered as a conditional diffusion process where the inputs are restricted to the 2D molecular graph of the reactants and atomic indices defining the reactive site (red box). These inputs are encoded into a molecular context that guides the reverse diffusion process. The HiEGNN denoising module (green box) iteratively refines the initial random 3D coordinates (yellow box) based on both the noisy structure and the molecular context. The HiEGNN utilizes higher-degree SE(3)-equivariance to accurately model complex 3D structures while inherently respecting rotational and translational equivariances, ultimately predicting the required noise to generate the precise 3D TS geometry (blue box). concat. concatenated; TASFs topology-augmented scalar features.

To achieve sufficient structural accuracy and generalization on such complex, diverse, yet relatively sparse TS data, the core architecture must be capable of deeply capturing and learning molecular 3D structural information. Given that higher-degree equivariant GNNs leverage spherical harmonics to precisely capture subtle changes in molecular 3D structure^25–27^, we reasoned that this enhanced representational capacity is particularly crucial for modeling the intricate and often highly distorted geometries of transition states. We therefore posited that this model type serves as an ideal denoising kernel for generating complex 3D TS structures within a diffusion framework. Accordingly, we designed a higher-degree equivariant denoising kernel, termed HiEGNN, and built the UniTS-Gen framework upon this kernel, as illustrated in Fig. 3.

UniTS-Gen is engineered as a conditional diffusion model built around the HiEGNN kernel. Its input consists solely of the 2D molecular graph of the reactants (or intermediates), augmented with atomic indices that define the reactive site. These indices serve as an essential conditioning feature for distinguishing among multiple possible active sites. These graph-based inputs are encoded by a 2D molecular encoders^30^ to generate latent representations that capture topological and compositional information. Crucially, to enable these 2D-derived features to guide 3D structure generation within the equivariant framework, we introduce a mechanism termed topology-augmented scalar features (TASFs). TASFs inject the encoded 2D graph information into the *L*_*0*_ spherical components of HiEGNN’s equivariant representations, effectively conditioning the 3D geometric refinement process on the molecular connectivity and reactive site context. Starting from random coordinates initialized based on the input atom count, the model iteratively denoises the structure toward a chemically meaningful 3D TS geometry, with TASFs ensuring that each denoising step remains anchored to the topological constraints defined by the input graph.

The core of UniTS-Gen is the HiEGNN denoising module (Fig. 3, green box), which leverages higher-degree SE(3)-equivariance, a vital property that ensures the model inherently respects the rotational and translational equivariances of 3D space, which is important for the accurate and stable prediction of complex coordinates. During the reverse diffusion process, HiEGNN refines the 3D coordinates by integrating the instantaneous noisy structure with the molecular context, resulting in the generation of precise TS geometries that exhibit the rich conformational and configurational diversity required for comprehensive mechanistic analysis (see “Methods” and Supplementary Fig. 5 and Fig. 6 for details of the diffusion and sampling process).

### Structural accuracy and generalization in TS generation

**Fig. 4:**
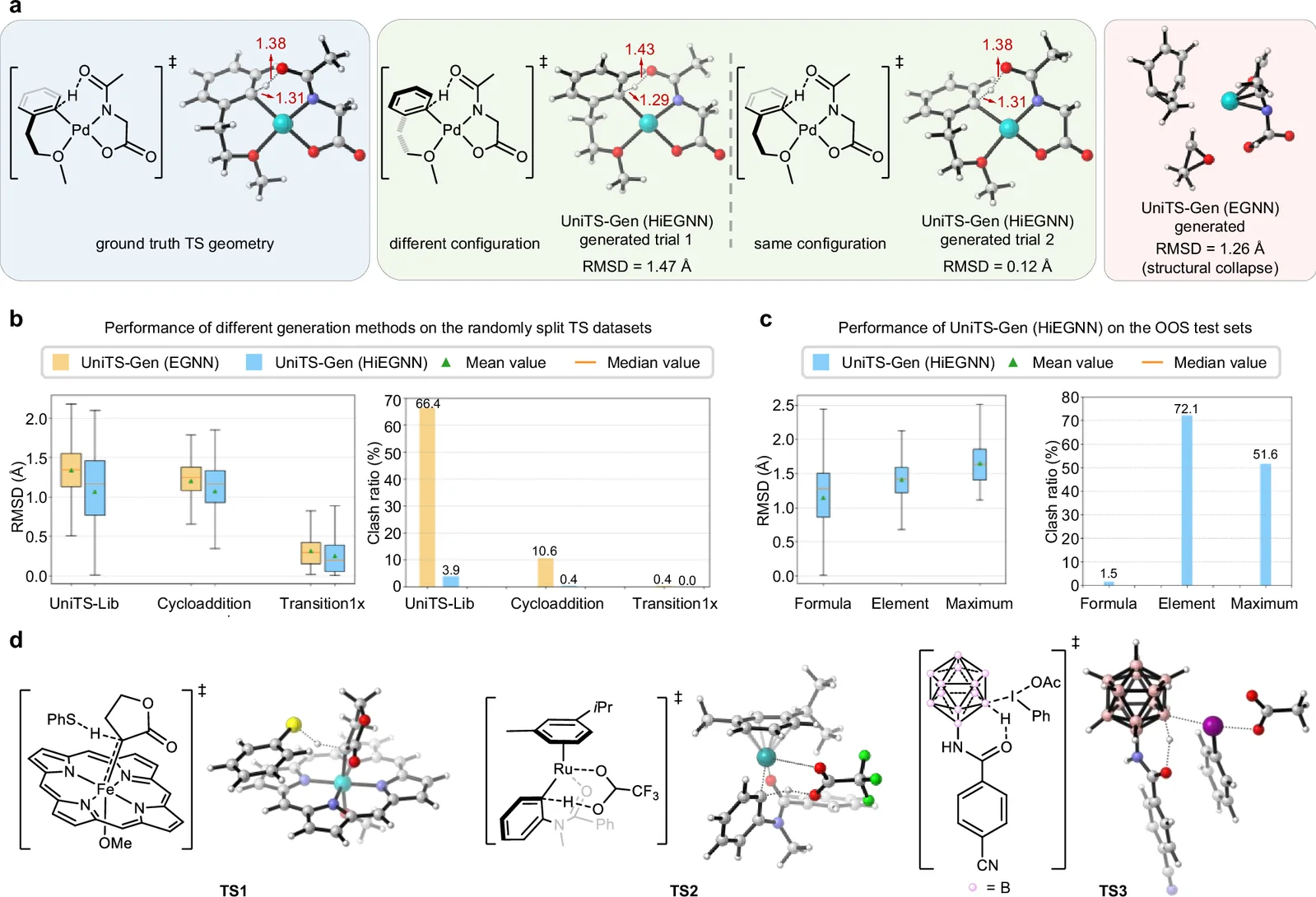
Superior structural accuracy and robust generalization of UniTS-Gen using the higher-degree equivariant denoising kernel. **a** A representative TS illustrating the generation of multiple chemically valid structures from a single 2D input graph. Two representative structures generated by the HiEGNN kernel are shown in the green panel, one of which achieves high structural accuracy (RMSD = 0.12 Å). In contrast, the standard EGNN kernel generates a structurally collapsed geometry, shown in the red panel. **b** Benchmark comparison of UniTS-Gen (HiEGNN) and UniTS-Gen (EGNN) on the UniTS-Lib, [3 + 2]-cycloaddition, and Transition1x test sets using RMSD (Å) and clash ratio (%). Statistics were calculated on UniTS-Lib (*n* = 440), [3 + 2]-cycloaddition (*n* = 528), and Transition1x (*n* = 1073). **c** Performance of UniTS-Gen (HiEGNN) on three OOS test sets derived from UniTS-Lib (Formula-OOS, Element-OOS, and Maximum-OOS), demonstrating robust generalization to increasingly challenging molecular systems. Statistics were calculated on Formula-OOS (*n* = 403), Element-OOS (*n* = 351), and Maximum-OOS (*n* = 219). For the box plots in **b** and **c**, boxes indicate the interquartile range (25th–75th percentiles), the center line indicates the median, whiskers extend to 1.5 × IQR, and the marker indicates the mean. **d** Three representative structures were generated for challenging OOS reactions, all successfully optimized and verified as true transition states. Source data are provided as a Source Data file. RMSD, root mean square deviation; OOS, out-of-sample.

We systematically evaluated the performance of UniTS-Gen in generating 3D TS structures. We first randomly split the UniTS-Lib dataset into training, validation, and test sets (8:1:1) to assess generation accuracy. Figure 4a visually presents a representative case of a Pd-catalyzed C–H activation TS^31^ from the test set. The blue panel displays the ground truth structure, while the green panels show two independent structures generated by UniTS-Gen (using HiEGNN as the denoising kernel) from the same input molecular graph and reactive site indices. Crucially, the denoising process in UniTS-Gen transforms random noise into ordered structures guided by molecular graph constraints; while the input graph dictates chemical connectivity, it does not rigidly constrain conformational freedom. Consequently, UniTS-Gen can generate diverse valid conformers and configurations from a single 2D graph. As shown, trial 1 yielded a chemically plausible TS structure but with the phenyl ring oriented differently from the ground truth, resulting in a relatively high root mean square deviation (RMSD) of 1.47 Å. In contrast, trial 2 converged to a configuration nearly identical to the ground truth, achieving a significantly lower RMSD of 0.12 Å. Notably, in both trials, the critical interatomic distances within the reactive center—specifically the C–H and O–H distances—closely matched the ground truth, a factor pivotal for successful downstream DFT optimization toward the correct saddle point.

To validate the necessity of the higher-degree equivariant architecture for the complex UniTS-Lib dataset, we benchmarked our HiEGNN kernel against a standard E(n) Equivariant Graph Neural Network (EGNN)^32^ commonly used in 3D molecule generation^33^ (Fig. 4a, red panel). Notably, alternative models like React-OT^20^ was excluded as they require 3D structures of reactant and product, which are often unavailable for novel reactions, whereas our method uses only the 2D reactant graph. The structure generated by the EGNN-based model exhibited severe structural collapse, failing to form a chemically valid geometry despite forming a recognizable phenyl substructure. Paradoxically, due to the alignment-based nature of RMSD calculations, this physically invalid, collapsed structure yielded a lower RMSD (1.26 Å) than the chemically valid but configurationally distinct structure from HiEGNN trial 1 (1.47 Å). This observation underscores that RMSD alone is an insufficient metric for evaluating generative models on complex, multimodal TS landscapes, particularly where configurational diversity is high. To address this, we introduced the “clash ratio” metric, which quantifies the percentage of generated structures containing physically implausible interatomic distances (the detailed calculation method of the clash ratio can be found in the “Methods” section), providing a robust measure of chemical validity.

To comprehensively assess performance across varying levels of complexity, we evaluated both UniTS-Gen (HiEGNN) and UniTS-Gen (EGNN) on three datasets: the complex UniTS-Lib, the classic Transition1x benchmark, and the [3 + 2] dipolar cycloaddition dataset known for its diverse substrate combinations. For Transition1x, we adopted the data split reported by Duan et al.^20^, while for the cycloaddition dataset, we used an 8:1:1 split; single-sample inference was performed for all test structures. As shown in Fig. 4b, the performance gap is stark on the complex UniTS-Lib: while the mean of RMSD improvement of HiEGNN over EGNN appears moderate (1.07 Å vs. 1.34 Å), the difference in chemical validity is profound, with HiEGNN achieving a clash ratio of just 3.9% compared to 66.4% for EGNN. This result conclusively demonstrates the superiority of our higher-degree equivariant design for handling complex transition states. For the simpler Transition1x (molecules <25 atoms, <5 element types) and cycloaddition datasets, the simpler EGNN suffices, though HiEGNN still maintains a precision advantage. Due to the large differences in molecular size, absolute RMSD values are not comparable across the three datasets. In addition, to account for the inherent multi-configuration generation capability of UniTS-Gen, we also performed 10-sample evaluations for these datasets, identifying the best-matching conformer; details are provided in Supplementary Tables 3–6. Furthermore, we compared UniTS-Gen (HiEGNN) with other representative models^18–20^ on the Transition1x test set in the Supplementary Information, including both accuracy and inference time (Supplementary Table 4).

While random splits assess in-distribution performance, the critical test for a general-purpose model is its generalization to unseen chemical spaces. We therefore evaluated the generalizability of UniTS-Gen through three rigorous out-of-sample (OOS) tests on UniTS-Lib. These tests comprised a Formula-OOS set of 403 reactions with unseen elemental compositions, an Element-OOS set of 351 structures containing entirely unseen elements, and a Maximum-OOS set of 219 structures whose atom counts exceeded the maximum size encountered during training. The results (Fig. 4c) show that the model’s performance on the Formula-OOS test (mean of RMSD 1.15 Å, clash ratio 1.5%) closely rivals that of the random split, a finding that underscores its strong extrapolation capability. This unexpected robustness demonstrates UniTS-Gen’s ability to generalize effectively to novel organic reactions involving known elements—the most common scenario in mechanistic research^1^. While performance on the highly challenging Element-OOS and Maximum-OOS tasks indicates room for improvement, these limitations will be addressed by the continuous expansion of UniTS-Lib via our automated data workflow. To better illustrate how different RMSD values correspond to structural variations, we provide in Supplementary Figs. 7–9 representative examples of low, medium, and high RMSD structures generated on the Transition1x test set, the [3 + 2] cycloaddition test set, and the UniTS-Lib Formula-OOS test set, each overlaid with the corresponding reference geometry. Furthermore, we also provide a quantitative analysis of RMSD versus energy barrier errors for these representative structures in Supplementary Figs.10–12. For the Transition1x dataset, structural deviations lead to relatively small energy errors, whereas the more complex [3 + 2] cycloaddition and UniTS‑Lib datasets exhibit substantially larger single‑point energy differences. Notably, after DFT optimization, the differences in free energy barriers between the generated and reference structures become much less prominent across all three datasets.

The practical value of UniTS-Gen lies in its ability to generate high-quality initial guesses that liberate chemists from manual trial-and-error. To verify this utility, we subjected the structures generated from the Formula-OOS test set, which represents a highly challenging and common scenario in practical mechanistic exploration, to DFT-level transition state optimization. Notably, even based on a single random generation without any post-hoc filtering or heuristic constraints, the geometric success rate for converging to a first-order saddle point reached 68.7%. Subsequent manual validation, which filtered out ineffective transition states (e.g., those involving mere group rotation), confirmed that 41.9% of all test cases represented the reactive transition state; all of these cases passed intrinsic reaction coordinate (IRC) validation, with detailed results provided in the Supplementary Information (detailed in Supplementary Note 13). The final success rate closely approaches that achieved during the construction of the UniTS-Lib dataset itself. Furthermore, multi‑sampling can substantially improve the success rate: for five previously failed Formula‑OOS cases, generating 10 samples per reaction led to all five yielding correct transition states upon DFT optimization, all of which passed IRC validation.

To further illustrate this practical utility, Fig. 4d presents three representative, structurally complex, and mechanistically important transition states from the Formula-OOS dataset: **TS1**, a hydrogen atom transfer transition state featuring an iron-porphyrin moiety; **TS2**, a Ru-catalyzed C–H activation transition state involving aromatic π-coordination; and **TS3**, a B–H bond activation transition state containing a complex icosahedral borane substructure. UniTS-Gen correctly generated chemically reasonable initial geometries for these challenging systems, accurately positioning the critical bond-forming/breaking distances essential for subsequent TS optimization. The model also successfully reproduced intricate substructures, such as the iron-porphyrin macrocycle and the icosahedral borane cluster. Crucially, all three generated structures were successfully optimized to correct transition states and verified as valid for the intended pathway.

These results on the highly challenging Formula-OOS test, which was drawn from UniTS-Lib and spans broad element types, a wide range of molecular sizes, and diverse reaction types, demonstrate the potentially universal nature of our architecture. By providing reliable starting points for complex reaction systems, UniTS-Gen addresses the most labor-intensive bottleneck in TS discovery, namely the manual trial‑and‑error construction of reasonable 3D initial guesses. As a key component in automating mechanistic exploration, these results serve as a definitive proof-of-concept for UniTS-Gen’s practical efficacy.

### Applications in kinetic selectivity analysis and mechanistic discovery

**Fig. 5:**
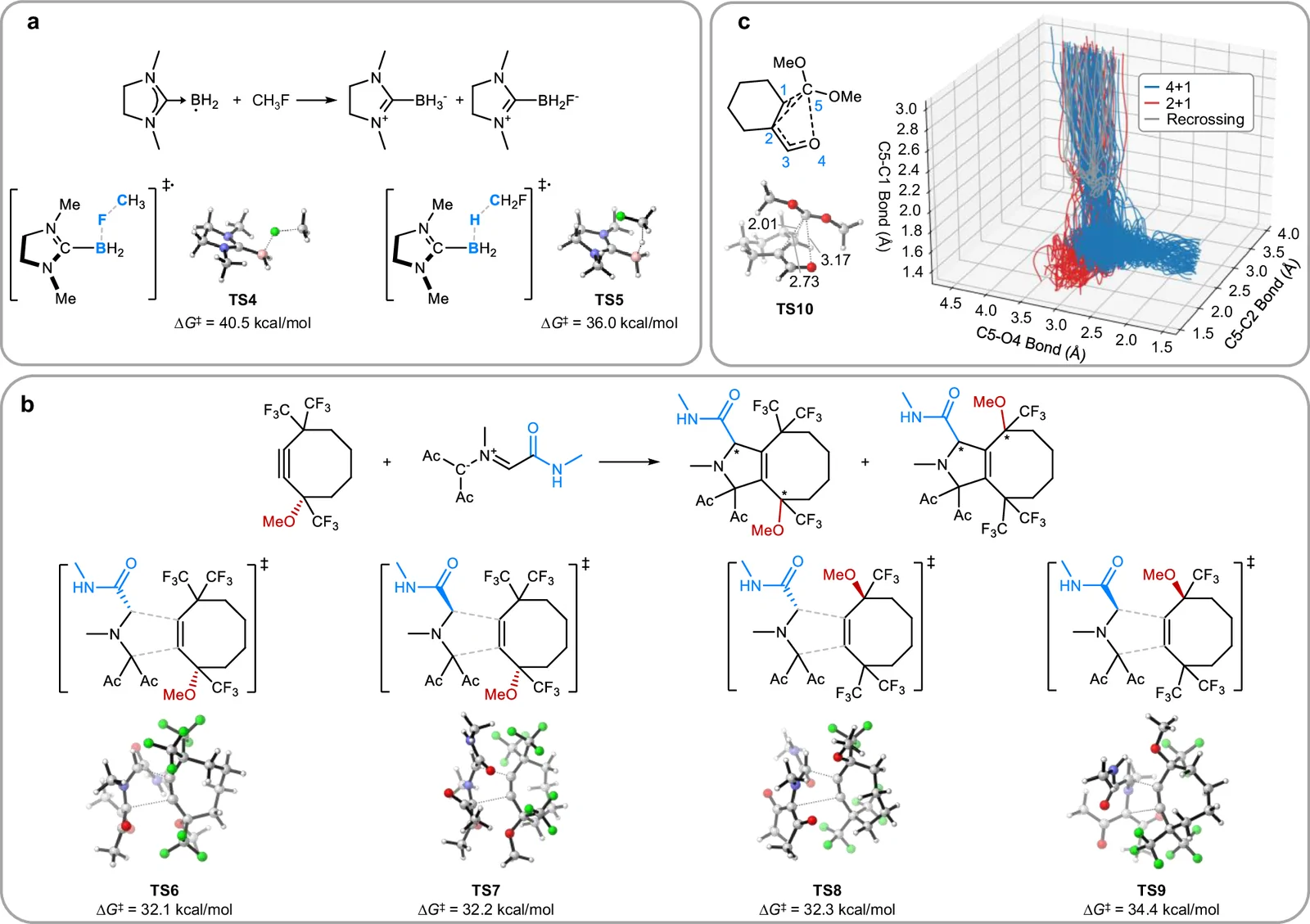
Demonstration of UniTS-Gen’s capability in reaction selectivity analysis, sampling complex stereochemically diverse space, and ambimodal TS analysis. **a** UniTS-Gen accurately predicts the kinetic selectivity in the C–H/C–F bond activation of CH_3_F by a Lewis base–boryl radical. By generating initial guesses for both pathways (**TS4** and **TS5**) guided by reactive site indices, subsequent DFT optimization yields free energies (*∆G*^‡^) consistent with the original report, correctly identifying the C–H activation pathway (*∆G*^‡^ = 36.0 kcal/mol) as kinetically favored over C–F activation (*∆G*^‡^ = 40.5 kcal/mol). **b** Utility in exploring complex stereochemical landscapes. UniTS-Gen successfully samples and generates initial guesses for all four possible diastereomeric TS configurations (**TS6**–**TS9**) in a complex [3 + 2] dipolar cycloaddition. Furthermore, the model’s conformational sampling power located a significantly lower energy barrier for the lowest-barrier stereoisomer (**TS6,** *∆G*^‡^ = 32.1 kcal/mol) compared to the literature dataset value (35.2 kcal/mol), highlighting the ability to accelerate high-precision mechanistic discovery. **c** Overlaid 3D trajectories from quasi‑classical molecular dynamics simulations of the ambimodal transition state. Blue, red, and gray indicate trajectories leading to the [4 + 1] cycloaddition product, the [2 + 1] cycloaddition product, and recrossing, respectively.

To further substantiate UniTS-Gen’s capability in kinetic and mechanistic studies, we first selected a highly selective bond activation system from the UniTS-Lib Formula-OOS test set, specifically the selective C–F or C–H bond activation of CH_3_F by a Lewis base–boryl radical, as reported by Xue et al.^34^ (Fig. 5a). Despite sharing the same reactant graph input, the two pathways yield distinct TS structures (**TS4** for C–F activation and **TS5** for C–H activation) differentiated solely by the reactive site indices provided as conditional inputs. For each site condition, we generated ten independent samples; all ten faithfully followed the conditioning to produce the expected TS geometries. Following structure generation by UniTS-Gen and subsequent DFT optimization using the computational methodology consistent with the original literature, we successfully located the saddle points for both **TS4** and **TS5** and confirmed them as correct transition states for their intended pathways. By comparing the free energies of these TS structures with the reactant, we obtained kinetic selectivities consistent with the reported findings, showing that the C–H bond activation pathway (*∆G*^‡^ = 36.0 kcal/mol) is kinetically favored over the C–F bond activation pathway (*∆G*^‡^ = 40.5 kcal/mol) promoted by the boryl radical. This case highlights how UniTS-Gen enables selectivity analysis by enabling the generation of distinct, path-specific transition states controlled solely by the input reaction center.

Furthermore, we demonstrated UniTS-Gen’s utility in exploring complex, stereochemically diverse systems by selecting a representative reaction from the test set of the [3 + 2] dipolar cycloaddition dataset^29^ (Fig. 5b), whose formula appeared only once in the entire dataset, making it a critical Formula-OOS case. Based on the same reactant molecular graph and reactive site indices, UniTS-Gen successfully sampled and generated initial guesses for all four possible diastereomeric TS configurations (**TS6**–**TS9**). Subsequent DFT optimization successfully located the reactive first-order saddle points for all generated structures. Among these, the lowest‑barrier configuration (**TS6**) corresponded to the stereoisomer originally reported in the literature. This result confirms UniTS-Gen’s ability to cover the stereochemically diverse space. This high-precision, broad-sampling approach is a substantial advantage over traditional manual or computationally expensive conformer searches, which are often limited by predefined templates, thereby significantly accelerating accurate mechanistic discovery.

To further explore the capability of UniTS-Gen in more complex mechanistic scenarios, we applied it to an ambimodal transition state system recently reported in the literature^35^, in which a single transition state bifurcates to give both [4 + 1] and [2 + 1] cycloaddition products. The elemental composition of this system was not present in UniTS-Lib, providing a rigorous test of generalization to a truly unseen chemical space. Using the molecular graph together with the C1–C5 atom pair index as input, we generated 10 TS guesses. Seven of these corresponded to the intended TS geometry, while the remaining three involved a different bond‑forming pattern (C5–C3). From the seven successful guesses, we selected a stable conformation for DFT optimization, which successfully converged to a correct transition state (**TS10** in Fig. 5c). Quasi‑classical molecular dynamics simulations based on **TS10** reproduced the bifurcation behavior, yielding both cycloaddition products (Fig. 5c). This result demonstrates that UniTS-Gen can generate accurate TS geometries for ambimodal systems, where precise bond lengths in the reactive region are critical, and extends the applicability of our framework to complex dynamic phenomena beyond conventional transition state analysis.

## Discussion

We establish a unified strategy for generating initial TS structures, integrating an automated workflow for dataset construction with a generative model based on higher-degree equivariant diffusion. This enabled the creation of UniTS-Lib, a library of over 4000 consistently optimized TS structures with broad elemental and structural diversity. To learn from this complex dataset, we developed UniTS-Gen, which employs a higher-degree equivariant denoising kernel (HiEGNN) to generate 3D TS geometries from only 2D reactant graphs.

UniTS-Gen demonstrated high accuracy across multiple benchmarks and robust generalization to the Formula-OOS set of UniTS-Lib, containing unseen elemental compositions. While challenges remain for structures with entirely unseen elements or sizes beyond the training set. DFT optimization of the generated structures on the challenging Formula-OOS test set yielded a 68.7% success rate for convergence to a first-order saddle point, with 41.9% verified by IRC as the correct TS. The framework’s capacity to capture subtle 3D geometric features, exemplified by the iron-porphyrin macrocycle and the icosahedral borane cluster in Fig. 4d, suggests potential for broader application, such as in predicting other complex architectures in materials science.

Furthermore, we demonstrated UniTS-Gen’s utility in kinetic selectivity analysis and mechanistic discovery. For selective C–H/C–F bond activation, specifying different reactive sites allowed UniTS-Gen to generate valid initial structures for both pathways, enabling the reproduction of reported selectivity after DFT optimization. In a complex [3 + 2] cycloaddition, the model sampled all potential diastereomeric TS configurations. In addition, we applied UniTS-Gen to an ambimodal transition state system, where a single TS bifurcates to give both [4 + 1] and [2 + 1] cycloaddition products.

These findings underscore that our proposed unified strategy significantly enhances the automation of organic reaction mechanism research. Specifically, UniTS‑Gen eliminates the most labor‑intensive and expert‑intuition‑dependent bottleneck in TS discovery by automating the construction of reasonable 3D initial guesses, thereby serving as an essential building block for automated mechanistic discovery. It should be emphasized that UniTS‑Gen requires reactive‑site indices as input. This design provides a flexible interface for chemists to generate transition states for specific reactive sites of interest on demand. For large‑scale, end‑to‑end automated reaction network exploration, reactive sites can be identified by rule‑based product enumeration frameworks^36^, or by heuristic rules. By providing a scalable and transferable solution, this approach is readily applicable to TS generation across organic synthesis. Furthermore, by continuously collecting TS structures from mechanistic studies using our automated data curation workflow, and by integrating other existing TS datasets^22,29,37^, we can systematically expand the training coverage. Given that HiEGNN has demonstrated its ability to denoise and generate accurate geometries for highly complex systems, this expansion will effectively address the current limitations on Element‑OOS and Maximum‑OOS tasks, moving us closer to a universal TS generation model.

Our work paves the way for next-generation computational tools, as the reliable initial guesses may enable future integration with machine learning potentials^38,39^ for end-to-end optimization and could provide critical 3D TS information for quantitative structure-selectivity modeling^40–42^, ultimately accelerating data-driven catalysis research. Furthermore, the HiEGNN demonstrates a robust capacity to generate accurate and intricate 3D structures even from sparse and complex data. This capability suggests its potential for extension to other challenging structure-generation tasks in computational chemistry that face similar data-sparsity constraints.

## Methods

### Data collection for UniTS-Lib

The TS structures comprising UniTS-Lib were harvested from the Supplementary Information of literature reports detailing organic reaction mechanisms. We developed a custom data curation pipeline for the automated extraction and standardization of 3D atomic coordinates from computational output files across 346 distinct literature sources. Detailed statistics regarding the journal sources and the 346 literature documents used to construct UniTS-Lib are provided in Supplementary Table 1. Transition state structures were collected from the Supplementary Information of published articles using PyMuPDF (fitz, v.1.26.7). Molecular structures were processed and standardized using RDKit (v.2025.03.5), MolOP (v.0.1.34.1), and Open Babel (openbabel-wheel, v.3.1.1.22). Data analysis was performed using Python 3.11 with NumPy (v.1.26.4), SciPy (v.1.16.1), Matplotlib (v.3.10.6), Pymatgen (v.2025.6.14), and RDKit (v.2025.3.5).

### Computational details for UniTS-Lib

All DFT calculation results were obtained using the Gaussian 09 program^43^. Geometry optimizations of all structures in UniTS-Lib were carried out using B3LYP functional^44,45^, employing the D3 version of Grimme’s dispersion corrections^46^ with Becke-Johnson damping^47^, and the Def2SVP basis set^48^ for all elements. Frequency analysis was performed at the same level of theory as geometry optimization to confirm whether optimized stationary points were either local minima or transition states.

### 2D molecular graph encoding in UniTS-Gen

The UniTS-Gen framework utilizes 2D molecular graphs as input. The input molecular graph includes nine node features: atom type, explicit atom degree, total charge, spin multiplicity, chiral tag, aromaticity, total valence, hydrogen count, and Cahn–Ingold–Prelog (CIP) code. It also includes five edge features: bond type, bond direction, stereo information, presence in a ring and conjugation status (Supplementary Fig. 5). These features are processed by a 2D graph encoder, adapted from our prior work^30^, which employs embedding layers followed by multiple graph convolutional blocks to generate latent 2D representations. To incorporate reaction-specific information, we extract a subgraph representing the reactive sites, which is encoded and concatenated with the global molecular embedding to form a unified molecular context for conditional denoising. Model development was performed using Python 3.11 with PyTorch (v.2.4.0), PyTorch Geometric (v.2.6.1), e3nn (v.0.5.7), NumPy (v.1.26.4), SciPy (v.1.16.1), and RDKit (v.2025.3.5).

### Higher-degree SE(3)-equivariant graph neural network in UniTS-Gen

An SE(3)-equivariant graph neural network is a graph-based model for three-dimensional molecular structures, in which atoms are treated as nodes and their interactions as edges for message passing. “SE(3)-equivariant” means that the representation remains physically consistent under rigid translations and rotations of the input geometry. The term “higher-degree” indicates that the model uses not only scalar (degree-0) and vector (degree-1) features, but also spherical harmonic features with degrees greater than 2, to encode finer directional structure and geometric coupling.

Higher-degree SE(3)-equivariant graph neural networks represent each node by a collection of SO(3) irreducible features spanning degrees $l=0,\ldots,{L}_{\max }$, rather than by scalar or vector features alone. For node i, the embedding is written as:

1 $${{{\bf{h}}}}_{i}={\left\{{{{\bf{h}}}}_{i}^{\left(l\right)}\right\}}_{l=0}^{{L}_{\max}},{{{\bf{h}}}}_{i}^{\left(l\right)} \in {R}^{\left(2l+1\right) \times C}$$

where C denotes the number of channels at each degree. Under a rotation $R\in {SO}\left(3\right)$, each degree transforms according to the corresponding Wigner-D matrix,

2 $${{{{\bf{h}}}}}_{i}^{\left(l\right)}\mapsto {{{{\rm{D}}}}}^{\left(l\right)}\left(R\right){{{{\bf{h}}}}}_{i}^{\left(l\right)}$$

At initialization, the embedding ${{{{\bf{h}}}}}_{i}^{\left(l\right)}$ is typically constructed from spherical harmonic encodings of the relative coordinates ${{{{\bf{r}}}}}_{{ij}}={{{{\bf{x}}}}}_{j}-{{{{\bf{x}}}}}_{i}$, or initialized as all-zero vectors. During message passing, higher-degree SE(3)-equivariant graph neural networks employ various equivariant operations to compute interactions among node embeddings, as well as between node embedding and the relative coordinate vectors. These operations include tensor products, gating mechanisms, and others. Through the composition of these equivariant functions, the entire model remains equivariant under arbitrary rotations.

### HiEGNN denoising kernel in UniTS-Gen

The core of UniTS-Gen is the HiEGNN denoising kernel that predicts time-step-specific noise for 3D coordinates. The kernel constructs a dynamic 3D molecular graph based on the current atomic positions and types, from which it derives higher-degree SO(3) embeddings through distance and degree embedding. These structural representations are fused with the 2D molecular context by TASFs and iteratively refined through multiple Transformer blocks comprising SO(2)-equivariant graph attention layers and feedforward networks (Supplementary Fig. 6). Finally, an equivariant prediction head processes these enhanced features to determine the noise, enabling the diffusion model to reconstruct transition state geometries directly from 2D graphs.

### Topology-augmented scalar features (TASFs) in UniTS-Gen

To enable the 2D molecular graph to guide 3D structure generation within the equivariant diffusion framework, we introduce topology-augmented scalar features (TASFs)—a mechanism that injects topological information into the spherical harmonic representations of HiEGNN. Rather than using raw 2D descriptors directly, the molecular graph is first encoded by a graph encoder^30^ to produce node-wise latent 2D features. When reaction-center conditioning is used, an additional reaction-center encoder generates a reactive-site representation, which is aggregated over the selected reactive atoms, broadcast to all nodes, and concatenated with the node-wise graph features to form the final 2D conditioning signal. During denoising, these conditioning features are further concatenated with the time embedding and passed through a learnable bridge module that projects them to match the dimensionality of the degree-0 (scalar) spherical components. These transformed features are then added directly to the degree-0 embeddings of each node in the dynamic 3D graph:

3 $${{{{\bf{h}}}}}_{i}^{0,{{{\rm{new}}}}}={{{{\bf{h}}}}}_{i}^{0,{{{\rm{old}}}}}+{{{\rm{Bridge}}}}\left({{{{\bf{x}}}}}_{i}^{2{{{\rm{D}}}}}\right)$$

where ${{{{\bf{h}}}}}_{i}^{0,{{{\rm{old}}}}}$ denotes the degree-0 embedding of node i before topological injection, ${{{{\bf{h}}}}}_{i}^{0,{{{\rm{new}}}}}$ denotes the updated degree-0 embedding after topological injection, and ${{{{\bf{x}}}}}_{i}^{2{{{\rm{D}}}}}$ is the corresponding 2D-derived node feature. This design ensures that the topological context—including molecular connectivity, atom types, and reactive site information—is directly infused into the lowest-order equivariant representation, which captures scalar-valued node-level information. By anchoring the degree-0 embeddings to the 2D graph priors, TASFs provide a strong inductive bias throughout the diffusion process, enabling the higher-degree components (*L* ≥ 1) to focus on resolving fine-grained 3D geometric details while remaining consistent with the topological constraints defined by the input graph.

### Diffusion model in UniTS-Gen

A diffusion model is a generative model that learns a data distribution through a pair of stochastic processes: a forward diffusion process and a reverse denoising process. In UniTS-Gen, diffusion is applied only to the three-dimensional coordinates, whereas atom identities, 2D graph representations and reaction-center context are retained as conditioning guidance throughout the process. Starting from the target transition-state geometry, Gaussian noise is progressively added to the coordinates, and the HiEGNN denoising kernel is trained to predict the noise at each step and reverse the corruption process.

Let ${{{\bf{x}}}}$ denote the target coordinates and c the conditioning information. In UniTS-Gen, the latent state at timestep t is written as ${{{{\bf{z}}}}}_{t}=\left[{{{{\bf{z}}}}}_{t}^{\left({{{\bf{x}}}}\right)},{{{\bf{h}}}}\right]$, where ${{{\bf{h}}}}$ denotes node-level conditioning features that are not diffused. The noise schedule is parameterized by a scalar function ${{{\rm{\gamma }}}}\left(t\right)$, from which

4 $${\alpha }_{t}=\sqrt{{{{\rm{sigmoid}}}}\left(-{\gamma }_{t}\right)},\,\,\,\,\,\,\,\,\,{\sigma }_{t}=\sqrt{{{{\rm{sigmoid}}}}\left({\gamma }_{t}\right)}$$

The forward diffusion step maps clean coordinates to noisy coordinates according to

5 $$q\left({{{{\bf{z}}}}}_{t} | {{{\bf{x}}}}\right)={{{\mathscr{N}}}}\left({{{{\bf{z}}}}}_{t};{\alpha }_{t}{{{{\bf{x}}}}}_{t},{\sigma }_{t}^{2}{{{\bf{I}}}}\right)$$

with

6 $${{{{\bf{z}}}}}_{t}^{\left({{{\bf{x}}}}\right)}={\alpha }_{t}{{{\bf{x}}}}+{\sigma }_{t}{{{\rm{\epsilon }}}},\,\,\,\,\,\,\,\,{{{\boldsymbol{\epsilon }}}}\sim {{{{\mathcal{N}}}}}_{{{{\rm{cg}}}}}\left(0,{{{\bf{I}}}}\right)$$

where ${{{{\mathscr{N}}}}}_{{{{\rm{cg}}}}}$ denotes Gaussian noise restricted to the zero-center-of-gravity subspace to preserve translation invariance. The complete latent state is

7 $${{{{\bf{z}}}}}_{t}=\left[{{{{\bf{z}}}}}_{t}^{\left({{{\bf{x}}}}\right)},{{{\bf{h}}}}\right]$$

In the reverse denoising process, the HiEGNN kernel predicts the noise at timestep t under conditional guidance c,

8 $$\hat{{{{{\boldsymbol{\epsilon }}}}}_{t}}=\phi \left({{{{\bf{z}}}}}_{t},t,c\right)$$

and the clean-coordinate estimate is recovered by the standard noise parameterization,

9 $$\hat{{{{\bf{x}}}}}=\frac{1}{{\alpha }_{t}}\left({{{{\bf{z}}}}}_{t}^{\left({{{\bf{x}}}}\right)}-{\sigma }_{t}\hat{{{{{\boldsymbol{\epsilon }}}}}_{t}}\right)$$

During training, the core objective is the mean-squared error between the sampled noise and the predicted noise.

10 $${{{{\mathcal{L}}}}}_{{{{\rm{diff}}}}}={E}_{t,{{{\bf{x}}}},{{{\boldsymbol{\epsilon }}}}}\left[{{{{\boldsymbol{||}}}}{{{\boldsymbol{\epsilon }}}}-\phi \left({{{{\bf{z}}}}}_{t},t,c\right){||}}^{2}\right]$$

The timestep t is sampled from the interval [0,T], where T corresponds to Gaussian noise. After training, the diffusion model $\phi \left(\cdot \right)$ can start from randomly sampled Gaussian noise and gradually recover the data distribution through multiple iterative sampling steps ($T\to T-1\to T-2\to \ldots \to 0$). For the single reverse transition from t to s=t−1, the implementation uses a conditional Gaussian distribution

11 $$p\left({{{{\bf{z}}}}}_{s},|,{{{{\bf{z}}}}}_{t},c\right)={{{\mathscr{N}}}}\left({{{{\bf{z}}}}}_{s};{\mu }_{t\to s},{\sigma }_{t\to s}^{2}{{{\bf{I}}}}\right)$$

with

12 $${\mu }_{t\to s}=\frac{{{{{\bf{z}}}}}_{t}^{(x)}}{{\alpha }_{{t|s}}}-\frac{{\sigma }_{{t|s}}^{2}}{{\alpha }_{{t|s}}{\sigma }_{t}}\hat{{{{{\boldsymbol{\epsilon }}}}}_{t}},{\sigma }_{t\to s}=\frac{{\sigma }_{{t|s}}{\sigma }_{s}}{{\sigma }_{t}},{\alpha }_{{t|s}}=\frac{{\alpha }_{t}}{{\alpha }_{s}}$$

### Metric for assessing structural clash (clash ratio)

To evaluate the chemical plausibility of the generated 3D transition state structures and identify physically implausible geometries, we defined a clash ratio metric. A structure is classified as a “clash” if any pair of atoms *i* and *j* exhibits an interatomic distance *d*_*ij*_ indicating severe steric compression or structural collapse. Specifically, a structural clash is identified based on the following criterion:

13 $${d}_{{ij}} < \,0.75\,\times \,({r}_{{{\mathrm{cov}}},i}+{r}_{{{\mathrm{cov}}},j})$$

where *r*_cov*,i*_ and *r*_cov*,j*_ represent the respective covalent radii of the atoms. The clash ratio is calculated as the percentage of generated structures within a given test set that contain at least one such physically implausible interatomic distance. This metric serves as a robust measure for assessing the structural integrity of generative models when applied to complex molecular systems.

### RMSD calculation

RMSD values were calculated following the same procedure used by Duan et al.^20^ Specifically, the generated transition-state structure was first aligned to the corresponding reference transition-state structure using the structure matching functionality implemented in Pymatgen (version 2025.6.14). The RMSD between the aligned atomic coordinates was then calculated and used to quantify the structural similarity between the generated and reference transition-state geometries.

### Reporting summary

Further information on research design is available in the Nature Portfolio Reporting Summary linked to this article.

## Supplementary information

Supplementary Information

Reporting Summary

Transparent Peer Review file

## Source data

Source Data

Source Data

## Data Availability

The UniTS-Lib dataset, the preprocessed Transition1x dataset, and the [3 + 2]-cycloaddition dataset used in this study have been deposited in Figshare: https://doi.org/10.6084/m9.figshare.31338676 (ref. ^49^). The IRC results generated in this study have been deposited in GitHub: https://github.com/licheng-xu-echo/UniTS. All data used and generated in this study are publicly available. Source data are provided with this paper.

## Code availability

Codes for data preprocessing, model training, generation and visualization of transition states are available via https://github.com/licheng-xu-echo/UniTS. A permanently archived version is accessible through Zenodo at https://doi.org/10.5281/zenodo.20586870 (ref. ^50^).
