# Supplementary material for: Automated transition state generation for mechanistic exploration in organic synthesis
Source: Nat Commun. 2026 Aug 27;17:10254. doi: 10.1038/s41467-026-77230-8 (PMC13616924; doi:10.1038/s41467-026-77230-8)
Supplement: Supplementary file 1 [file 41467_2026_77230_MOESM1_ESM.pdf]

Supplementary Information

Automated transition state generation for mechanistic exploration in organic synthesis

Li-Cheng Xu (徐丽成) [1,2*], Junyi An (安俊逸) [1,2], Weiqi Liu (刘蔚起) [1,3], Yun-Fei Shi (史云飞) [1,2], Chong-Lei Ji (吉崇磊) [4], Fenglei Cao (曹风雷) [1,2*], Yuan Qi (漆远) [1,3*]

[1]Shanghai Academy of Artificial Intelligence for Science, Shanghai, 200232, China

[2]Golab Inc., Shanghai, 200000, China

[3]Artificial Intelligence Innovation and Incubation Institute, Fudan University, Shanghai, 201203, China

[4]School of Physical Science and Technology, ShanghaiTech University, Shanghai, 200120, China

[*]Email: xulicheng@sais.org.cn; caofenglei@sais.org.cn; qiyuan@fudan.edu.cn

Table of Contents

Table of Contents 1

Suppl. Note 1. Literature sources and journal statistics for UniTS-Lib 2

Suppl. Note 2. Computational details 3

Suppl. Note 3. Validation procedure for optimized transition state structures in UniTS-Lib 4

Suppl. Note 4. Generation of 2D molecular graphs and identification of reactive sites 5

Suppl. Note 5. Representative transformation types and TS structures in UniTS-Lib 6

Suppl. Note 6. Element coverage of UniTS-Lib 7

Suppl. Note 7. Data fields and content of UniTS-Lib entries 9

Suppl. Note 8. Detailed architecture of UniTS-Gen 10

Suppl. Note 9. Metric for assessing structural clash (clash ratio) 12

Suppl. Note 10. Detailed performance evaluation of UniTS-Gen under multi-sample inference 13

Suppl. Note 11. RMSD distribution and structural examples 15

Suppl. Note 12. Correlation between structural deviation and energy barrier 18

Suppl. Note 13. Detailed evaluation of UniTS-Gen on the Formula-OOS test set 20

Suppl. Note 14. Code and data availability 22

Supplementary references 23

Suppl. Note 1. Literature sources and journal statistics for UniTS-Lib

The initial set of transition state (TS) structures constituting UniTS-Lib was derived from the Supplementary Information documents of peer-reviewed articles focusing on detailed organic reaction mechanistic studies. Our comprehensive data curation pipeline involved the automated extraction and standardization of atomic coordinates from the computational files provided in 346 distinct literature sources.

Supplementary Table 1 summarizes the journal source statistics for all 346 literature documents used to construct UniTS-Lib. Detailed information regarding the source documents' digital object identifiers (DOI) and the script used for reaction coordinate extraction are available in our public GitHub repository: https://github.com/licheng-xu-echo/UniTS.

Supplementary Table 1 | Detailed list of journals for Supplementary Information documents.

Journal	Count	Journal	Count
Advanced Synthesis & Catalysis	1	ACS Catalysis	34
Asian Journal of Organic Chemistry	3	Journal of the American Chemical Society	84
Angewandte Chemie International Edition	72	JACS Au	1
Chemistry - An Asian Journal	5	Organometallics	1
ChemCatChem	1	Nature Catalysis	1
Chemistry – A European Journal	7	Nature Synthesis	2
Chinese Journal of Chemistry	1	Physical Chemistry Chemical Physics	3
ChemPlusChem	1	Chemical Science	13
European Journal of Organic Chemistry	2	Organic Chemistry Frontiers	8
Journal of Computational Chemistry	1	Chemical Communications	7
Science China Chemistry	3	Catalysis Science & Technology	1
Chem – A Cell Press journal	5	Dalton Transactions	4
Cell Reports Physical Science	1	Organic & Biomolecular Chemistry	1
Inorganic Chemistry	4	Inorganic Chemistry Frontiers	1
The Journal of Organic Chemistry	32	Science	1
Organic Process Research & Development	1	CCS Chemistry	5
Organic Letters	38	Molecules	1

Suppl. Note 2. Computational details

All density functional theory (DFT) calculation results were obtained using the Gaussian 09 program[1]. Geometry optimizations of all structures in UniTS-Lib were carried using B3LYP functional[2,3], employing the D3 version of Grimme's dispersion corrections[4] with Becke-Johnson damping[5], and the Def2SVP basis set[6] for all elements. Frequency analysis was performed at the same level of theory as geometry optimization to confirm whether optimized stationary points were either local minimum or transition states. Intrinsic Reaction Coordinate (IRC) calculations were not performed for the TS structures in UniTS-Lib during dataset construction.

For the geometry optimizations of the structures presented in Fig. 5 of the main text, the Gaussian 09 program was utilized, employing computational methods consistent with the original literature[7–9] for each respective structure, in order to optimize the local minimum or transition states and calculate the kinetic barriers. Furthermore, regarding the transition state (and the two reactants) from the original dataset related to the reaction discussed in Fig. 5b of the main text, we re-optimized them using the Gaussian 09 program and the exact computational method cited in the corresponding literature[7]. This was followed by frequency analysis and higher-level single point energy calculation to obtain kinetic barrier data directly comparable to the model generated transition state structure. Quasi-classical molecular dynamics simulations for Fig. 5c of the main text were performed at the M06-2X/def2-SVP-SMD(toluene) level of theory with TS structures optimized at the same level, using the Progdyn code developed by Singleton and coworkers[10]. Temperatures were set to 384 K for simulations with a time step of 1.0 fs. Trajectories were terminated when the forming bond lengths are either lower than 1.6 Å or longer than 3.0 Å or the simulation time is over 500 fs.

The 3D diagrams of molecules were generated using CYLView[11].

Suppl. Note 3. Validation procedure for optimized transition state structures in UniTS-Lib

Rigorous validation was applied to all optimized structures to ensure they represent true transition states, guaranteeing the high-quality of the UniTS-Lib dataset. This validation process involved a two-step approach:

1. Initial structural screening was conducted using frequency vibrations, retaining the structures that possessed one and only one imaginary vibrational frequency. This criterion is the necessary condition for a structure to be a true first-order saddle point on the potential energy surface.

2. To further confirm the identity of the transition state and verify that the imaginary frequency corresponds to the desired reaction coordinate (i.e., bond formation or cleavage), a graph-matching verification step was implemented. This process involves: (1) Displacing the optimized TS coordinates along the imaginary frequency mode, both in the forward and reverse directions, to generate two distinct terminal 3D structures. (2) Generating the 2D molecular graph representation for each of these two terminal 3D structures. (3) Comparing the connectivity of the two resulting 2D molecular graphs. A genuine reaction TS should show a change in connectivity (e.g., formation or cleavage of bonds) between the two terminal structures. (4) Structures where the two terminal 2D molecular graphs are identical (e.g., a TS corresponding solely to the rotation of a substituent group) were explicitly discarded as non-reactive saddle points. Related scripts are available in our public GitHub repository: https://github.com/licheng-xu-echo/UniTS.

Finally, ambiguous structures or cases where the automated verification yielded uncertain results were subjected to additional manual inspection to ensure absolute confidence in the final dataset inclusion.

Suppl. Note 4. Generation of 2D molecular graphs and identification of reactive sites

As illustrated in Supplementary Figure 1, we first utilize the MolOP[12] and RDKit[13] cheminformatics python package to derive the 2D molecular graph representation, complete with bond connectivity information, directly from the 3D optimized coordinates of the TS structure.

Subsequently, the molecular graph is used in conjunction with the TS's calculated imaginary vibrational frequency mode to locate the atoms involved in bond forming and bond breaking. These atoms collectively define the reactive site indices, which are provided as a crucial conditional input to the UniTS-Gen model to guide the 3D generation process towards the correct reaction center.

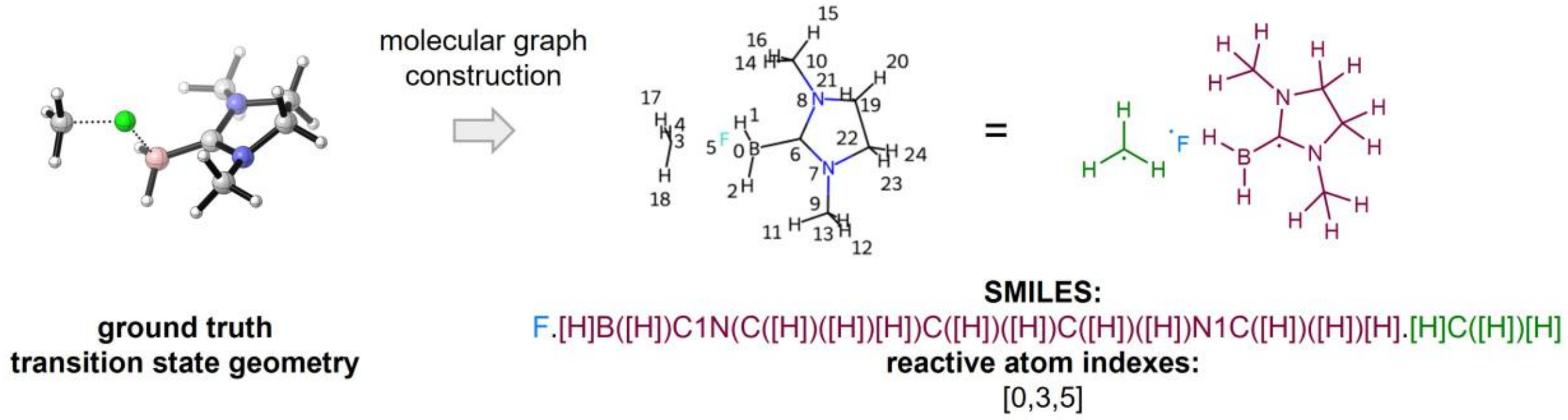

Supplementary Figure 1 | Generation of 2D molecular graph from ground truth TS geometry.

Suppl. Note 5. Representative transformation types and TS structures in UniTS-Lib

As the TS structures in UniTS-Lib were extracted and validated from a wide range of mechanistic literature, the dataset encompasses a diverse collection of chemical transformation types. Supplementary Figure 2 displays the corresponding TS structures for 12 of these representative chemical transformations.

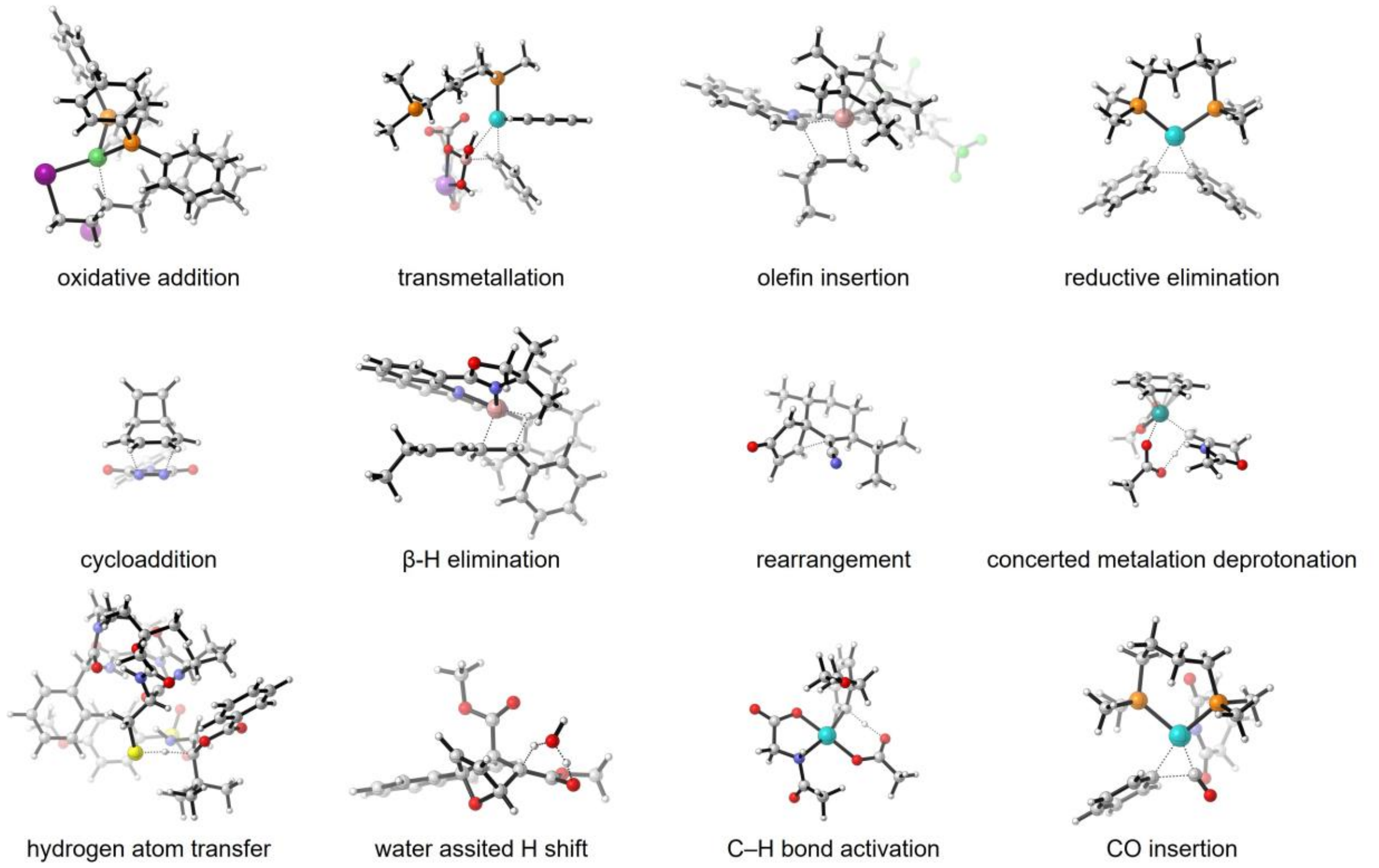

Supplementary Figure 2 | 12 representative transformation types and corresponding TS structures in UniTS-Lib.

Suppl. Note 6. Element coverage of UniTS-Lib

Supplementary Figure 3 displays a heatmap of the periodic table, which summarizes the elemental coverage of UniTS-Lib and records the occurrence frequency for each element. The counting methodology employed for this statistic tallies the presence of an element in a TS structure: if a TS structure contains a specific element, that element's count is incremented by one, regardless of the number of atoms of that element present in the structure. This provides a clear representation of the range of chemical spaces included in the dataset, encompassing common organic elements as well as various transition metals and heavier main group elements crucial for catalysis and complex synthesis.

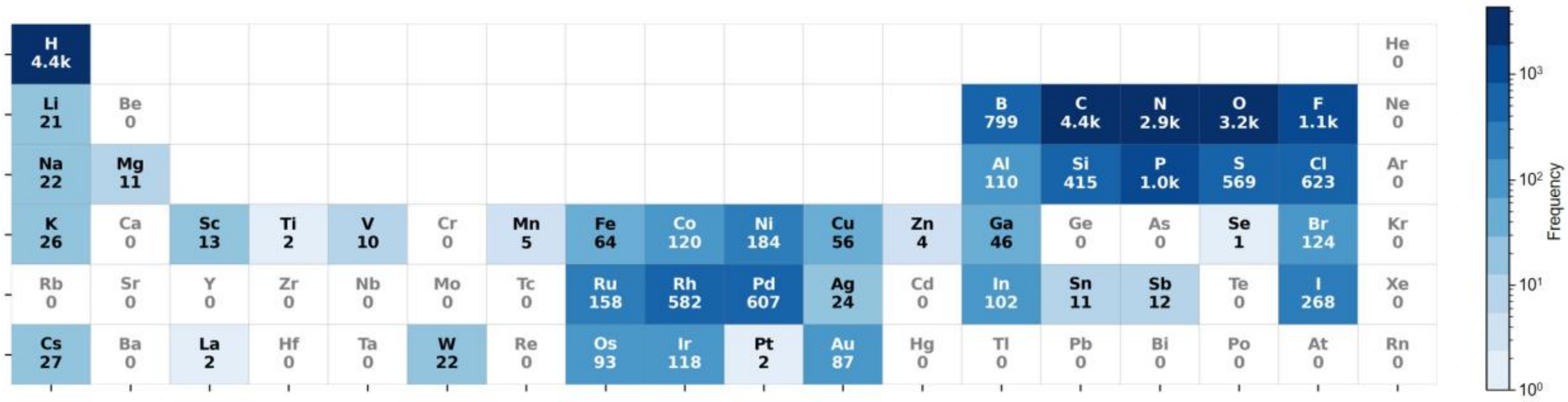

Supplementary Figure 3 | The heatmap of element coverage of UniTS-Lib.

To provide a detailed overview of element co-occurrence frequencies in UniTS-Lib, we include four chord diagrams (Supplementary Figure 4) covering the frequency ranges 1–10, 10–10^2, 10^2–10^3, and 10^3–10^4. In each diagram, the outer track length represents element frequency, and chord thickness scales with co-occurrence frequency. These diagrams collectively illustrate the distribution of element pairs across different frequency regimes.

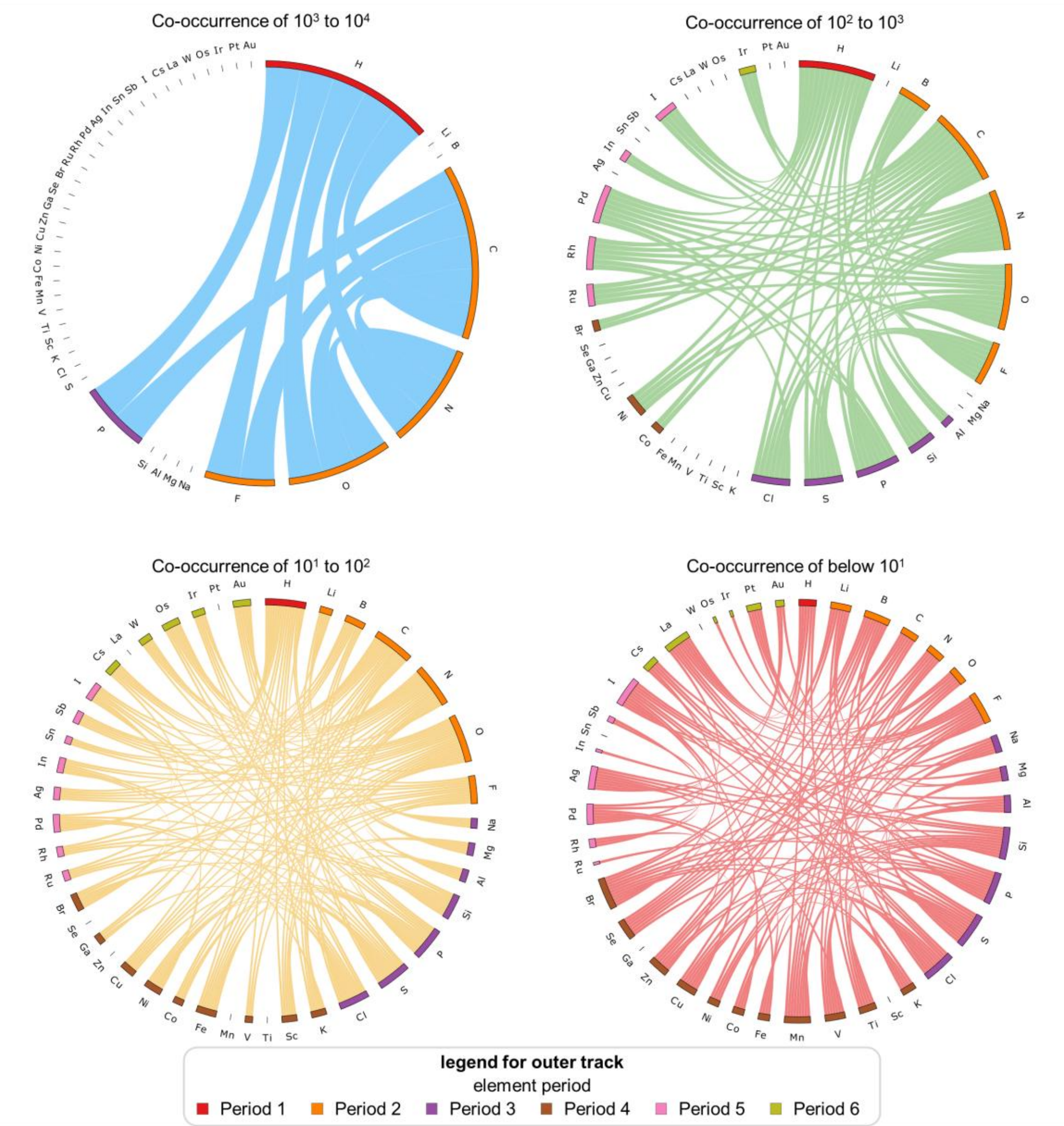

Supplementary Figure 4 | Element co-occurrence chord diagrams for UniTS-Lib across four frequency ranges.

Suppl. Note 7. Data fields and content of UniTS-Lib entries

Each TS structure within the UniTS-Lib is stored as a comprehensive data entry, ensuring sufficient information is available for both model training and downstream quantum chemistry analysis. This standardized data format includes essential structural and electronic properties derived from the rigorous DFT optimization and frequency analysis workflow.

Supplementary Table 2 lists and describes the complete set of data fields included in each UniTS-Lib entry. These fields encompass the optimized coordinates, electronic states (charge and multiplicity), key thermodynamic corrections (e.g., thermal correction to Gibbs free energy), and the calculated vibrational frequency mode necessary for confirming the saddle point identity. Specifically, the recorded forces correspond to the input geometry orientation (*inpt_orien*), while the vibration normal modes (*modes*) correspond to the standardized geometry orientation (*std_orien*). Additionally, the dataset includes the RDKit-generated molecular graph (*rdmol*); it is important to note that while this object retains 3D conformational information, our model exclusively utilizes its 2D topological information (*graph_feat*) and strictly ignores the embedded 3D coordinates. The UniTS-Lib is available in https://github.com/licheng-xu-echo/UniTS.

Supplementary Table 2 | Data fields and content of each UniTS-Lib record.

Label	Description	Units	Typing
atoms	atomic numbers (N×1)	–	list
inpt_orien	input orientation of geometry (N×3)	Å	array
std_orien	standard orientation of geometry (N×3)	Å	array
forces	forces (N×3)	Hartree / Å	array
energy	potential energy	Hartree	float
freqs	vibrational frequencies	cm^{-1}	array
modes	vibration normal modes	Å	array
TCH	thermal correction to enthalpy	Hartree / particle	float
TCG	thermal correction to Gibbs_free energy	Hartree / particle	float
chrg	net charge	–	integer
mult	spin multiplicity	–	integer
rdmol	molecular graph generated by RDKit	–	Mol object
rct_idx	reactive atom indices	–	list
graph_feat	node features and edge features of graph	–	tensor

Suppl. Note 8. Detailed architecture of UniTS-Gen

UniTS-Gen is constructed as a conditional diffusion model that leverages a higher-degree SE (3)-equivariant graph neural network (i.e., HiEGNN) for the denoising process. The model's primary input consists only of the transition state reactant molecule's 2D molecular graph and the atomic indices defining the reactive site.

During the inference sampling process, the atomic composition of the input molecular graph is used to initialize the 3D TS structure, where the coordinate components are initialized as random noise. Simultaneously, the 2D molecular graph and the reactive site indices are processed by the 2D graph encoder (detailed in Supplementary Figure 5) to generate a 2D molecular graph embedding. This embedding serves as the essential molecular context used to guide the HiEGNN in denoising the noisy coordinates.

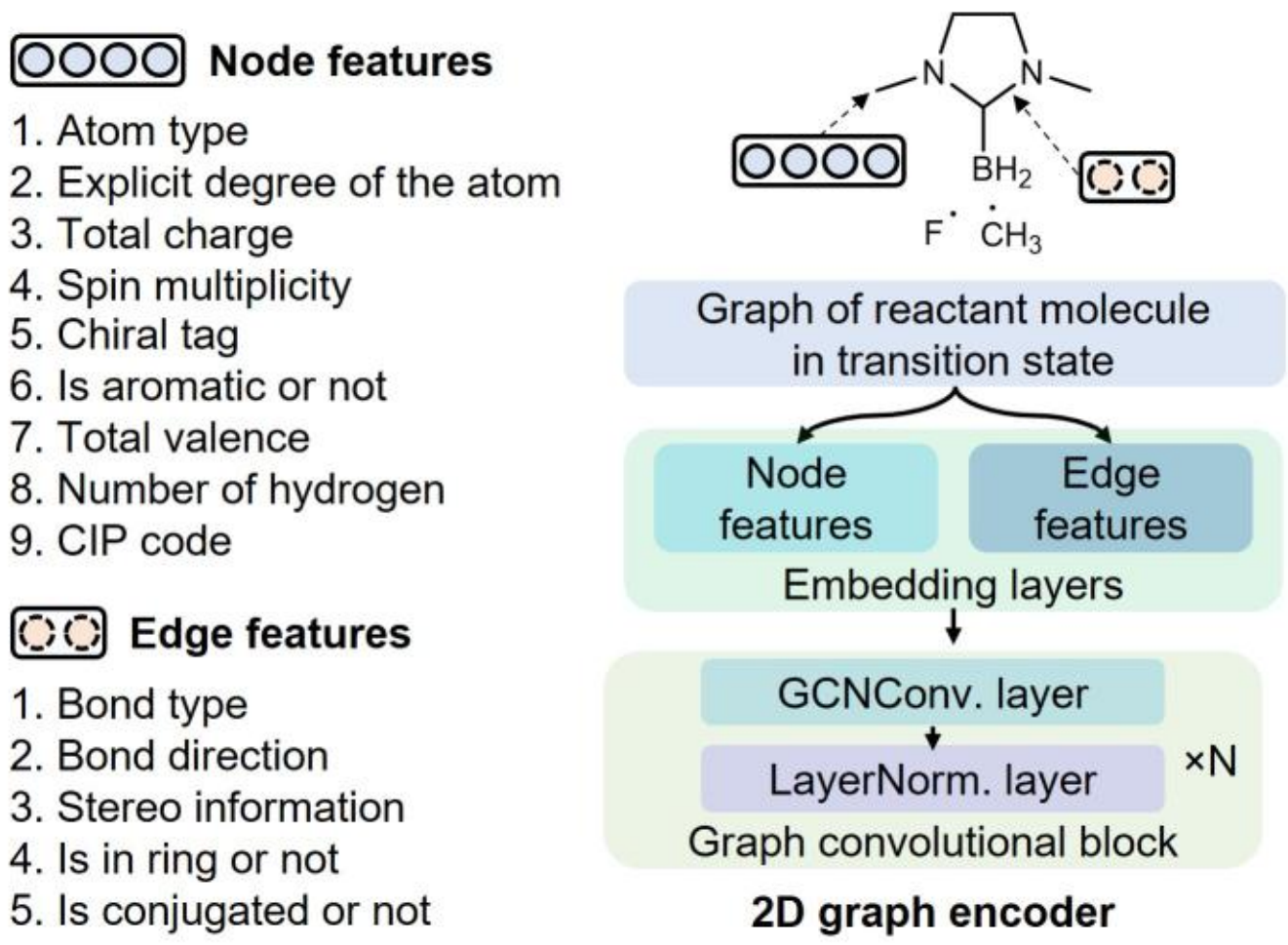

Supplementary Figure 5 | The node features and edge features generated for each 2D molecular graph and the detailed modules in 2D graph encoder.

Supplementary Figure 5 illustrates the 2D graph encoder architecture, which is adapted from our prior work[14]. The input molecular graph includes nine node features and five edge features, which are transformed into node and edge embeddings through embedding layers. Subsequently, these embeddings undergo multiple graph convolutional blocks, each composed of a GCN convolutional layer and a Layer Norm layer, to generate the final 2D graph embeddings. For the reactive site information, the relevant atoms are used to construct a subgraph, which is processed by the same 2D graph encoder to generate its corresponding embedding. The embeddings from the reactant molecular graph and the reactive site are concatenated to form the final molecular context used for conditional denoising.

In the model's training process, noise is progressively added to the ground truth TS structure over time steps. The HiEGNN is trained to predict the magnitude of this noise at the current time step, enabling the reverse diffusion process to revert the noisy TS coordinates back to the initial TS structure.

The core of UniTS-Gen is the HiEGNN denoising kernel, whose details are shown in Supplementary Figure 6. This architecture is adapted from the work by Liao et al.[15] The model receives three main inputs: atomic types, the positions at the current time step, and the molecular context derived from the 2D graph.

The HiEGNN first computes the molecular graph of the current 3D structure based on atomic types and positions, obtaining the edge indices. It then calculates the edge distance embedding and the edge degree embedding to derive the higher-degree SO(3) embeddings. Furthermore, the model extracts the element

embedding based on the atomic types, which is then concatenated with the molecular context from the 2D graph and added to the 0th degree (L_0) of the SO(3) embedding.

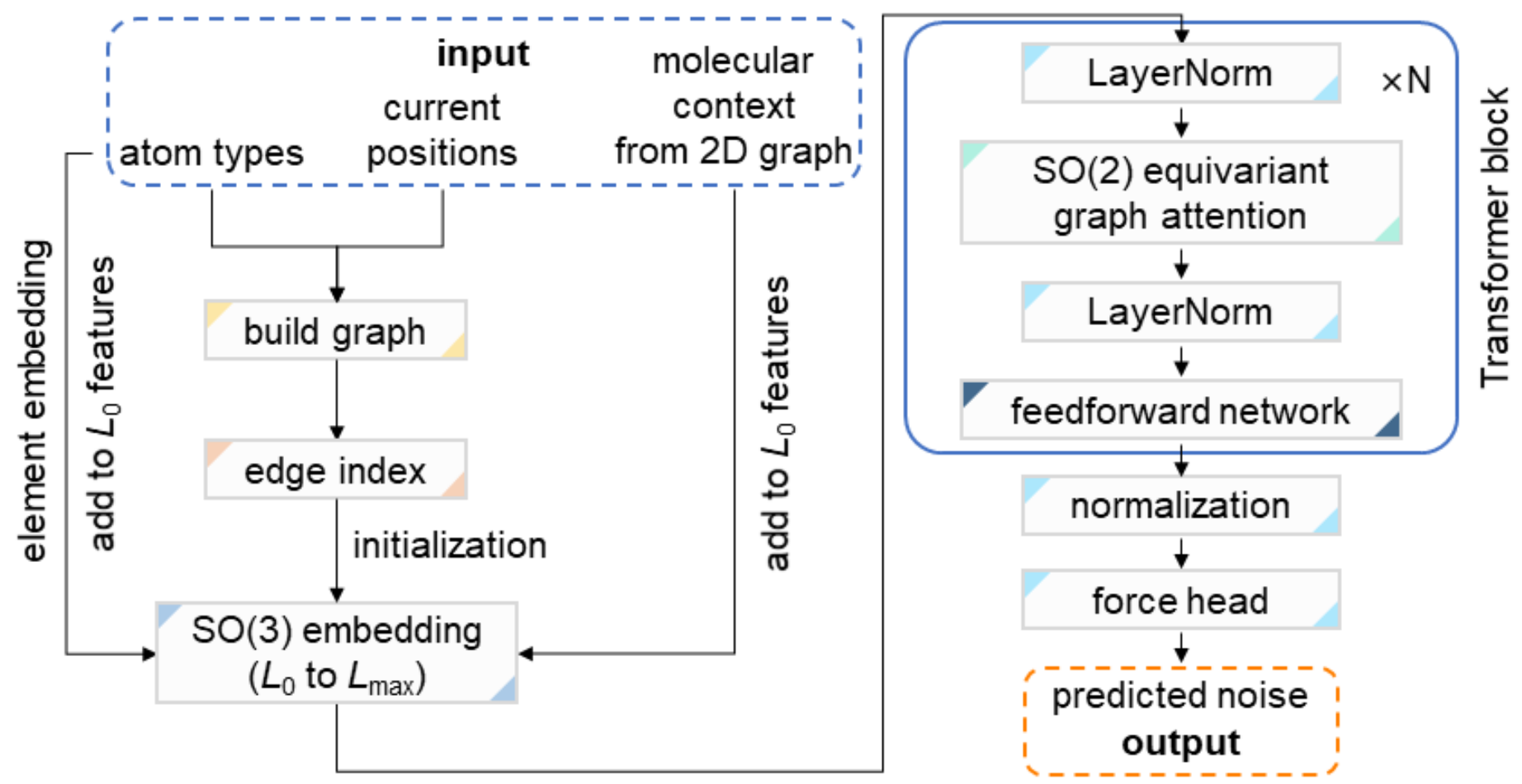

Supplementary Figure 6 | The detailed architecture of HiEGNN denoising kernel.

The SO(3) embeddings are then passed through multiple Transformer blocks, each comprising a Layer Norm layer, an SO(2) equivariant graph attention layer, another layer norm layer, and a feedforward network. These blocks enhance the representation capability of the structural information. Finally, the features are passed through a normalization layer. The original model's force prediction head (composed of SO(2) equivariant graph attention) is utilized to process these features to predict the noise corresponding to the current time step and position.

Suppl. Note 9. Metric for assessing structural clash (clash ratio)

To quantitatively assess the chemical validity of the generated 3D transition state structures and identify physically implausible geometries, we introduced the clash ratio metric. A generated structure is designated as a "clash" if any pair of atoms exhibits an interatomic distance that is significantly shorter than expected, indicating severe steric compression or structural collapse. Specifically, the criterion for identifying a structural clash is defined as follows: for any two atoms i and j, if the distance between them, d_{ij}, is less than 75% of the sum of their respective covalent radii ($r_{\mathrm{cov},i} + r_{\mathrm{cov},j}$), the structure is flagged as invalid.

$$d_{ij} < 0.75 \times (r_{\mathrm{cov},i} + r_{\mathrm{cov},j})$$

The clash ratio is subsequently calculated as the percentage of generated structures that contain at least one such physically implausible interatomic distance within a given test set. This metric provides a robust measure for evaluating the chemical plausibility of generative models on complex molecular systems.

Suppl. Note 10. Detailed performance evaluation of UniTS-Gen under multi-sample inference

This section presents the detailed quantitative performance metrics for the UniTS-Gen model, comparing the HiEGNN kernel against the baseline EGNN kernel, across various datasets and rigorous out-of-sample (OOS) test scenarios. All evaluations consider multi-sample inference, where the minimum root mean square deviation (RMSD) and minimum clash ratio are reported from 1, 5, and 10 generated samples. The RMSD is calculated after optimal alignment[16], and the clash ratio quantifies the percentage of structures with chemically implausible interatomic distances (detailed in section 9).

Supplementary Table 3 summarizes the performance of UniTS-Gen (both EGNN and HiEGNN kernels) on the Transition1x dataset's randomly split test set. The dataset was partitioned with a 8000:1000:1073 ratio for the training, validation, and test sets, respectively. The table reports the minimum RMSD and minimum clash ratio achieved from 1, 5, and 10 inference samples.

Supplementary Table 3 | Performance of UniTS-Gen (EGNN and HiEGNN kernels) on the Transition1x random test set. The best performance is shown in bold.

	1-sample		5-sample		10-sample	
	RMSD / Å	clash ratio	RMSD / Å	clash ratio	RMSD / Å	clash ratio
UniTS-Gen (EGNN)	0.3170	0.37%	0.1958	0.09%	0.1630	0.00%
UniTS-Gen (HiEGNN)	**0.2517**	**0.00%**	**0.1202**	**0.00%**	**0.0905**	**0.00%**

We compared UniTS-Gen (HiEGNN) with representative state-of-the-art methods, namely TSDiff[17], OA-ReactDiff[18], and React-OT[16], on the Transition1x test set. The comparison includes input type, accuracy (mean of RMSD), and computational efficiency (inference time per reaction), as summarized in Supplementary Table 4.

Supplementary Table 4 | Comparison of different models in terms of input type, accuracy, and computational efficiency.

Method	Input type	Mean of RMSD (Å)	Inference per reaction (s)
TSDiff	2D graph	0.2526	-
OA-ReactDiff (1 times)	3D coordinate	0.1800	6.80
OA-ReactDiff (40 times)	3D coordinate	0.1297	272.00
React-OT	3D coordinate	0.1029	0.39
React-OT + pretraining	3D coordinate	0.0981	0.39
UniTS-Gen (HiEGNN, 1 times)	2D graph	0.2517	0.45
UniTS-Gen (HiEGNN, 10 times)	2D graph	0.0905	4.5

Inference times for UniTS-Gen were measured on a single NVIDIA H800 GPU with a batch size of 128, averaged over the entire Transition1x test set. Direct comparison of different methods may be affected by differences in GPU specifications and batch size settings.

For the more complex UniTS-Lib Formula-OOS dataset, the average inference time per single sample is approximately 10.22 seconds under the same hardware configuration.

Due to GPU parallelism, generating multiple samples for one reaction adds only modest computational cost. For example, generating 1 or 10 samples for a single reaction can both be completed within a single inference batch using a batch size of 16.

Supplementary Table 5 presents the generation accuracy on the randomly split test set of the [3+2] dipolar cycloaddition dataset. The training, validation, and test sets were created using a 4215:526:528 ratio. The metrics reported are the minimum RMSD and minimum clash ratio from 1, 5, and 10 inference samples.

Supplementary Table 5 | Performance of UniTS-Gen (EGNN and HiEGNN kernels) on the [3+2] dipolar cycloaddition dataset random test set. The best performance is shown in bold.

	1-sample		5-sample		10-sample	
	RMSD / Å	clash ratio	RMSD / Å	clash ratio	RMSD / Å	clash ratio
UniTS-Gen (EGNN)	1.2016	10.61%	0.9680	0.76%	0.8588	0.19%
UniTS-Gen (HiEGNN)	**1.0757**	**0.38%**	**0.7195**	**0.00%**	**0.6159**	**0.00%**

Supplementary Table 6 details the performance of UniTS-Gen on the randomly split test set of the UniTS-Lib dataset. The dataset was partitioned into training (3512), validation (439), and test (440) sets. The reported metrics reflect the lowest RMSD and clash ratio across 1, 5, and 10 generated samples.

Supplementary Table 6 | Performance of UniTS-Gen (EGNN and HiEGNN kernels) on the UniTS-Lib dataset random test set. The best performance is shown in bold.

	1-sample		5-sample		10-sample	
	RMSD / Å	clash ratio	RMSD / Å	clash ratio	RMSD / Å	clash ratio
UniTS-Gen (EGNN)	1.3408	66.36%	1.1705	43.64%	1.1183	37.73%
UniTS-Gen (HiEGNN)	**1.0665**	**3.86%**	**0.7871**	**0.45%**	**0.7009**	**0.45%**

Supplementary Table 7 provides the evaluation metrics for the UniTS-Gen (HiEGNN kernel) on three rigorous OOS test sets derived from UniTS-Lib, which were specifically designed to test the model's generalization capabilities beyond the training domain. The Formula-OOS test set (403 structures) was constructed by grouping TS structures based on their unique atomic formula, thereby ensuring that all formulas in this test set were entirely unseen during training, with the remaining data randomly split into training (3589) and validation (399) sets. For the Element-OOS test, the test set (351 structures) comprises all TS structures in UniTS-Lib that contain elements from the 6th period of the periodic table, with the remaining structures randomly allocated to the training (3636) and validation (404) sets. Finally, the Maximum-OOS test set (219 structures) consists of all TS structures with a total atom count greater than 120, and the remaining data were randomly partitioned into training (3754) and validation (418) sets. The Supplementary Table 7 reports the minimum RMSD and minimum clash ratio from 1, 5, and 10 inference samples for the HiEGNN kernel on these three challenging OOS test sets.

Supplementary Table 7 | Performance of UniTS-Gen (HiEGNN kernels) on the UniTS-Lib dataset OOS test set.

	1-sample		5-sample		10-sample	
	RMSD / Å	clash ratio	RMSD / Å	clash ratio	RMSD / Å	clash ratio
Formula-OOS	1.1462	1.49%	0.8651	0.99%	0.7685	0.25%
Element-OOS	1.4113	72.08%	1.1678	41.88%	1.0821	30.20%
Maximum-OOS	1.6490	51.60%	1.5263	14.16%	1.4956	5.02%

Suppl. Note 11. RMSD distribution and structural examples

To illustrate how RMSD values correspond to structural differences across datasets, we selected three generated structures (single sample per reaction) from each test set, representing low, medium, and high RMSD relative to the reference geometry. These examples are shown in Figs. S7–S9, where panel a presents the RMSD histogram of generated structures on the respective test set, and panel b shows the overlay of generated (pink) and reference (blue) structures for the three RMSD levels.

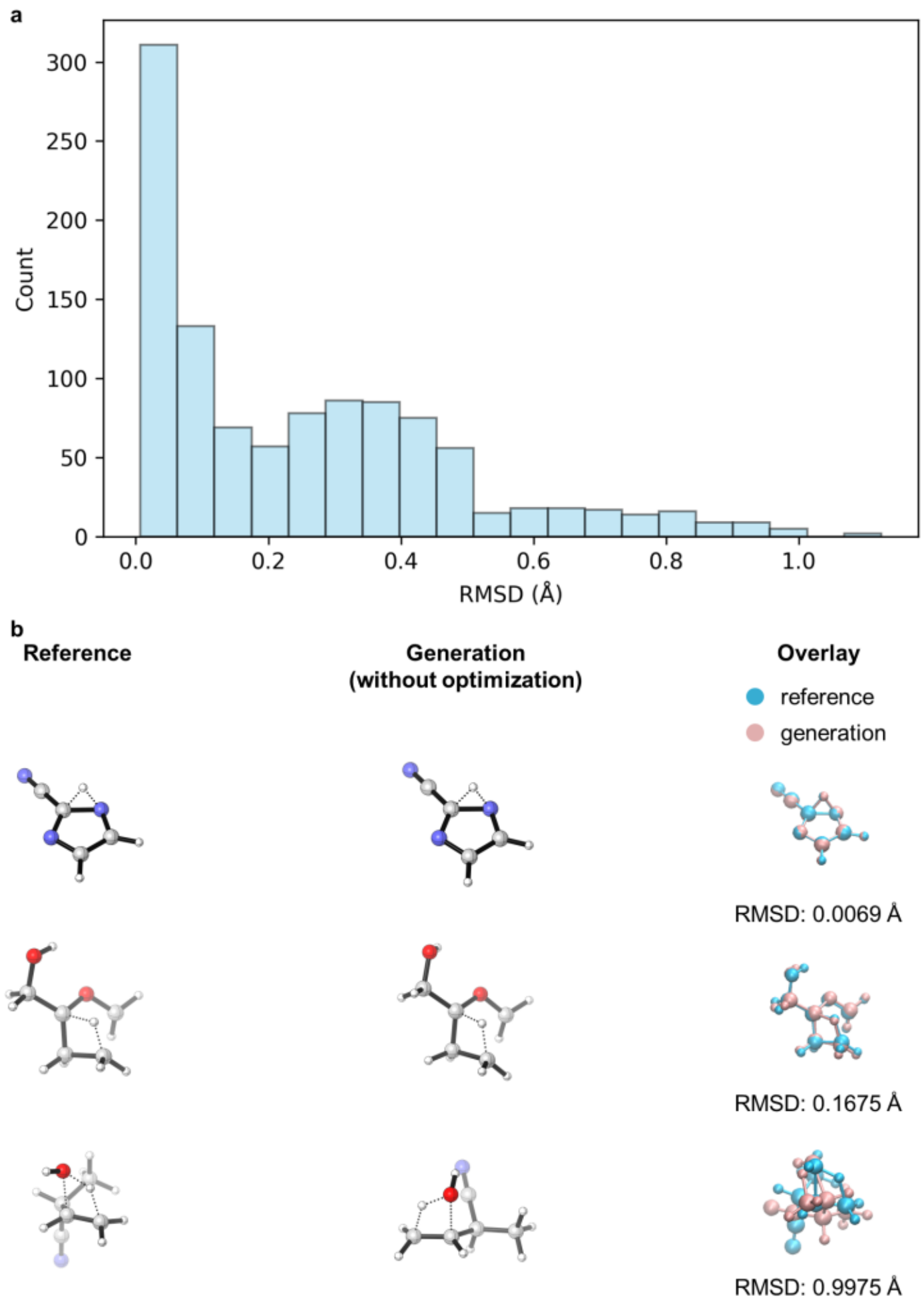

Supplementary Figure 7 | RMSD distribution and structural examples from the Transition1x test set. **a,** Histogram of RMSD values for generated TS structures. **b,** Overlay of generated (pink) and reference (blue) structures for representative cases with low, medium, and high RMSD.

For the Transition1x test set (Supplementary Figure 7), which contains the smallest molecules among three datasets, the selected RMSD values are 0.0069 Å, 0.1675 Å, and 0.9975 Å. The generated structures corresponding to these RMSD values are overlaid with their reference geometries in Supplementary Figure 7b.

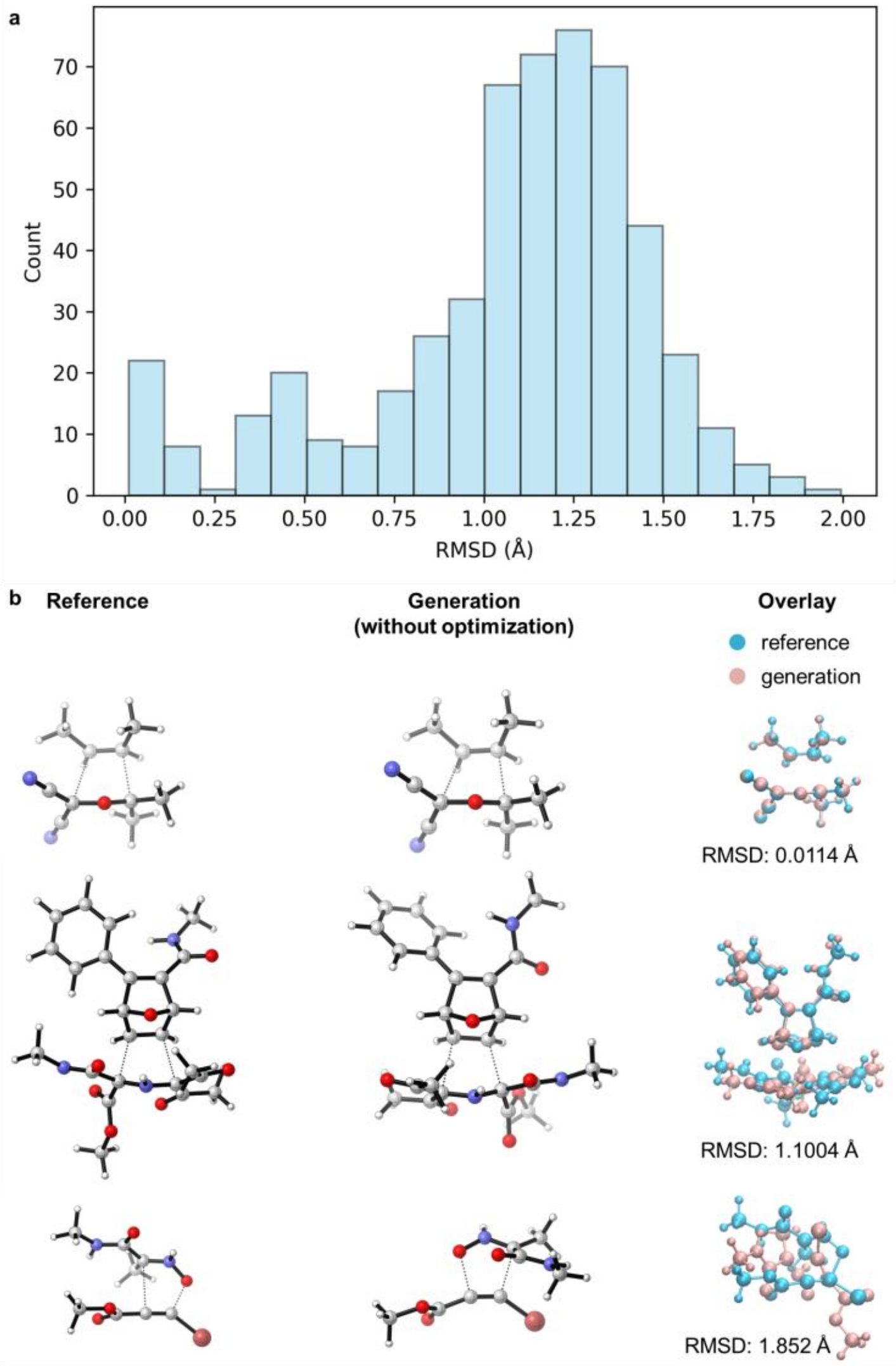

Supplementary Figure 8 | RMSD distribution and structural examples from the [3+2] cycloaddition test set. a, Histogram of RMSD values for generated TS structures. **b,** Overlay of generated (pink) and reference (blue) structures for representative cases with low, medium, and high RMSD.

For the [3+2] cycloaddition test set (Supplementary Figure 8), the RMSD values of selected molecules are 0.0114 Å, 1.1004 Å, and 1.852 Å. The RMSD histogram (Supplementary Figure 8a) shows a broad distribution centered around 1.25 Å. The overlaid structures for the three RMSD levels are shown in Supplementary Figure 8b.

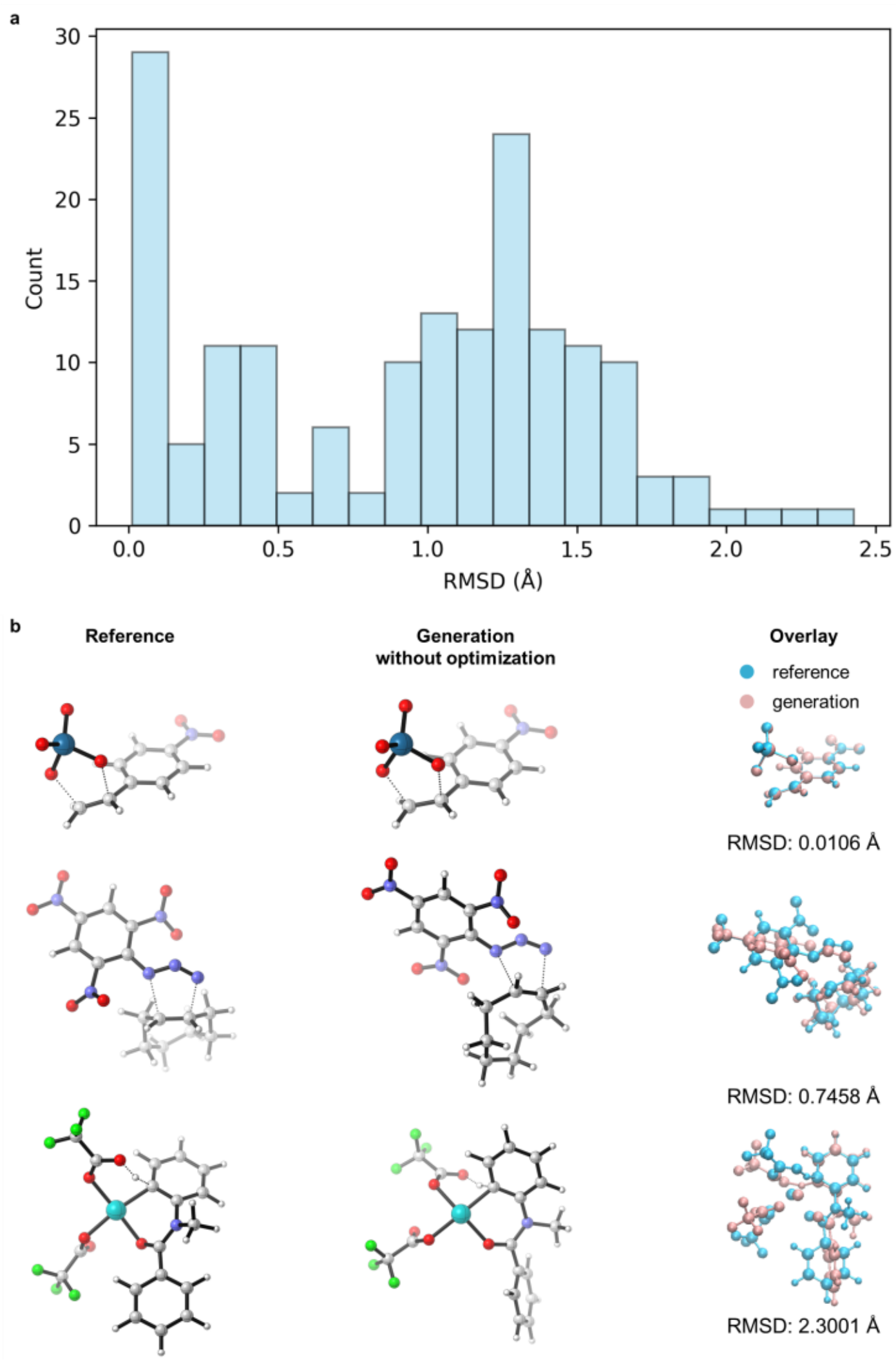

Supplementary Figure 9 | RMSD distribution and structural examples from the UniTS-Lib test set. a, Histogram of RMSD values for generated TS structures. **b,** Overlay of generated (pink) and reference (blue) structures for representative cases with low, medium, and high RMSD.

For the UniTS-Lib Formula-OOS test set (Supplementary Figure 9), the selected RMSD values are 0.0106 Å, 0.7458 Å, and 2.3001 Å. The overlaid structures are shown in Supplementary Figure 9b. The RMSD histogram (Supplementary Figure 9a) is based IRC-validated Formula-OOS test results.

All nine generated structures shown in these figures successfully converged to a reactive saddle point upon DFT optimization.

Suppl. Note 12. Correlation between structural deviation and energy barrier

We performed the following analyses using the three representative structures (low, medium, and high RMSD) selected from each dataset (Transition1x, [3+2] cycloaddition, and UniTS-Lib).

First, we compared the single-point energy differences between the generated structures and the reference geometries without any geometry optimization. Second, we uniformly optimized all structures (including the reference geometries, because the original references were not computed with a consistent method), performed IRC calculations, optimized the end-points along the IRC paths, and calculated the corresponding free energy barriers. Finally, we compared the barrier differences between the generated and reference structures. All calculations were carried out using the same level of theory (B3LYP-D3BJ/def2SVP). The results are presented in Figs. S10–12.

As shown in Figs. S10–12, for all three datasets, when the RMSD between the generated and reference structures is around 0.01 Å or lower, the single-point energy difference is small even without optimization (first row of each figure). Among these, UniTS-Lib, which contains the most complex molecules, shows the largest single-point energy difference within this group, yet it remains below 1 kcal/mol (Supplementary Figure 10, first row). When RMSD reaches medium or high levels, the single-point energy differences become substantial.

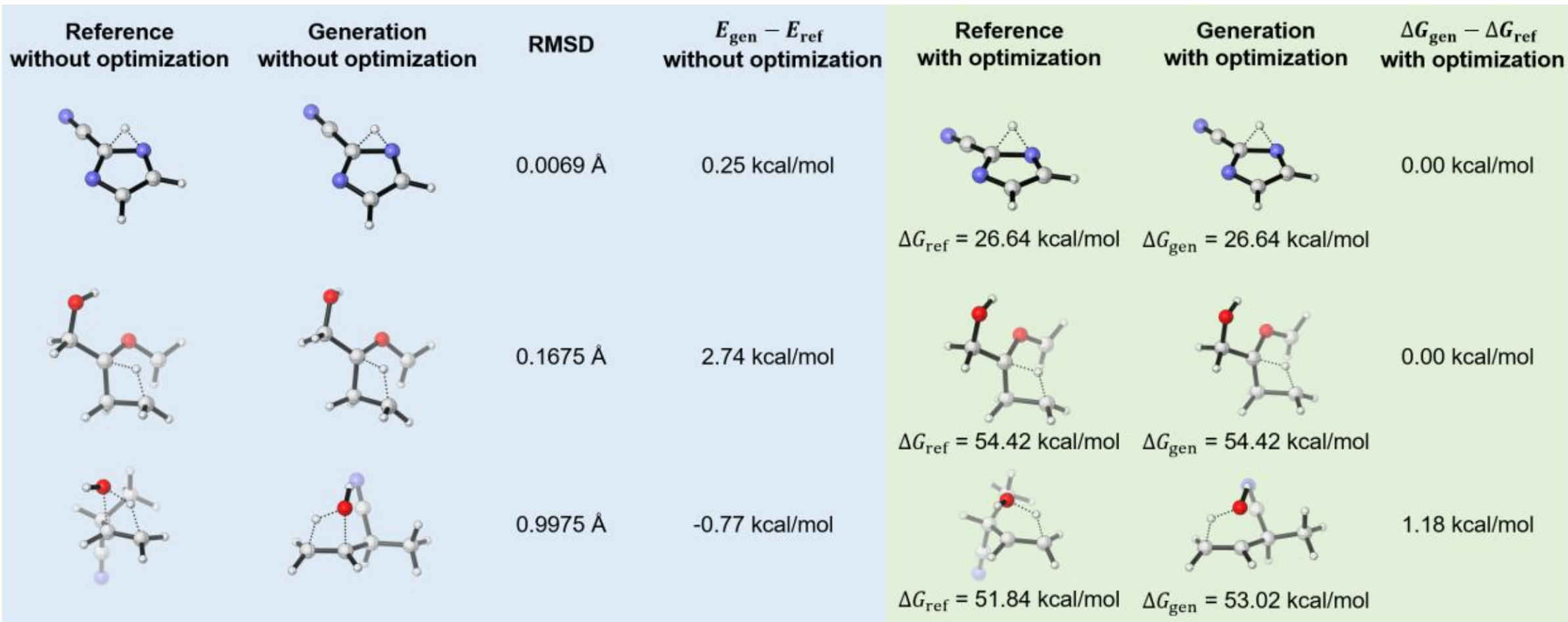

Supplementary Figure 10 | RMSD and barrier error of representative structures from Transition1x.

For the Transition1x dataset, the energy differences are relatively small due to the simpler molecular systems; the largest difference (2.74 kcal/mol) is observed for the medium-RMSD case, which corresponds to a conformational difference. After DFT optimization, the free energy barrier difference for the low-RMSD and the medium-RMSD (conformational difference) cases became negligible (0.00 kcal/mol, Supplementary Figure 10, first and second rows). However, for the high-RMSD case (configurational difference), a free energy barrier difference of 1.18 kcal/mol remained after optimization (Supplementary Figure 10, third row).

For the more complex [3+2] cycloaddition and UniTS-Lib datasets, the medium- and high-RMSD structures exhibit considerably larger single-point energy differences relative to the references (Figs. S11 and S12, second and third rows). This is mainly because, in these complex systems, small changes in bond lengths and angles related to bond-forming/-breaking events can lead to significant energy variations. Nevertheless, after DFT optimization, the barrier differences between the generated and reference structures are

substantially reduced.

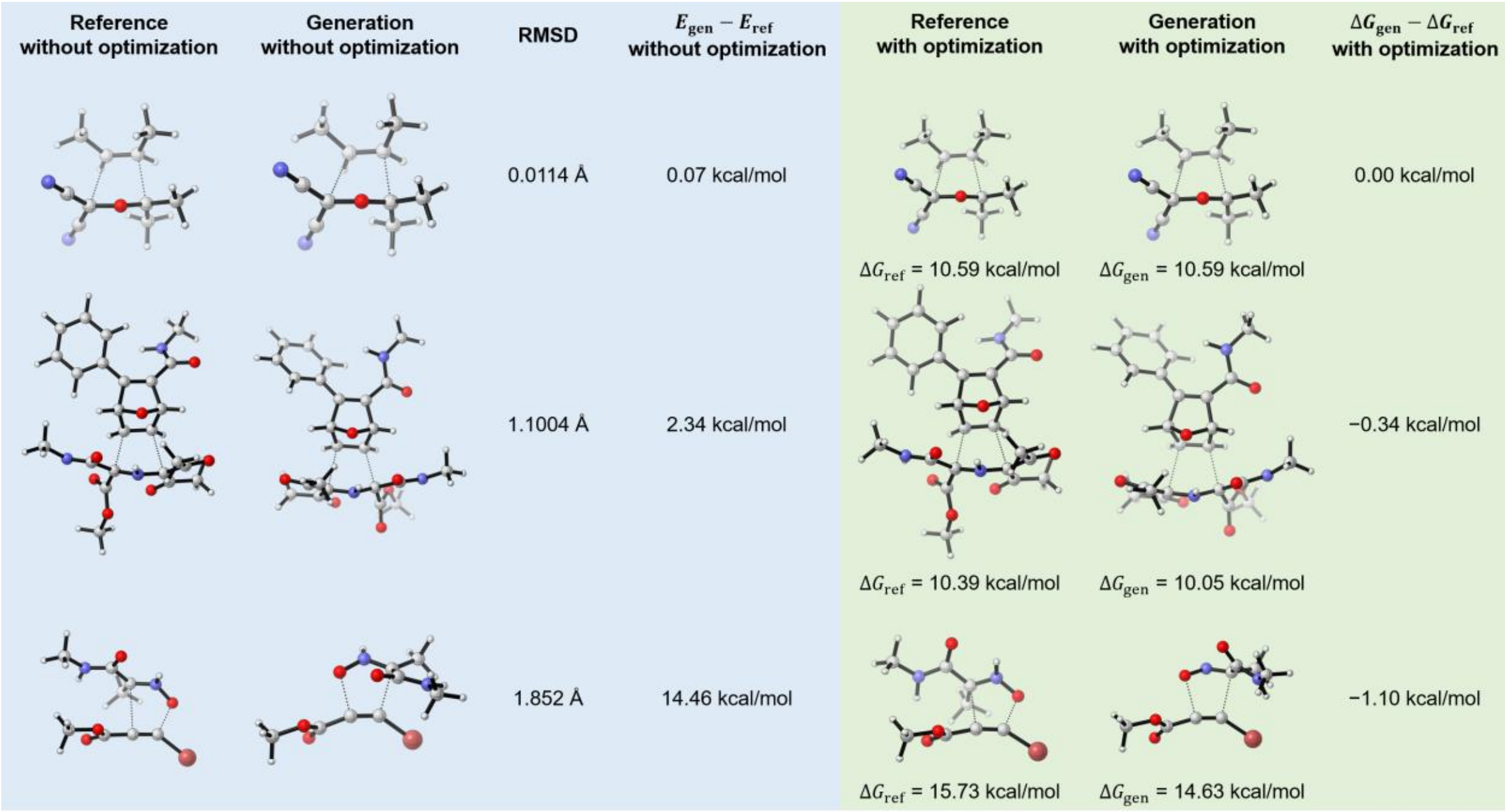

Supplementary Figure 11 | RMSD and barrier error of representative structures from [3+2]-cycloaddition dataset.

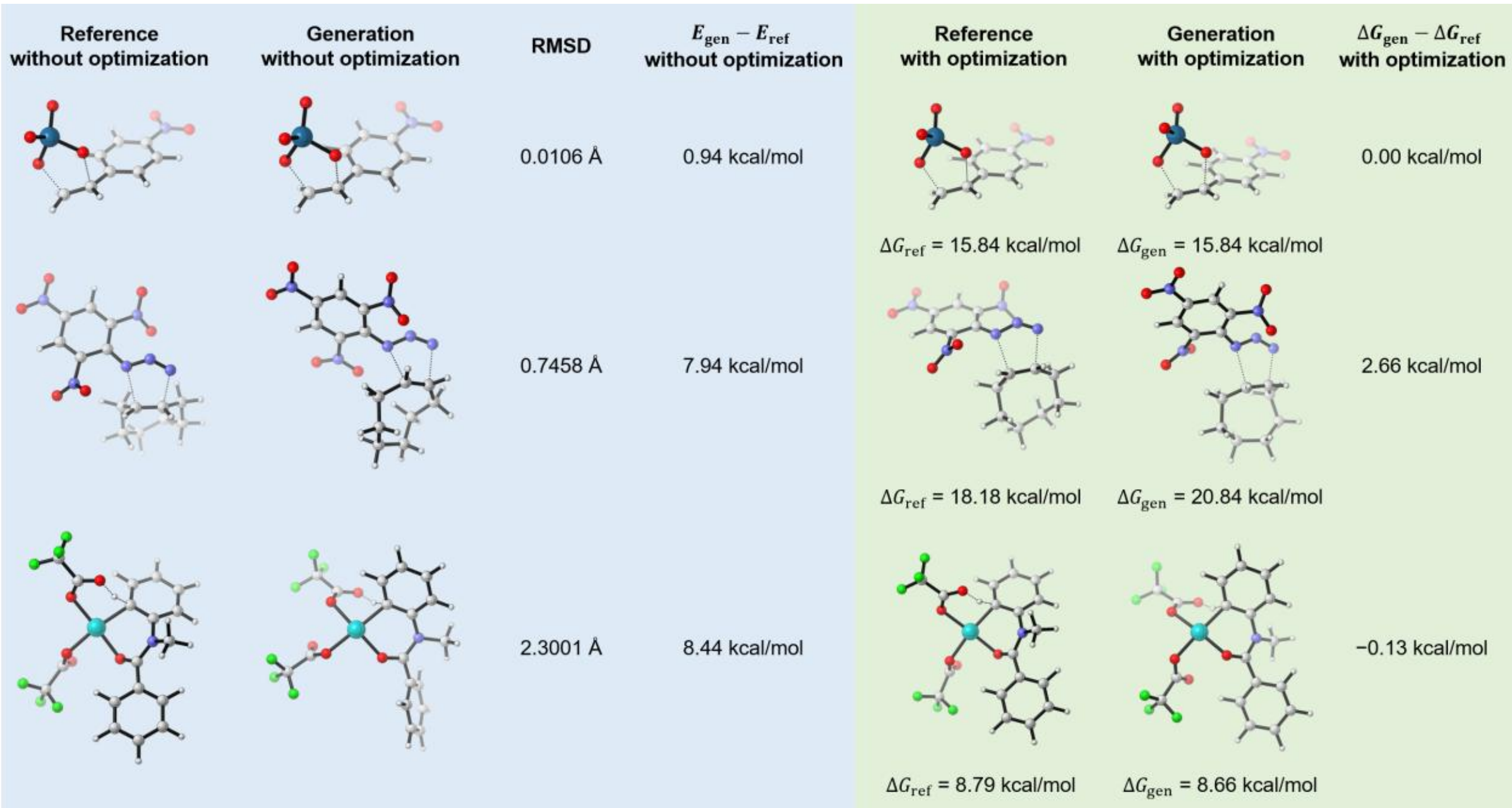

Supplementary Figure 12 | RMSD and barrier error of representative structures from UniTS-Lib.

Suppl. Note 13. Detailed evaluation of UniTS-Gen on the Formula-OOS test set

Formula-OOS test set comprises 403 TS structures whose elemental composition (formula) was entirely absent from the model's training data. The model achieved a RMSD of 1.15 Å and a low clash ratio of 1.5% on this OOS subset.

To assess the chemical utility of these generated structures, they were used as initial guesses for downstream DFT optimization. The success metrics are as follows:

1. Optimization success: 337 structures (83.6% of the total) successfully converged during DFT optimization.

2. First-order saddle point confirmation: Following frequency analysis, 277 of the optimized structures (68.7% of the total) were confirmed to possess one and only one imaginary frequency, indicating a true first-order saddle point on the potential energy surface.

3. Reaction relevance validation: After manual inspection to exclude non-reactive saddle points (e.g., those corresponding to simple group rotations or conformational changes unrelated to bond making/breaking), 169 structures (41.9% of the total) were confirmed as relevant TS structures associated with the chemical reaction, all of which passed IRC validation. The IRC results is available via https://github.com/licheng-xu-echo/UniTS.

Furthermore, we selected five previously failed cases from the Formula-OOS test set and performed multi-sampling: for each reaction, we generated 10 TS guesses and submitted all to DFT optimization. All five reactions successfully yielded the correct transition state, as confirmed by IRC validation (Supplementary Figure 13). This demonstrates that multi-sampling can substantially improve the success rate for challenging systems.

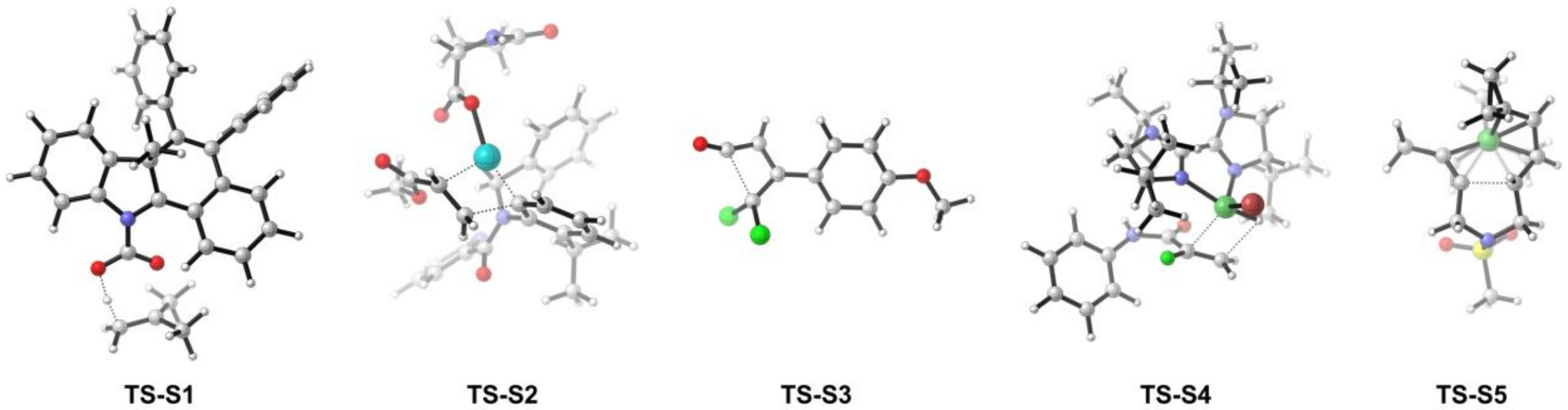

Supplementary Figure 13 | Optimized transition states for five previously failed Formula-OOS cases.

Supplementary Figure 14 displays a representative example from the Formula-OOS test set, showing both the initial TS structure generated by UniTS-Gen and the final, validated first-order saddle point structure obtained after DFT optimization using the generated structure as a starting point. Further examples and detailed data can be accessed through our public GitHub repository: https://github.com/licheng-xu-echo/UniTS.

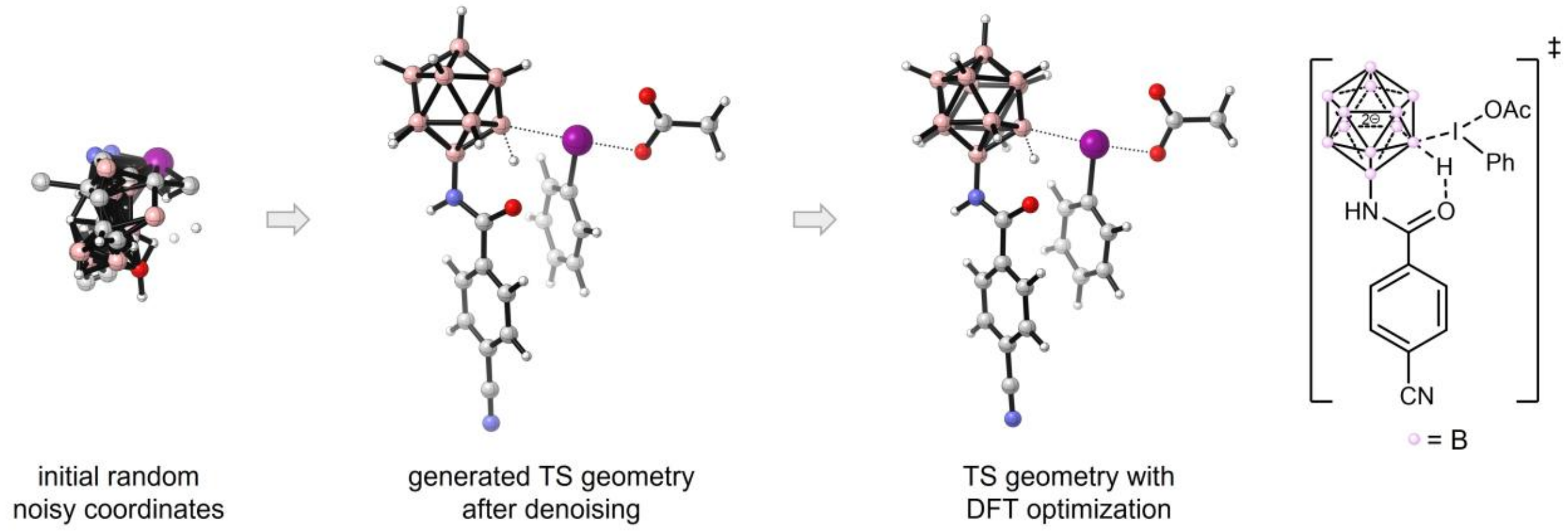

Supplementary Figure 14 | A showcase from Formula-OOS test set, illustrating UniTS-Gen denoising and subsequent DFT optimization.

Suppl. Note 14. Code and data availability

The processed UniTS-Lib dataset, as well as the code used for data collection, data preprocessing, model training, and TS generation, are accessible through https://github.com/licheng-xu-echo/UniTS.

Supplementary references

1. Frisch, M. J. *et al.* Gaussian 09 Revision D.01. (2016).

2. Lee, C., Yang, W. & Parr, R. G. Development of the Colle-Salvetti correlation-energy formula into a functional of the electron density. *Phys. Rev. B* **37**, 785–789 (1988).

3. Becke, A. D. Density-functional thermochemistry. III. The role of exact exchange. *J. Chem. Phys.* **98**, 5648–5652 (1993).

4. Grimme, S., Antony, J., Ehrlich, S. & Krieg, H. A consistent and accurate *ab initio* parametrization of density functional dispersion correction (DFT-D) for the 94 elements H-Pu. *J. Chem. Phys.* **132**, 154104 (2010).

5. Grimme, S., Ehrlich, S. & Goerigk, L. Effect of the damping function in dispersion corrected density functional theory. *J. Comput. Chem.* **32**, 1456–1465 (2011).

6. Schäfer, A., Horn, H. & Ahlrichs, R. Fully optimized contracted Gaussian basis sets for atoms Li to Kr. *J. Chem. Phys.* **97**, 2571–2577 (1992).

7. Stuyver, T., Jorner, K. & Coley, C. W. Reaction profiles for quantum chemistry-computed [3 + 2] cycloaddition reactions. *Sci. Data* **10**, 66 (2023).

8. Guo, X., Zhang, Y., Lai, X., Pang, Y. & Xue, X. C(sp^3)−F Bond Activation by Lewis Base-Boryl Radicals via Concerted Electron-Fluoride Transfer. *Angew. Chem. Int. Ed.* **64**, e202415715 (2025).

9. Wang, T. & Xue, X.-S. Ambimodal (2 + 1)/(4 + 1) Pseudopericyclic Transition States in Reactions between Dihalocarbenes and Olefins. *J. Am. Chem. Soc.* **148**, 8723–8735 (2026).

10. Singleton, D. A., Hang, C., Szymanski, M. J. & Greenwald, E. E. A New Form of Kinetic Isotope Effect.

Dynamic Effects on Isotopic Selectivity and Regioselectivity. *J. Am. Chem. Soc.* **125**, 1176–1177 (2003).

11. Legault, C. Y. CYLview, 1.0b. Université de Sherbrooke (2009).

12. Tang, M. & Hong, X. MolOP@v0.1.34.1. Zenodo https://doi.org/10.5281/ZENODO.15771599 (2025).

13. RDKIt. https://www.rdkit.org/.

14. Xu, L.-C., Tang, M.-J., An, J., Cao, F. & Qi, Y. A unified pre-trained deep learning framework for cross-task reaction performance prediction and synthesis planning. *Nat. Mach. Intell.* **7**, 1561–1571 (2025).

15. Liao, Y.-L., Wood, B. M., Das, A. & Smidt, T. E. EquiformerV2: Improved Equivariant Transformer for Scaling to Higher-Degree Representations. in *The Twelfth International Conference on Learning Representations, ICLR 2024, Vienna, Austria, May 7-11, 2024* (OpenReview.net, 2024).

16. Duan, C. *et al.* Optimal transport for generating transition states in chemical reactions. *Nat. Mach. Intell.* **7**, 615–626 (2025).

17. Kim, S., Woo, J. & Kim, W. Y. Diffusion-based generative AI for exploring transition states from 2D molecular graphs. *Nat. Commun.* **15**, 341 (2024).

18. Duan, C., Du, Y., Jia, H. & Kulik, H. J. Accurate transition state generation with an object-aware equivariant elementary reaction diffusion model. *Nat. Comput. Sci.* **3**, 1045–1055 (2023).
