# Supplementary material for: Automated transition state generation for mechanistic exploration in organic synthesis
Source: Nat Commun. 2026 Aug 27;17:10254. doi: 10.1038/s41467-026-77230-8 (PMC13616924; doi:10.1038/s41467-026-77230-8)
Supplement: Supplementary file 2 [file 41467_2026_77230_MOESM2_ESM.pdf]

nature portfolio

Corresponding author(s): Li-Cheng Xu, Fenglei Cao, and Yuan Qi

Last updated by author(s): Jul 3, 2026

Reporting Summary

Nature Portfolio wishes to improve the reproducibility of the work that we publish. This form provides structure for consistency and transparency in reporting. For further information on Nature Portfolio policies, see our Editorial Policies and the Editorial Policy Checklist.

Statistics

For all statistical analyses, confirm that the following items are present in the figure legend, table legend, main text, or Methods section.

n/a	Confirmed	
☒	☐	The exact sample size (n) for each experimental group/condition, given as a discrete number and unit of measurement
☒	☐	A statement on whether measurements were taken from distinct samples or whether the same sample was measured repeatedly
☒	☐	The statistical test(s) used AND whether they are one- or two-sided<br>*Only common tests should be described solely by name; describe more complex techniques in the Methods section.*
☒	☐	A description of all covariates tested
☒	☐	A description of any assumptions or corrections, such as tests of normality and adjustment for multiple comparisons
☒	☐	A full description of the statistical parameters including central tendency (e.g. means) or other basic estimates (e.g. regression coefficient) AND variation (e.g. standard deviation) or associated estimates of uncertainty (e.g. confidence intervals)
☒	☐	For null hypothesis testing, the test statistic (e.g. F, t, r) with confidence intervals, effect sizes, degrees of freedom and P value noted<br>*Give P values as exact values whenever suitable.*
☒	☐	For Bayesian analysis, information on the choice of priors and Markov chain Monte Carlo settings
☒	☐	For hierarchical and complex designs, identification of the appropriate level for tests and full reporting of outcomes
☒	☐	Estimates of effect sizes (e.g. Cohen's d, Pearson's r), indicating how they were calculated

Our web collection on statistics for biologists contains articles on many of the points above.

Software and code

Policy information about availability of computer code

Data collection	Transition state structures were collected from the Supplementary Information of published articles using PyMuPDF (fitz, v.1.26.7). Molecular structures were processed and standardized using RDKit (v.2025.03.5), MolOP (v.0.1.34.1), and Open Babel (openbabel-wheel, v.3.1.1.22). Geometry optimizations and electronic structure calculations were performed using Gaussian 09 Revision D.01.
Data analysis	Data analysis and model development were performed using Python 3.11 with PyTorch (v.2.4.0), PyTorch Geometric (v.2.6.1), e3nn (v.0.5.7), NumPy (v.1.26.4), SciPy (v.1.16.1), Matplotlib (v.3.10.6), Pymatgen (v.2025.6.14), and RDKit (v.2025.3.5). Custom analysis scripts used in this study are available through the GitHub repository archived in Zenodo, as described in the Methods section.

For manuscripts utilizing custom algorithms or software that are central to the research but not yet described in published literature, software must be made available to editors and reviewers. We strongly encourage code deposition in a community repository (e.g. GitHub). See the Nature Portfolio guidelines for submitting code & software for further information.

Data

Policy information about availability of data

All manuscripts must include a data availability statement. This statement should provide the following information, where applicable:

- Accession codes, unique identifiers, or web links for publicly available datasets
- A description of any restrictions on data availability
- For clinical datasets or third party data, please ensure that the statement adheres to our policy

The UniTS-Lib dataset, the preprocessed Transition1x dataset, and the [3+2]-cycloaddition dataset used in this study have been deposited in the Figshare: https://doi.org/10.6084/m9.figshare.31338676. The IRC results generated in this study have been deposited in the GitHub: https://github.com/licheng-xu-echo/UniTS. All data used and generated in this study are publicly available. Source data are provided with this paper.

Research involving human participants, their data, or biological material

Policy information about studies with human participants or human data. See also policy information about sex, gender (identity/presentation), and sexual orientation and race, ethnicity and racism.

Reporting on sex and gender	N/A
Reporting on race, ethnicity, or other socially relevant groupings	N/A
Population characteristics	N/A
Recruitment	N/A
Ethics oversight	N/A

Note that full information on the approval of the study protocol must also be provided in the manuscript.

Field-specific reporting

Please select the one below that is the best fit for your research. If you are not sure, read the appropriate sections before making your selection.

☒ Life sciences ☐ Behavioural & social sciences ☐ Ecological, evolutionary & environmental sciences

For a reference copy of the document with all sections, see nature.com/documents/nr-reporting-summary-flat.pdf

Life sciences study design

All studies must disclose on these points even when the disclosure is negative.

Sample size	Sample sizes of UniTS-Lib were based on calculation and quality-filtered TS structures (4,391 in total), which is comparable to existing benchmarks and sufficient for training, validation, and testing.
Data exclusions	No data were excluded from the analyses.
Replication	To ensure the reproducibility of our experimental findings, we took the following measures: (1) we provided detailed descriptions of the data splitting procedure; (2) we made available the model weights for all models used in testing.
Randomization	For the Transition1x dataset, we adopted the same data split as reported in previous studies. For all other random splits, we provided the random seeds used for data partitioning.
Blinding	Blinding was not relevant to this study, as all data were derived from published literature or generated by deterministic computational methods without any human assignment to groups.

Reporting for specific materials, systems and methods

We require information from authors about some types of materials, experimental systems and methods used in many studies. Here, indicate whether each material, system or method listed is relevant to your study. If you are not sure if a list item applies to your research, read the appropriate section before selecting a response.

Materials & experimental systems

n/a	Involved in the study
☒	☐ Antibodies
☒	☐ Eukaryotic cell lines
☒	☐ Palaeontology and archaeology
☒	☐ Animals and other organisms
☒	☐ Clinical data
☒	☐ Dual use research of concern
☒	☐ Plants

Methods

n/a	Involved in the study
☒	☐ ChIP-seq
☒	☐ Flow cytometry
☒	☐ MRI-based neuroimaging

Plants

Seed stocks: N/A

Novel plant genotypes: N/A

Authentication: N/A
