# Supplementary material for: Automated transition state generation for mechanistic exploration in organic synthesis
Source: Nat Commun. 2026 Aug 27;17:10254. doi: 10.1038/s41467-026-77230-8 (PMC13616924; doi:10.1038/s41467-026-77230-8)
Supplement: Supplementary file 3 [file 41467_2026_77230_MOESM3_ESM.pdf]

nature portfolio

Peer Review File

Automated transition state generation for mechanistic exploration in organic synthesis

Corresponding Author: Dr Li-Cheng Xu

This file contains all reviewer reports in order by version, followed by all author rebuttals in order by version.

Version 0:

Reviewer comments:

Reviewer #1

(Remarks to the Author)
This manuscript addresses an important problem in chemical reaction and the dataset curation effort is timely. However, in its current form, the paper significantly overclaims what has been achieved. The work demonstrates a conditional TS-guess generator under strong assumptions, not a universal framework for automated transition-state discovery. I do not think the present evidence supports publication in Nature Communications at its current form.
----
major concerns
1. The central claims are overstated. The repeated use of “universal,” “general-purpose,” and “automated mechanistic discovery” is not justified by the results. On the authors’ own challenging Formula-OOS setting, only 68.7% of generated structures converge to a first-order saddle point, and only 41.9% are manually verified as the correct TS. That is promising, but far from universal or broadly reliable.
2. The method is not “truly automated”. The model does not infer the reaction center. It requires reactive-site indices as an essential conditioning input. In realistic mechanistic exploration, specifying the correct reactive site is often one of the hardest parts of the problem. The manuscript should therefore not present this as automated TS discovery in any strong sense.
3. The dataset is too sparse to support the “universal” framing. A library of 4,391 TS structures spanning 42 elements, multiple reaction classes, charged species, radicals, and systems up to 231 atoms is broad, but also extremely sparse once partitioned into meaningful chemical regimes. The likely per-regime sample count is small. The observed degradation on Element-OOS and Maximum-OOS is consistent with exactly this limitation. Along the line, Savoie et al. has achieved a much extended effort along the similar line (https://chemrxiv.org/doi/full/10.26434/chemrxiv-2025-ccgfs).
4. TS validation appears insufficiently rigorous. I do not see clear evidence of systematic IRC verification. The manuscript describes frequency analysis and graph matching, and later manual filtering of “ineffective transition states” such as simple group rotations. That is not enough to establish that the optimized structures connect the intended minima, especially for flexible or organometallic systems. This is a major weakness for a paper whose main claim rests on TS correctness.
5. The dataset should not be described so confidently as “high-fidelity.” All structures are standardized at B3LYP-D3(BJ)/def2-SVP. For a library spanning radicals, ions, transition metals, and heavy elements up to Au, this is a practical uniform protocol, not an obvious high-fidelity reference standard. Without IRC verification and without deeper discussion of method limitations across such diverse chemistry, the language is too strong.
6. The train/test design does not convincingly establish generalization. The main split is random on a literature-derived dataset assembled from 346 sources. That leaves substantial room for leakage of scaffolds, catalyst classes, ligand motifs, or recurring mechanistic patterns. The OOS tests are useful, but still not stringent enough to justify strong extrapolation claims. A paper-level, catalyst-family, or template-level split is needed.
7. The evidence for reactive-site control is anecdotal. The manuscript includes one selectivity example showing that the same reactant graph with different reactive-site indices can lead to different downstream TS structures. But breaking C-H and C-F share similar chemistry and structures and should be considered as esay examples. That is a useful illustration, but not a systematic validation of the conditioning mechanism. Given how central this input is to the method, the evidence is too thin.
8. RMSD is overused and underqualified. The manuscript reports RMSD across datasets with very different size distributions and chemical complexity. UniTS-Lib has median sizes around 50 atoms and maxima up to 231, whereas Transition1x contains much smaller systems. Absolute RMSD values are therefore not directly comparable across datasets. The manuscript itself shows that RMSD can mis-rank chemically nonsensical collapsed structures as better than chemically

plausible but configurationally different ones. This makes the cross-dataset presentation misleading unless heavily qualified. Adding more examples in each dataset to show what the numbers mean structurally (by overlapping generated and reference structures) and kinetically (by computing the barrier error) are much needed.
9. The application section is too limited for the claims being made. Two case studies are not enough to support broad claims about selectivity analysis or mechanistic discovery. The claim that the model found a lower-barrier structure than the literature in the [3+2] example is especially overinterpreted; this could reflect many factors besides superior generative capability.
---
minor comments
The title and abstract should be toned down substantially. “Universal framework” is not supported by the results.
The manuscript should clearly distinguish “first-order saddle point” from “correct transition state for the intended pathway.” At present these ideas are blurred.

(Remarks on code availability)
I do not have enough time to dig into the codes this time and I think there are major issues in the work that need to be addressed first prior to that.

Reviewer #2

(Remarks to the Author)
In this manuscript, Xu and co-workers present a general framework for automated transition-state generation that combines a curated dataset (UniTS-Lib) with a diffusion-based generative model (UniTS-Gen). The approach enables the direct generation of three-dimensional TS structures from two-dimensional reactant representations and addresses a longstanding challenge in computational chemistry: the efficient construction of reliable TS initial guesses for mechanistic exploration. The authors compile a diverse dataset of 4,391 high-quality TS structures spanning a broad range of elements and reaction types, and develop a higher-degree equivariant diffusion model to generate TS geometries. The benchmarking results demonstrate strong accuracy and promising generalization capability, including transferability to previously unseen reaction classes. The framework is further shown to accelerate TS discovery by providing high-quality initial structures for subsequent optimization.

Overall, this work addresses an important and timely problem at the interface of computational chemistry and machine learning. The integration of dataset construction, methodological innovation, and application to mechanistic studies represents a valuable contribution. The manuscript is clearly written and technically sound. I recommend publication in Nature Communications after minor revision:

1) While prior TS generation methods are discussed, a more explicit comparison with representative state-of-the-art approaches (in terms of accuracy, robustness, or computational efficiency) would help better contextualize the advantages of the present method.
2) It would be interesting to explore whether the proposed TS generation approach can be extended to more complex scenarios, such as ambimodal transition states (e.g., Ess, Wheeler, Houk, and co-workers, Angew. Chem. Int. Ed. 2008, 47, 7592–7601). Even a brief discussion of the potential applicability or limitations in this context would further highlight the scope of the method.).
3) A brief clarification of technical terminology (e.g., “higher-degree equivariant network”) would improve accessibility for a broader readership.

(Remarks on code availability)

Reviewer #3

(Remarks to the Author)
This is valuable work on the field of generative AI for transition states, remarkably supporting metals and a wide diversity of complex reactions. The TS library will be a useful resource for the community. The topology-informed 2D graph to 3D geometry transform is appealing and appears to be a superior alternative to previous diffusion models. Adding further value, the study goes beyond conventional metrics and tests the generated TS geometry guesses via actual DFT saddle point optimizations, yielding a success rate similar to what an experienced computational chemist would achieve.

Minor revisions:

Fig. 2a: The chords color legend can be better - Nearly only two colors appear to be relevant in the plot.

Fig. 2 analysis: If possible, give statistics over reaction types.

Fig 3: "defuse" should be "diffuse"?

"multi-configurational" may cause confusion (multi-reference in electronic structure theory) - try to find an alternative term.

The Methods section is too succinct. Give more detail. In the Computational Details, state (if true) that no IRC calculations were carried out. In the UniTS-Gen Architecture, give some minimal math expressing the architecture of the model (of particular interest is the 2D-conditioning of the 3D geometry generation) and the loss function used to train it. TASFs: In the equation, what is the difference between the $embeddingi$0 terms on both sides of the equals sign? Revise the text discussing the TASFs making a bit longer if possible and also clearer.

Congratulations for the impressive work done.

(Remarks on code availability)

Version 1:

Reviewer comments:

Reviewer #1

(Remarks to the Author)
The authors have done a great job in this revision. They tone down many statements, making this high-quality manuscript more rigorous. I also like the new results on quasi-classical dynamics and overlapping visualization examples for different dataset, which makes it much clear. My only remaining comment is about energy barriers for different datasets. I understand the authors' arguement regarding to conformations, but the same arguement holds for RMSD, no? I would encourage the authors to compute energy barriers in some ways, so that the audience can have a idea about how barrier errors changes among different datasets and molecule sizes.

(Remarks on code availability)

Reviewer #2

(Remarks to the Author)
The authors have thoroughly and convincingly addressed all of this reviewer's concerns. In particular, they demonstrate that their model can successfully handle ambimodal transition-state scenarios, which are inherently complex, previously unexplored, and highly sensitive to subtle bond-length variations within the reactive region. This represents a particularly compelling validation of the robustness and generality of the approach.

Importantly, the study highlights the potential of the model to address challenging mechanistic problems that are difficult to analyze using conventional intuition alone, making the work highly relevant to both synthetic and mechanistic chemists. Overall, this work demonstrates substantial conceptual and methodological significance, and I strongly support its publication in Nature Communications.

(Remarks on code availability)

Version 2:

Reviewer comments:

Reviewer #1

(Remarks to the Author)
I thank the authors for adding the barrier error examples, which give us an estimation about the relationship between RMSD and barrier errors. I recommend its publication now.

(Remarks on code availability)

Open Access This Peer Review File is licensed under a Creative Commons Attribution 4.0 International License, which permits use, sharing, adaptation, distribution and reproduction in any medium or format, as long as you give appropriate credit to the original author(s) and the source, provide a link to the Creative Commons license, and indicate if changes were made.
In cases where reviewers are anonymous, credit should be given to 'Anonymous Referee' and the source.
The images or other third party material in this Peer Review File are included in the article's Creative Commons license, unless indicated otherwise in a credit line to the material. If material is not included in the article's Creative Commons license and your intended use is not permitted by statutory regulation or exceeds the permitted use, you will need to obtain permission directly from the copyright holder.
To view a copy of this license, visit https://creativecommons.org/licenses/by/4.0/

Response to Referee #1

Comment: This manuscript addresses an important problem in chemical reaction and the dataset curation effort is timely. However, in its current form, the paper significantly overclaims what has been achieved. The work demonstrates a conditional TS-guess generator under strong assumptions, not a universal framework for automated transition-state discovery. I do not think the present evidence supports publication in Nature Communications at its current form.

Response: We thank the reviewer for acknowledging the importance of our work and for the rigorous assessment of the claims and language in our manuscript. Following the reviewer's guidance, we have added new case studies to further demonstrate the practical value of UniTS-Gen in automated mechanistic exploration. Regarding the term "universal", we would like to clarify that it was intended to convey that our framework represents a promising universal framework and serves as a key component toward automated transition state discovery, as evidenced by its ability to generate transition states across a wide range of element types, molecular sizes, and reaction types, rather than claiming exhaustive coverage of all possible TS structures. Nevertheless, we agree that the original wording could be overclaimed. We have therefore carefully toned down the language throughout the manuscript and more precisely defined the scope of our method. We believe these changes bring the claims more in line with the evidence presented and better meet the high standards of *Nature Communications*.

----

major concerns

Comment: 1. The central claims are overstated. The repeated use of "universal," "general-purpose," and "automated mechanistic discovery" is not justified by the results. On the authors' own challenging Formula-OOS setting, only 68.7% of generated structures converge to a first-order saddle point, and only 41.9% are manually verified as the correct TS. That is promising, but far from universal or broadly reliable.

Response: We thank the reviewer for the careful assessment of our language. Regarding the terms "universal" and "general-purpose", we would like to clarify that they were intended to reflect UniTS-Gen's ability to generate accurate TS initial guesses across broad element types, a wide range of molecular sizes, and diverse reaction types (including complex systems such as an icosahedral borane cluster), as demonstrated by our results. In the revised manuscript, we have therefore described these capabilities as a promising universal/general-purpose framework, rather than making absolute claims. Furthermore, following the reviewer's later suggestion regarding the title, we have changed "universal" to "unified", a term that more accurately conveys that our contribution provides an integrated pipeline combining TS data curation and generative model development. We have also carefully revised other related terms and statements throughout the manuscript to ensure the overall claims align with the demonstrated capabilities.

Regarding the reviewer's concern about the 41.9% manually verified success rate on the Formula-OOS test (with a single generated sample per reaction), we would like to offer the following perspective. First, as noted in the manuscript, the construction of UniTS-Lib itself started from over 10,000 initial TS structures extracted from literature, yet after DFT optimization with a

consistent protocol, only 4,391 (roughly 40%) were confirmed as transition states, despite all being derived from published, expert-curated TS geometries. With this in mind, we feel that a single-shot success rate of ~40% from a purely generative model without manual intervention compares reasonably well with the success rate of expert-guided TS optimization, given the inherent difficulty of the task.

Second, TS optimization is known to be sensitive to initial geometry. For complex systems (e.g., iron-porphyrin or icosahedral borane), manually constructing a reasonable TS guess can be challenging and may require multiple attempts. Meanwhile, UniTS-Gen can generate multiple diverse guesses at modest additional cost. To further evaluate reliability under multi-sampling, we selected 5 previously failed cases from the Formula-OOS set, generated 10 samples for each, and submitted all to DFT optimization. Encouragingly, all 5 reactions successfully yielded the correct transition state, as confirmed by IRC validation. This suggests that when users are allowed to sample a small number of candidates, the effective success rate can be substantially improved for a broad range of challenging systems. We have added these results and discussions to the revised manuscript and Supplementary Information.

Comment: 2. The method is not "truly automated". The model does not infer the reaction center. It requires reactive-site indices as an essential conditioning input. In realistic mechanistic exploration, specifying the correct reactive site is often one of the hardest parts of the problem. The manuscript should therefore not present this as automated TS discovery in any strong sense.

Response: We thank the reviewer for this thoughtful comment. We agree that requiring reactive-site indices means the method is not fully automated in the strict sense. Accordingly, we have revised the manuscript to tone down claims of full automation. Instead, we position UniTS-Gen as a method designed to address the most labor-intensive bottleneck in TS discovery: the manual trial-and-error construction of reasonable 3D initial guesses. It serves as an essential component of, rather than a complete solution to, automated mechanistic discovery.

Regarding the identification of reactive sites, in our view this is a separate subproblem that can be addressed by different strategies depending on the application scenario. For routine mechanistic studies (e.g., investigating a specific transformation where the chemist already has reasonable hypotheses about bond forming/breaking), specifying the reactive atoms is straightforward and does not undermine the utility of our method. For large-scale, end-to-end automated reaction network exploration, reactive sites can be predicted by heuristic rules, a dedicated machine learning model, brute-force enumeration of all possible site combinations, or rule-based product enumeration frameworks such as YARP (*Nat. Comput. Sci.* **1**, 479–490 (2021)). Our framework is designed to be compatible with such upstream modules, which we have clarified in the revised manuscript. We hope this clarification better reflects the scope and positioning of our work.

Comment: 3. The dataset is too sparse to support the "universal" framing. A library of 4,391 TS structures spanning 42 elements, multiple reaction classes, charged species, radicals, and systems up to 231 atoms is broad, but also extremely sparse once partitioned into meaningful chemical regimes. The likely per-regime sample count is small. The observed degradation on Element-OOS

and Maximum-OOS is consistent with exactly this limitation. Along the line, Savoie et al. has achieved a much extended effort along the similar line (https://chemrxiv.org/doi/full/10.26434/chemrxiv-2025-ccgfs).

Response: We thank the reviewer for this insightful comment regarding the dataset. We agree that the current UniTS-Lib is sparse given the chemical space it aims to cover. However, we would like to highlight that this sparsity makes the strong performance of UniTS-Gen (HiEGNN) on the Formula-OOS test all the more notable. It demonstrates that a higher-degree equivariant architecture can learn meaningful patterns and generalize to unseen elemental compositions even from relatively limited data. In contrast, the standard EGNN kernel failed to produce chemically valid structures on the same dataset, underscoring that the architectural design is a key factor in achieving generalization under data sparsity. Thus, the "universal" qualification is justified with respect to the model architecture and its ability to handle diverse reaction systems, rather than implying exhaustive coverage of all chemical regimes.

We fully agree that the performance on Element-OOS and Maximum-OOS indicates room for improvement, which we view as a data limitation rather than a fundamental flaw of the architecture. As the reviewer suggests, expanding the training set is a clear path forward. The data curation workflow developed in this work can be readily applied to scale up UniTS-Lib. We are also grateful for the reviewer's pointer to the work by Savoie et al. We were aware of this independent effort, which focuses on transition metal catalysis and provides a valuable dataset of TS structures extracted from the literature. We note that the geometries in that work were optimized at the semi-empirical GFN2-xTB level. We believe this dataset represents an excellent training resource for TS generation targeting transition metal catalysis, and also serves as a promising starting point for constructing DFT-level TS datasets. In the future, we plan to leverage this dataset to further develop and train our model, with the goal of moving closer to a truly "universal" TS generation model. We have added these discussions to the revised manuscript. In addition, we have also acknowledged the current limitations of our model in the Discussion section of the revised manuscript.

Comment: 4. TS validation appears insufficiently rigorous. I do not see clear evidence of systematic IRC verification. The manuscript describes frequency analysis and graph matching, and later manual filtering of "ineffective transition states" such as simple group rotations. That is not enough to establish that the optimized structures connect the intended minima, especially for flexible or organometallic systems. This is a major weakness for a paper whose main claim rests on TS correctness.

Response: We thank the reviewer for this important suggestion regarding rigorous TS validation. Following the reviewer's advice, we have performed IRC calculations for all 169 TS structures in the Formula-OOS test set that had passed frequency analysis, graph matching, and manual filtering. All 169 structures passed IRC validation: for each of them, we verified that the IRC path connects two minima corresponding to bond-forming/-breaking events (rather than simple conformational changes). These IRC results have been included in the revised Supplementary Information and are available on GitHub.

Comment: 5. The dataset should not be described so confidently as “high-fidelity.” All structures are standardized at B3LYP-D3(BJ)/def2-SVP. For a library spanning radicals, ions, transition metals, and heavy elements up to Au, this is a practical uniform protocol, not an obvious high-fidelity reference standard. Without IRC verification and without deeper discussion of method limitations across such diverse chemistry, the language is too strong.

Response: We thank the reviewer for this constructive suggestion. We agree that “high-fidelity” is too strong given the single uniform DFT protocol used. We have replaced the term with either “consistently optimized” or “high-quality” throughout the manuscript to better reflect the standardized nature of our computational workflow.

Comment: 6. The train/test design does not convincingly establish generalization. The main split is random on a literature-derived dataset assembled from 346 sources. That leaves substantial room for leakage of scaffolds, catalyst classes, ligand motifs, or recurring mechanistic patterns. The OOS tests are useful, but still not stringent enough to justify strong extrapolation claims. A paper-level, catalyst-family, or template-level split is needed.

Response: We thank the reviewer for acknowledging our OOS tests and for the rigorous scrutiny of potential data leakage and generalization capability. To further address this concern and also respond to Reviewer 2's comment regarding applicability to ambimodal TS, we identified a recently published study (*J. Am. Chem. Soc.* **148**, 8723–8735 (2026), a paper-level OOS test) and took one transition state from this work that is not included in UniTS-Lib – specifically, one with an elemental composition unseen in UniTS-Lib (i.e., a Formula-OOS case). Using UniTS-Gen, we generated an initial TS guess for this system, which successfully converged to a correct transition state upon DFT optimization and passed IRC validation. Furthermore, quasi-classical molecular dynamics simulations based on this TS successfully reproduced the bifurcation behavior, yielding both [4+1] cycloaddition products (via C1–C5 and O4–C5 bond formation) and [2+1] cycloaddition products (via C1–C5 and C2–C5 bond formation), as shown in Figure 1. This demonstrates that our model can generate accurate initial TS geometries for ambimodal systems, where precise bond distances in the reactive region are critical. This external validation further demonstrates the generalization capability of our model and points to potential applications beyond conventional mechanistic analysis. We have included these results in the revised manuscript.

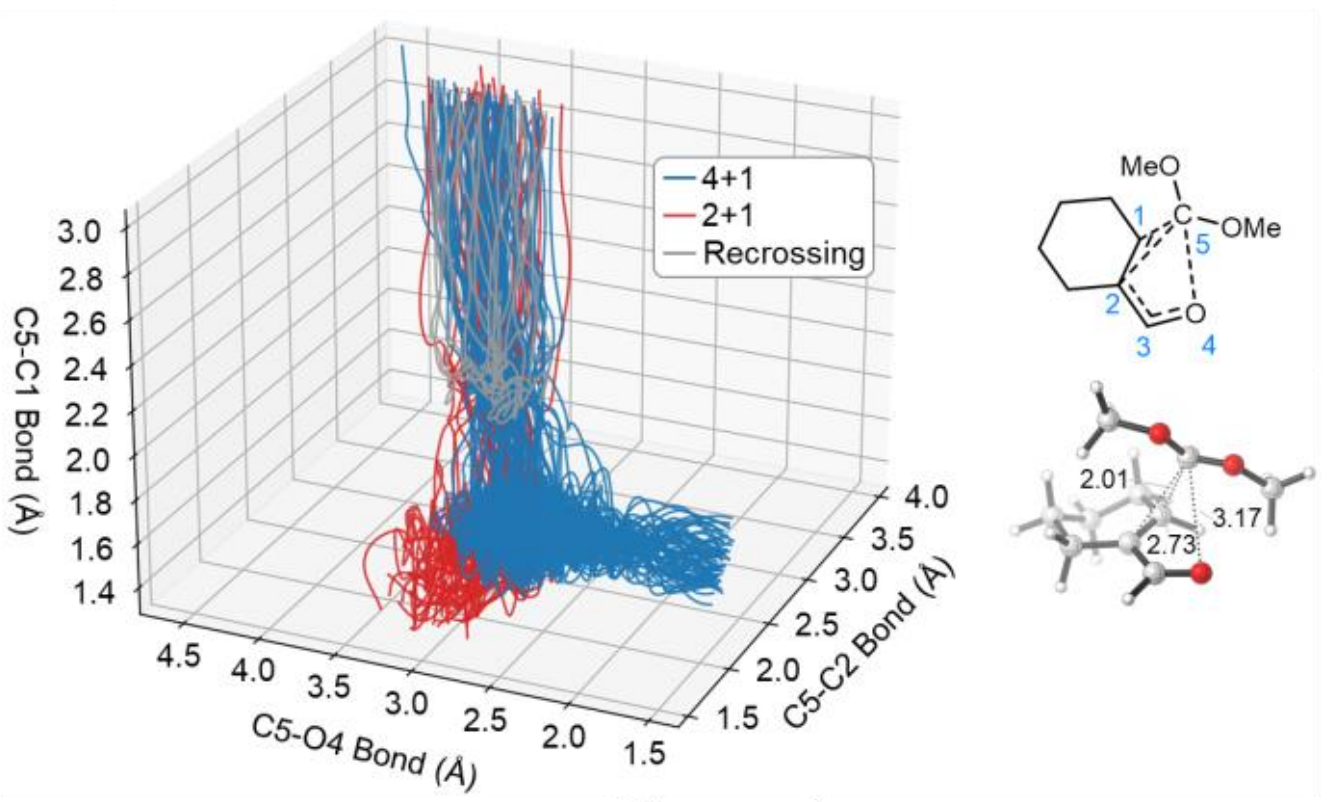

Fig. 1 Overlaid 3D trajectories from quasi-classical molecular dynamics simulations of the ambimodal transition state.

Comment: 7. The evidence for reactive-site control is anecdotal. The manuscript includes one selectivity example showing that the same reactant graph with different reactive-site indices can lead to different downstream TS structures. But breaking C-H and C-F share similar chemistry and structures and should be considered as esay examples. That is a useful illustration, but not a systematic validation of the conditioning mechanism. Given how central this input is to the method, the evidence is too thin.

Response: We thank the reviewer for this valuable suggestion. We would like to point out that generating different TS guesses from the same molecular graph by only changing the reactive site indices is inherently challenging, because the more similar the sites are, the more difficult it becomes for the conditional context to induce distinct transition states. In the C–H versus C–F example shown in Fig. 5a, we generated 10 independent samples for each site condition, and all 10 faithfully followed the conditioning to produce the expected TS geometries.

To further probe the limitations of our conditioning mechanism, we tested the ambimodal TS case mentioned in our previous response. Here, we specified the nucleophilic dimethoxycarbene carbon (C5) and the alkene double-bond carbon (C1) as the reactive sites. Out of 10 generated samples, only 7 yielded the intended TS initial guess; the remaining 3 instead generated a TS involving bond formation/breaking between the carbene carbon (C5) and the carbonyl carbon (C3) of the alkene substrate. This result indicates that when the reactive sites are highly similar in terms of their 2D molecular graph environment or chemical environment, and the system is unexplored, the conditioning mechanism may become less reliable. Nevertheless, our model still generated the expected TS guess in a substantial proportion (7 out of 10) of the sampling trials. We have added a discussion of these limitations in the revised manuscript.

Comment: 8. RMSD is overused and underqualified. The manuscript reports RMSD across datasets with very different size distributions and chemical complexity. UniTS-Lib has median sizes around 50 atoms and maxima up to 231, whereas Transition1x contains much smaller systems. Absolute RMSD values are therefore not directly comparable across datasets. The manuscript itself shows that RMSD can mis-rank chemically nonsensical collapsed structures as better than chemically plausible but configurationally different ones. This makes the cross-dataset presentation misleading unless heavily qualified. Adding more examples in each dataset to show what the numbers mean structurally (by overlapping generated and reference structures) and kinetically (by computing the barrier error) are much needed.

Response: We fully agree with the reviewer that RMSD is not an ideal metric, particularly for systems with large atom counts and for generative models capable of producing multiple conformers and configurations, as already discussed in our original manuscript. We continued to report RMSD mainly because it remains a commonly used metric in the field. We also acknowledge that cross-dataset RMSD comparisons are problematic due to the vastly different molecular sizes in the three datasets. In the revised manuscript, we have explicitly cautioned against such direct comparisons.

We greatly appreciate the reviewer's suggestion to provide concrete examples that illustrate what these RMSD numbers mean structurally. To this end, we selected from each of the three datasets three generated structures (single sample per reaction) representing low, medium, and high RMSD relative to the reference. These are presented in Figure 2–4 In each figure, panel (a) shows the

RMSD distribution of generated structures on the test set, and panel (b) shows the overlapping generated and reference structures for the three RMSD levels, allowing a visual appreciation of how RMSD correlates with structural differences.

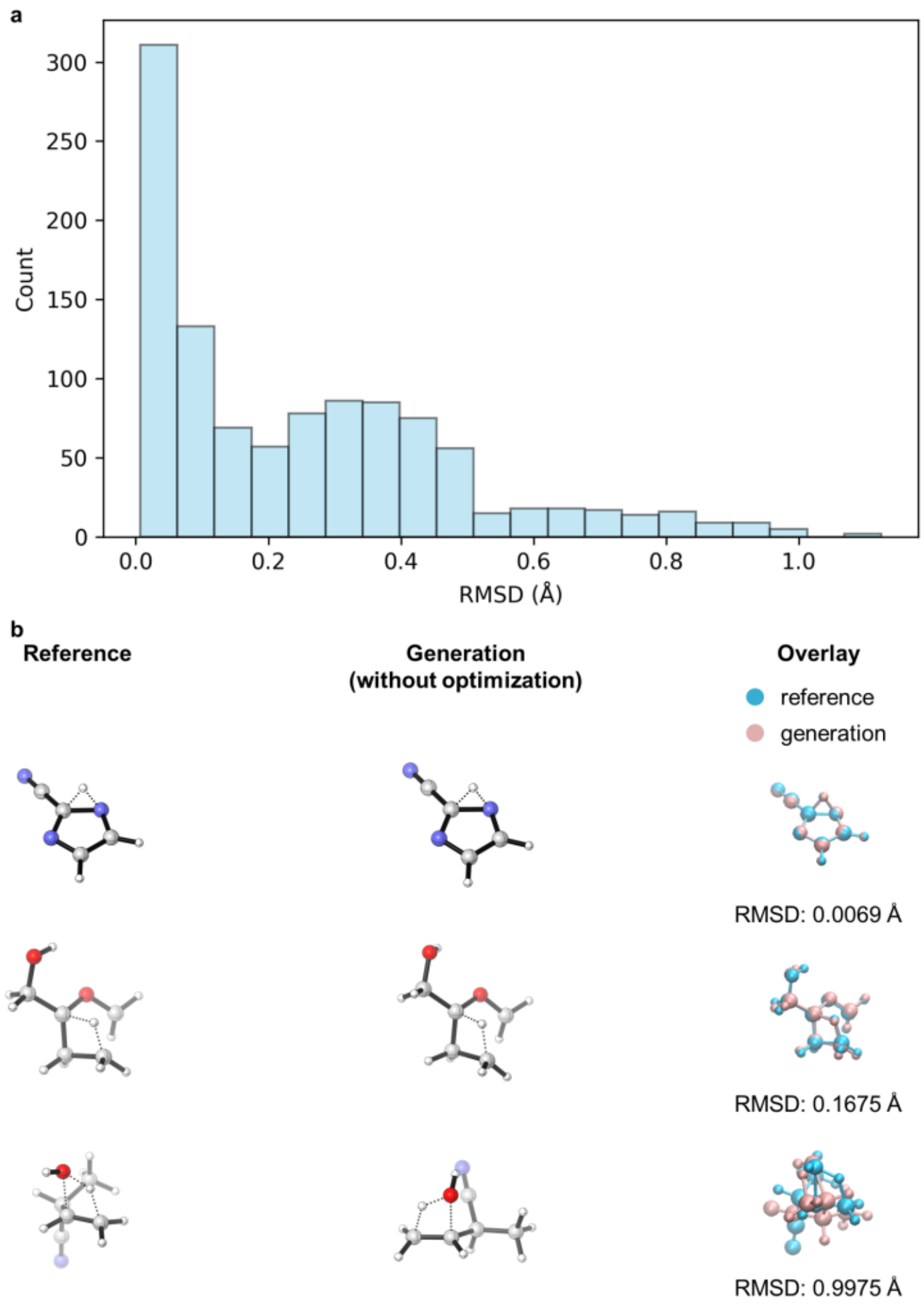

Fig. 2 RMSD distribution and structural examples from the Transition1x test set. a, Histogram of RMSD values for generated TS structures. **b,** Overlay of generated (pink) and reference (blue) structures for representative cases with low, medium, and high RMSD.

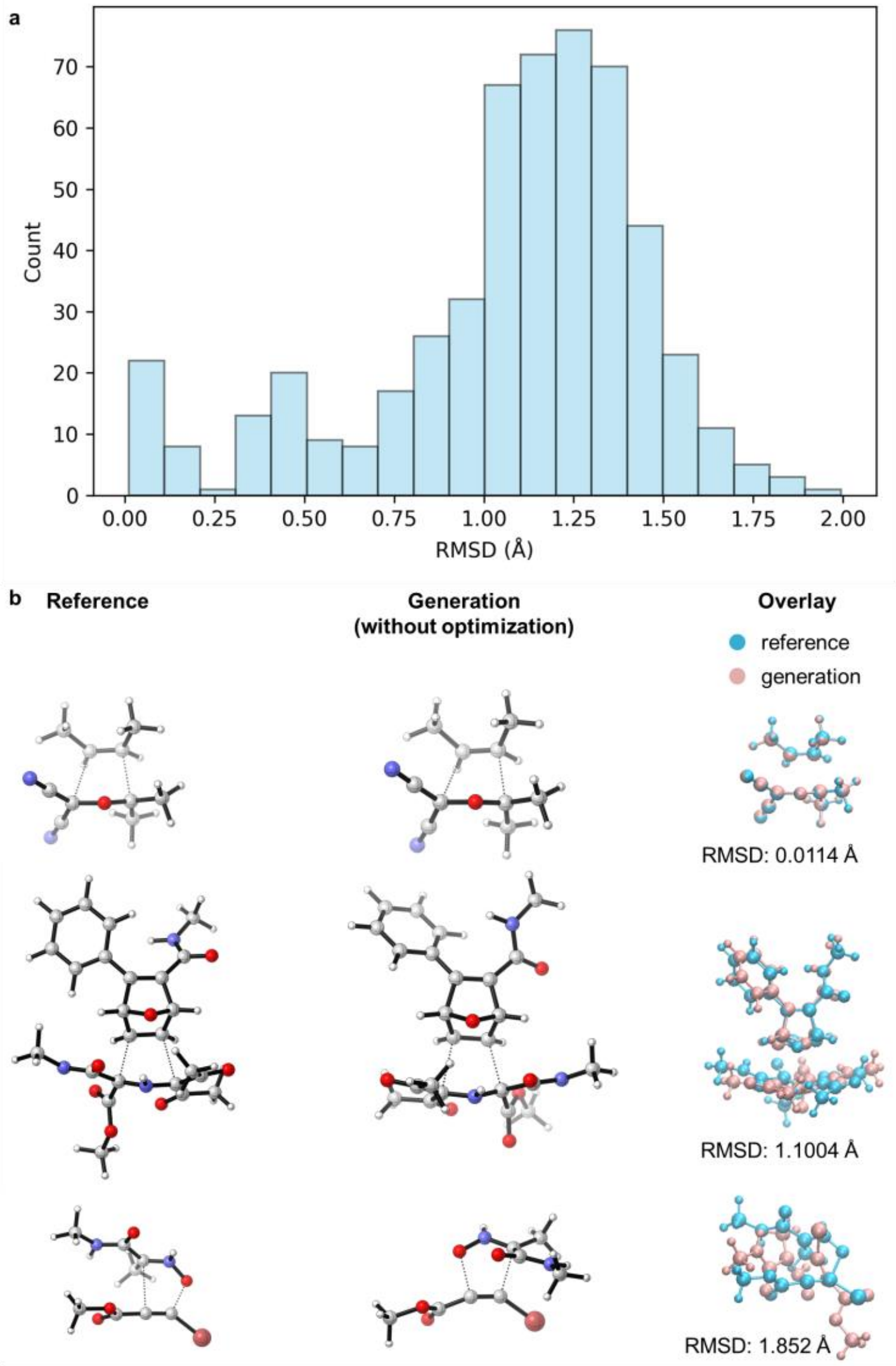

Fig. 3 RMSD distribution and structural examples from the [3+2] cycloaddition test set. a, Histogram of RMSD values for generated TS structures. **b,** Overlay of generated (pink) and reference (blue) structures for representative cases with low, medium, and high RMSD.

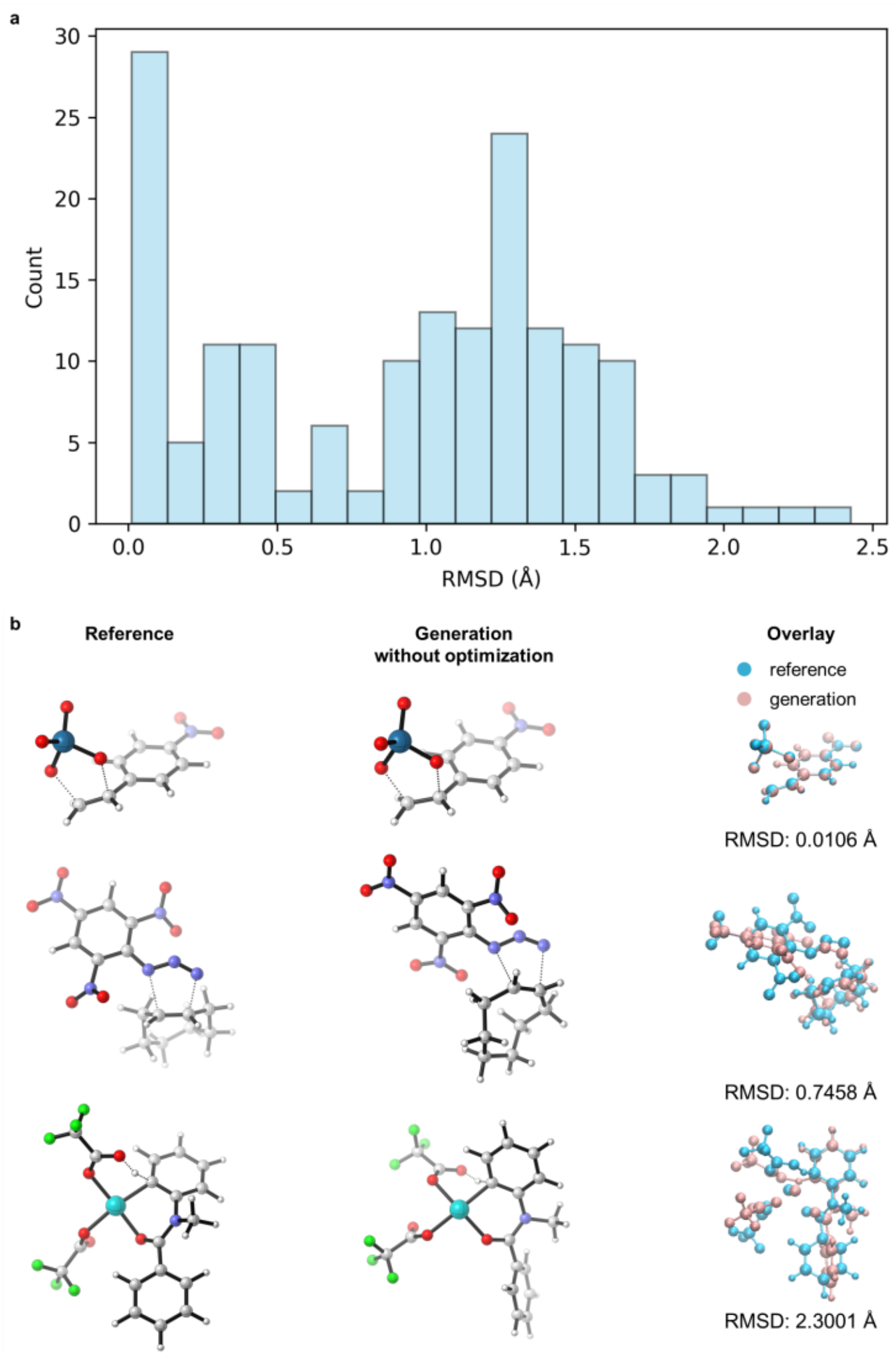

Fig. 4 RMSD distribution and structural examples from the UniTS-Lib test set. a, Histogram of RMSD values for generated TS structures. **b,** Overlay of generated (pink) and reference (blue) structures for representative cases with low, medium, and high RMSD.

For the Transition1x dataset, which contains the smallest molecules, the overall RMSD values are low: 0.1675 Å corresponds to a conformational difference, while 0.9975 Å reflects a configurational difference (Figure 2b). For the [3+2] cycloaddition dataset, an RMSD of around 1.1 Å indicates configurational differences, and the intrinsic propensity of this dataset to generate multiple configurations leads to a broad RMSD distribution around 1.25 Å (Figure 3a). For UniTS-Lib, configurational differences can result in RMSD values exceeding 2 Å (Figure 4b). Notably, all nine generated structures shown in these figures successfully converged to correct transition states upon DFT optimization.

Regarding the reviewer's suggestion to compute barrier errors, we would like to point out that the reference structures in these datasets, especially those in UniTS-Lib, were not subjected to systematic conformational searches. Therefore, comparing barrier heights relative to a single reference may not be meaningful, as a single-sample generated conformation could be either higher or lower in energy than the reference. Instead, we emphasize that by generating multiple samples (e.g., 10 per reaction or more), users can identify TS guesses that are either structurally closer to the

reference or energetically more favorable. This multi-sampling strategy is a key advantage of our generative approach. We have incorporated the above results into the revised Supplementary Information.

Comment: 9. The application section is too limited for the claims being made. Two case studies are not enough to support broad claims about selectivity analysis or mechanistic discovery. The claim that the model found a lower-barrier structure than the literature in the [3+2] example is especially overinterpreted; this could reflect many factors besides superior generative capability.

Response: We thank the reviewer for the critical and constructive assessment of our application section. Our intention was to provide proof-of-concept illustrations, not comprehensive coverage of all mechanistic scenarios. We chose these two examples because both are Formula-OOS cases, thus demonstrating the model's potential on previously unseen chemical systems, and both involve selectivity, which allowed us to showcase the model's ability to generate site-conditioned TS guesses (the C–H vs C–F activation) and its capability to sample diverse stereochemically distinct configurations (the [3+2] cycloaddition).

We agree with the reviewer that the claim about finding a lower-barrier structure in the [3+2] cycloaddition example could be overinterpreted. In the revised manuscript, we have toned down this claim and instead focus on highlighting the model's ability to sample multiple plausible TS configurations from a single 2D input.

Additionally, to further broaden the application scope, we have added a new case study on ambimodal TS, where the same transition state leads to multiple products. This represents a distinct type of mechanistic analysis that goes beyond comparing barrier heights for selectivity. We believe this addition, together with the revised language, provides a more balanced and appropriately scoped demonstration of the model's utility.

---

minor comments

Comment: The title and abstract should be toned down substantially. "Universal framework" is not supported by the results.

Response: We thank the reviewer for this suggestion. We agree that "universal framework" was too strong. We have changed the title to "unified framework" to reflect that our contribution provides an integrated pipeline combining TS data curation and generative model development. We believe our model is potentially universal in terms of architecture, as demonstrated by the Formula-OOS results. We will further expand the training set and address current limitations to move closer to a truly "universal" framework. A discussion of these future directions has been added to the revised manuscript.

Comment: The manuscript should clearly distinguish "first-order saddle point" from "correct transition state for the intended pathway." At present these ideas are blurred.

Response: We thank the reviewer for this suggestion. In the revised manuscript, we have gone through all relevant sections and clearly distinguished among three levels of validation: (1) "first-order saddle point" (a stationary point with one imaginary frequency); (2) "reactive transition

state" (first-order saddle point further validated by graph-matching algorithms to confirm bond-forming/-breaking events rather than simple conformational changes); and (3) "correct transition state for the intended pathway" (additionally confirmed by IRC to connect the expected minima). We believe this addresses the concern.

Reviewer #1 (Remarks on code availability):

Comment: I do not have enough time to dig into the codes this time and I think there are major issues in the work that need to be addressed first prior to that.

Response: We thank the reviewer for the time and thorough, critical assessment throughout this process. We have carefully considered all of the reviewer's comments and have made extensive, detailed revisions to address the issues raised. We believe the manuscript has been substantially improved as a result.

Response to Referee #2

Comment: In this manuscript, Xu and co-workers present a general framework for automated transition-state generation that combines a curated dataset (UniTS-Lib) with a diffusion-based generative model (UniTS-Gen). The approach enables the direct generation of three-dimensional TS structures from two-dimensional reactant representations and addresses a longstanding challenge in computational chemistry: the efficient construction of reliable TS initial guesses for mechanistic exploration. The authors compile a diverse dataset of 4,391 high-quality TS structures spanning a broad range of elements and reaction types, and develop a higher-degree equivariant diffusion model to generate TS geometries. The benchmarking results demonstrate strong accuracy and promising generalization capability, including transferability to previously unseen reaction classes. The framework is further shown to accelerate TS discovery by providing high-quality initial structures for subsequent optimization.

Overall, this work addresses an important and timely problem at the interface of computational chemistry and machine learning. The integration of dataset construction, methodological innovation, and application to mechanistic studies represents a valuable contribution. The manuscript is clearly written and technically sound. I recommend publication in Nature Communications after minor revision:

Response: We thank the reviewer for the positive and constructive evaluation. We have revised the manuscript following the suggestions.

Comment: 1) While prior TS generation methods are discussed, a more explicit comparison with representative state-of-the-art approaches (in terms of accuracy, robustness, or computational efficiency) would help better contextualize the advantages of the present method.

Response: We thank the reviewer for this constructive suggestion. Following the reviewer's advice, we compared our model with representative state-of-the-art methods (TSDiff, OA-ReactDiff, and React-OT) on the Transition 1x dataset. The results are shown in Table 1.

Table 1 Comparison of different models in terms of input type, accuracy, and computational efficiency.

Method	Input type	Mean of RMSD (Å)	Inference per reaction (s)
TSDiff	2D graph	0.2526	-
OA-ReactDiff (1 times)	3D coordinate	0.1800	6.80
OA-ReactDiff (40 times)	3D coordinate	0.1297	272.00
React-OT	3D coordinate	0.1029	0.39
React-OT + pretraining	3D coordinate	0.0981	0.39
UniTS-Gen (HiEGNN, 1 times)	2D graph	0.2517	0.45
UniTS-Gen (HiEGNN, 10 times)	2D graph	0.0905	4.5

In terms of single-sample RMSD, models that rely on 3D reactant and product structures as input (React-OT and OA-ReactDiff) achieve the lowest values, mainly because the provided 3D

geometries offer a strong initial prior. However, this also implies that such models are more sensitive to the quality of the reference input, which to some extent limits their applicability in more exploratory tasks.

It is worth noting that the relatively higher single-sample RMSD of UniTS-Gen does not arise from obvious structural errors (as indicated by the clash ratio in Fig. 4b of the main text), but rather from stereochemical differences between the generated TS and the reference structure. This issue can be effectively alleviated by multi-sampling: with 10 samples, the mean RMSD of UniTS-Gen becomes the lowest among all compared methods.

Another model that takes 2D molecular graphs as input is TSDiff, whose denoising network is built on the invariant GNN (SchNet). While this model works reasonably well on relatively simple systems, it may struggle on complex and sparse datasets such as UniTS-Lib, just like EGNN.

Regarding inference time, we have included the average time per reaction in Table 1. However, we would point out that a direct comparison may not be fully meaningful because we do not know the exact GPU specifications or batch size settings used for the other methods. Our own inference times were measured on a single NVIDIA H800 GPU with a batch size of 128, averaged over the entire Transition 1x test set. For the most complex and practically relevant scenario, the UniTS-Lib Formula-OOS dataset, the average inference time per single sample is approximately 10.22 seconds.

We would also like to add a practical note: thanks to GPU parallelism, in typical mechanistic studies where only a small number of transition states need to be generated at once, the difference between 1 and 10 samples is negligible. For instance, both 1 and 10 samples for a single reaction can be finished in one inference batch with a batch size of 16. We have included these results in the revised Supplementary Information.

Comment: 2) It would be interesting to explore whether the proposed TS generation approach can be extended to more complex scenarios, such as ambimodal transition states (e.g., Ess, Wheeler, Houk, and co-workers, Angew. Chem. Int. Ed. 2008, 47, 7592–7601). Even a brief discussion of the potential applicability or limitations in this context would further highlight the scope of the method.).

Response: We are particularly grateful to the reviewer for this constructive suggestion, which has helped us further broaden the application perspective of our model.

To respond to the reviewer's suggestion, we selected a representative ambimodal transition state from a recent 2026 study (*J. Am. Chem. Soc.* **148**, 8723–8735 (2026)), in which the transition state bifurcates to give both [4+1] and [2+1] cycloaddition products in molecular dynamics simulations. Notably, the combination of elements in this transition state has never appeared in UniTS-Lib, providing a valuable Formula-OOS test case for evaluating the generalization capability of our model on truly unseen systems.

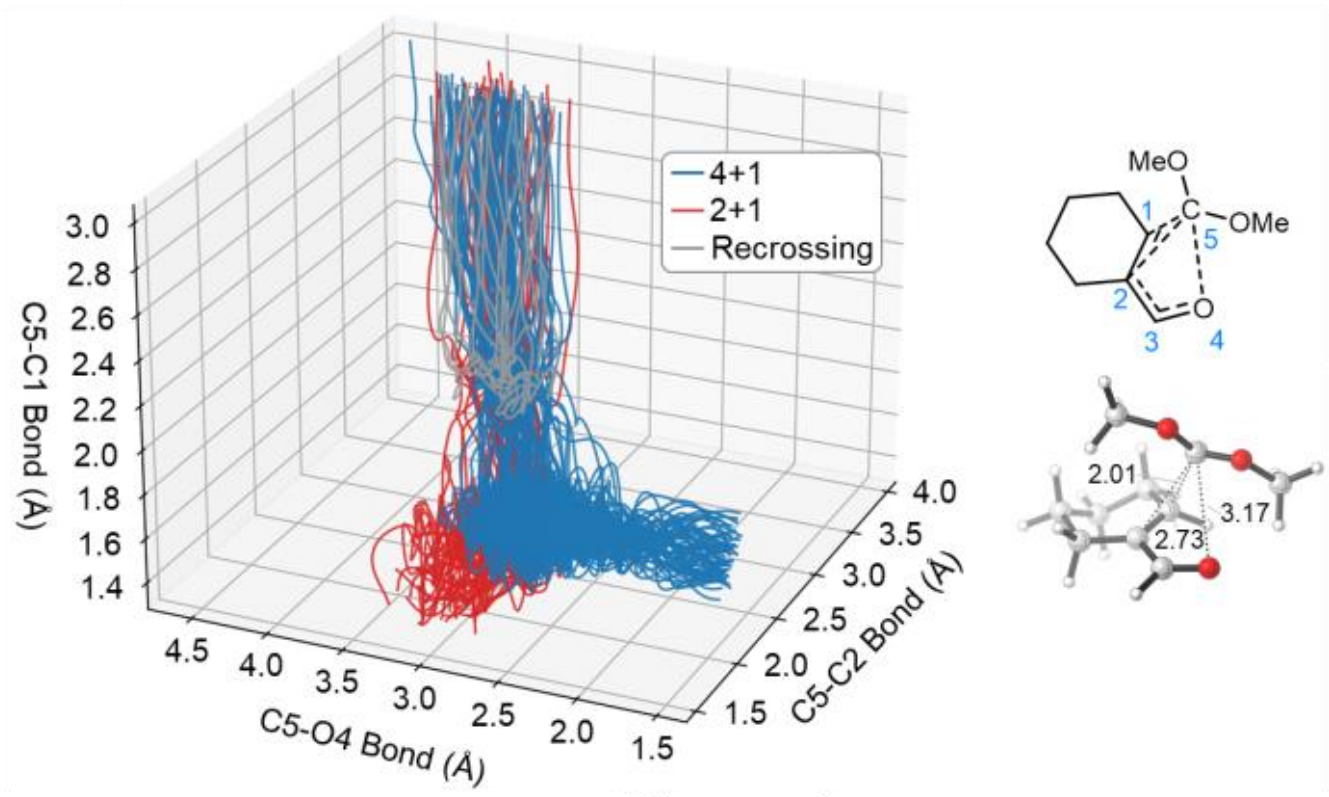

Fig. 5 Overlaid 3D trajectories from quasi-classical molecular dynamics simulations of the ambimodal transition state.

We used the molecular graph together with the C1–C5 atom pair index (this bond inevitably form/break during the reaction) as input and performed 10 sampling runs. Among the generated structures, we selected a relatively stable conformation for TS optimization, which successfully converged to the correct transition state. We then performed quasi-classical molecular dynamics simulations based on this TS using the same parameter settings as reported in the original study. Due to computational limits, we were able to complete 113 full trajectories.

Encouragingly, we observed two distinct products from these trajectories, with a cycloaddition product ratio of [4+1]:[2+1] = 63:41, along with 9 recrossing events (see Figure 5). This ratio differs somewhat from the original report, which may be due to the slightly different TS conformation used and the much smaller number of trajectories we ran. Nevertheless, the successful observation of bifurcation demonstrates that our model can be applied to ambimodal transition state scenarios that are unknown, complex, and highly sensitive to bond lengths in the reactive region.

We thank the reviewer again for this valuable suggestion, which has meaningfully expanded the scope of our model's applicability in mechanistic exploration. The corresponding results and discussion have been added to the application section of the revised manuscript.

Comment: 3) A brief clarification of technical terminology (e.g., “higher-degree equivariant network”) would improve accessibility for a broader readership.

Response: We thank the reviewer for this helpful suggestion. To improve accessibility for a broader readership, we have added detailed explanations of key technical terms, including “higher-degree SE(3)-equivariant graph neural network”, “diffusion model”, and “topology-augmented scalar features” in the Methods section of the revised manuscript. We believe these additions will make the manuscript more accessible to readers.

Response to Referee #3

Comment: This is valuable work on the field of generative AI for transition states, remarkably supporting metals and a wide diversity of complex reactions. The TS library will be a useful resource for the community. The topology-informed 2D graph to 3D geometry transform is appealing and appears to be a superior alternative to previous diffusion models. Adding further value, the study goes beyond conventional metrics and tests the generated TS geometry guesses via actual DFT saddle point optimizations, yielding a success rate similar to what an experienced computational chemist would achieve.

Response: We sincerely thank the reviewer for the positive and encouraging assessment of our work, and for recognizing the value of the data resource, the strengths of our 2D-informed diffusion design, and the practical relevance of our DFT validation results.

Comment: Minor revisions:

Fig. 2a: The chords color legend can be better - Nearly only two colors appear to be relevant in the plot.

Response: We thank the reviewer for this careful and constructive suggestion. Accordingly, we have adjusted the mapping between element co-occurrence frequency and chord thickness in Fig. 2a, ensuring that pairs with lower frequencies (below 100) remain clearly visible (as shown in Figure 6). In addition, to further clarify the distribution of co-occurrence frequencies among different elements in UniTS-Lib, we have included four separate chord diagrams (see Figure 7) in the Supplementary Information, covering the frequency ranges of 1–10, 10–10^2, 10^2–10^3, and 10^3–10^4. We believe these revisions improve the clarity of the element co-occurrence visualization.

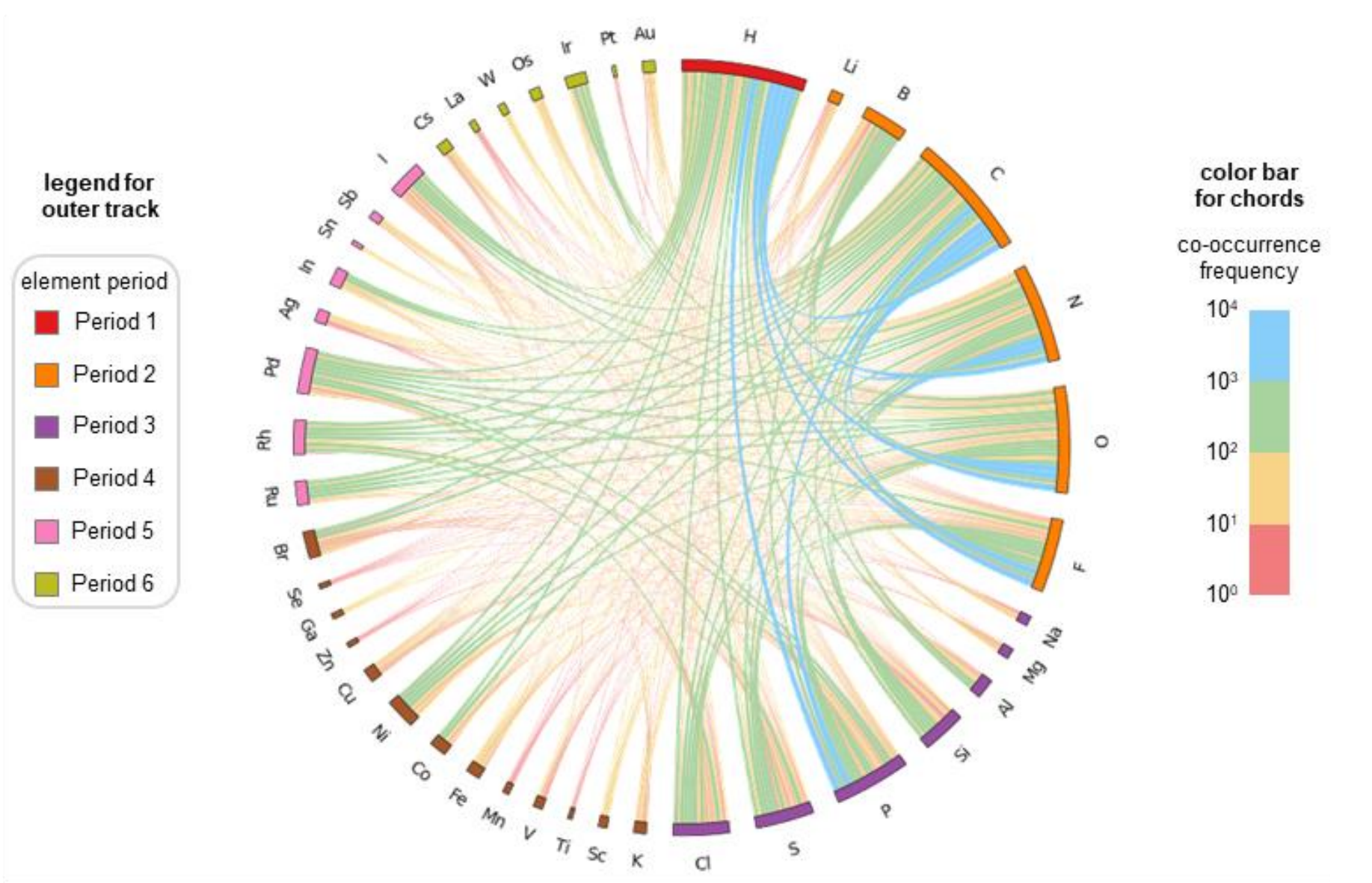

Fig. 6 Revised element co-occurrence chord diagram illustrates the elemental richness and relationships within UniTS-Lib.

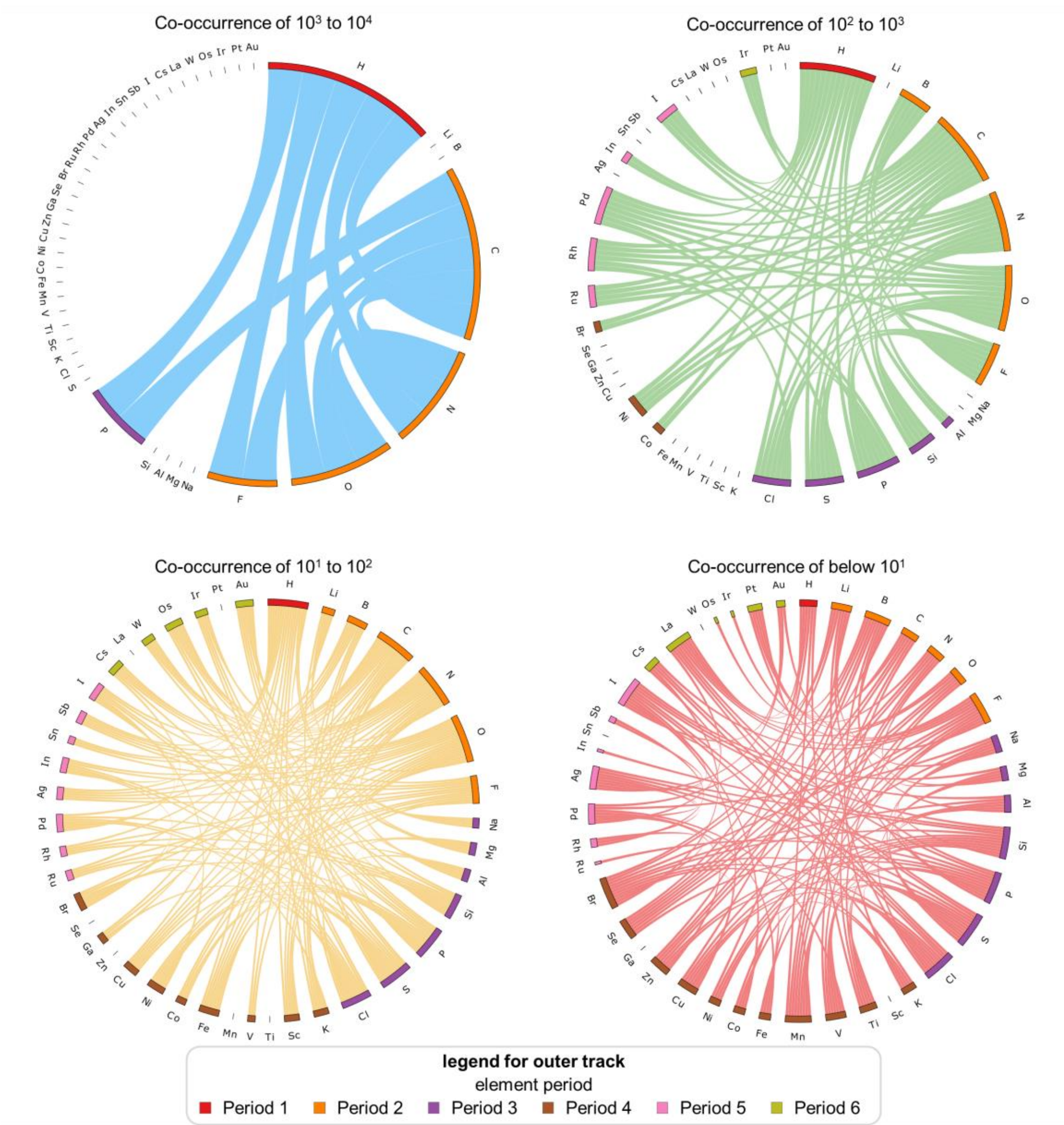

Fig. 7 Element co-occurrence chord diagrams for UniTS-Lib across four frequency ranges.

Comment: Fig. 2 analysis: If possible, give statistics over reaction types.

Response: We thank the reviewer for this kind suggestion. We agree that providing reaction type information helps readers understand the composition of the dataset. In fact, in the previous version of the Supplementary Information (Supplementary Figure 2), we already provided a list of 12 representative reaction types (e.g., oxidative addition, transmetalation, C–H activation, cycloaddition, etc.) to illustrate the breadth of transformations covered by UniTS-Lib.

We would like to clarify that a quantitative analysis by reaction type is not provided because many transition states in UniTS-Lib originate from complex mechanistic studies. As a result, a single TS structure may not be uniquely assignable to a single reaction type. Under such circumstances, providing seemingly precise statistical numbers could be misleading. We therefore chose to present the reaction type diversity qualitatively, complemented by other metrics such as elemental diversity

and atom count distribution in Fig. 2, to collectively convey the broad coverage of UniTS-Lib. We have clarified this consideration in the revised manuscript.

Comment: Fig 3: "defuse" should be "diffuse"?

Response: We thank the reviewer for catching this typo. The figure has been corrected from "defuse" to "diffuse". We have also rechecked the entire manuscript for similar errors.

Comment: "multi-configurational" may cause confusion (multi-reference in electronic structure theory) - try to find an alternative term.

Response: We thank the reviewer for this clarification. To avoid ambiguity, we have replaced "multi-configurational" with "stereochemically diverse" throughout the manuscript.

Comment: The Methods section is too succinct. Give more detail. In the Computational Details, state (if true) that no IRC calculations were carried out. In the UniTS-Gen Architecture, give some minimal math expressing the architecture of the model (of particular interest is the 2D-conditioning of the 3D geometry generation) and the loss function used to train it. TASFs: In the equation, what is the difference between the $embeddingi0$ terms on both sides of the equals sign? Revise the text discussing the TASFs making a bit longer if possible and also clearer.

Response: We thank the reviewer for these helpful suggestions. Accordingly, we have expanded the Computational Details in SI and Methods section in main text as follows:

1. In computational details of SI, we now explicitly state that no IRC calculations were performed during the initial construction of UniTS-Lib (only frequency analysis and graph matching were used), while for the 169 generated TS structures in the Formula-OOS set that passed filtering, IRC validation has been added.

2. In the UniTS-Gen architecture subsection of Methods section, we have added detailed descriptions and key mathematical expressions describing the higher-degree SE(3)-equivariant graph neural network, the diffusion process, the loss function used for training, and how the 2D conditioning guides the 3D equivariant generation.

3. Regarding the TASFs equation, we have updated equals:

$$h_i^{0,new} = h_i^{0,old} + Bridge\left(x_i^{2D}\right)$$

where $h_i^{0,old}$ (here we have updated the notation from $embedding$ to h) denotes the degree-0 embedding of node i before topological injection, $h_i^{0,new}$ denotes the updated degree-0 embedding after topological injection, and x_i^{2D} is the corresponding 2D-derived node feature. The surrounding discussion of TASFs has been expanded accordingly.

We believe these additions have substantially improved the completeness and readability of the Methods section.

Comment: Congratulations for the impressive work done.

Response: We sincerely thank the reviewer for positive and encouraging assessment of our work.

Response to Referee #1

Comment: The authors have done a great job in this revision. They tone down many statements, making this high-quality manuscript more rigorous. I also like the new results on quasi-classical dynamics and overlapping visualization examples for different dataset, which makes it much clear. My only remaining comment is about energy barriers for different datasets. I understand the authors' arguement regarding to conformations, but the same arguement holds for RMSD, no? I would encourage the authors to compute energy barriers in some ways, so that the audience can have a idea about how barrier errors changes among different datasets and molecule sizes.

Response: We sincerely thank the reviewer for the positive assessment of our revised manuscript and for all the constructive comments throughout the review process, which have substantially improved the quality of our work.

We also appreciate the reviewer's further suggestion regarding cross-dataset analysis of barrier errors. We agree that providing concrete barrier error data for the three datasets will help readers better understand how structural deviations translate into energy differences across different systems. To this end, using the three representative structures (low, medium, and high RMSD) selected from each dataset in the previous revision, we performed the following analyses: (1) we compared the single-point energy differences between the generated structures and the reference geometries without optimization; (2) we then uniformly optimized all structures (including the reference geometries, because the original references were not computed with a consistent method), performed IRC calculations, optimized the end-points along the IRC paths, and calculated the corresponding free energy barriers. Finally, we compared the barrier differences between the generated and reference structures. All calculations were carried out using the same level of theory (B3LYP-D3BJ/def2SVP). The results are presented in Figures 1–3.

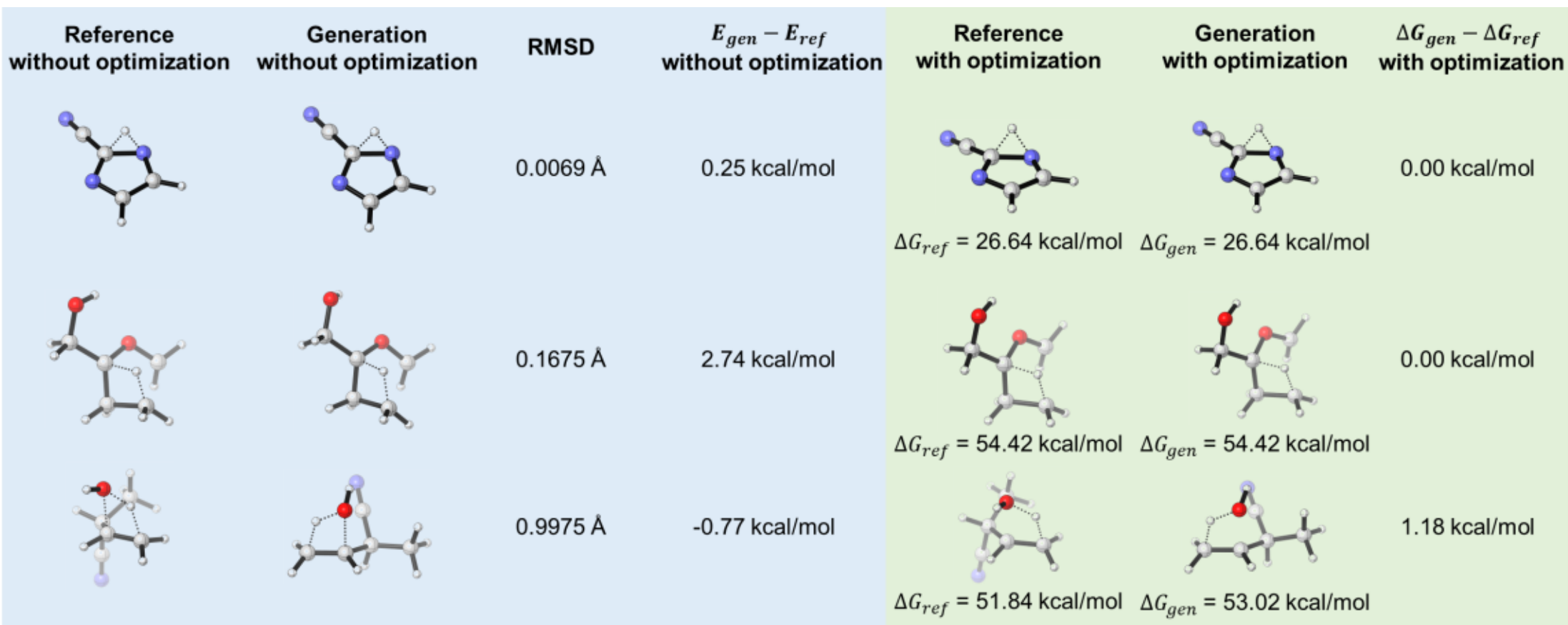

Fig. 1 RMSD and barrier error of representative structures from Transition1x.

As shown in Figures 1–3, for all three datasets, when the RMSD between the generated and reference structures is around 0.01 Å or lower, the single-point energy difference is small even without optimization (first row of each figure). Among these, UniTS-Lib, which contains the most complex molecules, shows the largest single-point energy difference within this group, yet it remains below 1 kcal/mol (Figure 3, first row). When RMSD reaches medium or high levels, the single-point energy differences become substantial. For the Transition1x dataset, the energy

differences are relatively small due to the simpler molecular systems; the largest difference (2.74 kcal/mol) is observed for the medium-RMSD case, which corresponds to a conformational difference. After DFT optimization, the free energy barrier difference for the low-RMSD and the medium-RMSD (conformational difference) cases became negligible (0.00 kcal/mol, Figure 1, first and second rows). However, for the high-RMSD case (configurational difference), a free energy barrier difference of 1.18 kcal/mol remained after optimization (Figure 1, third row).

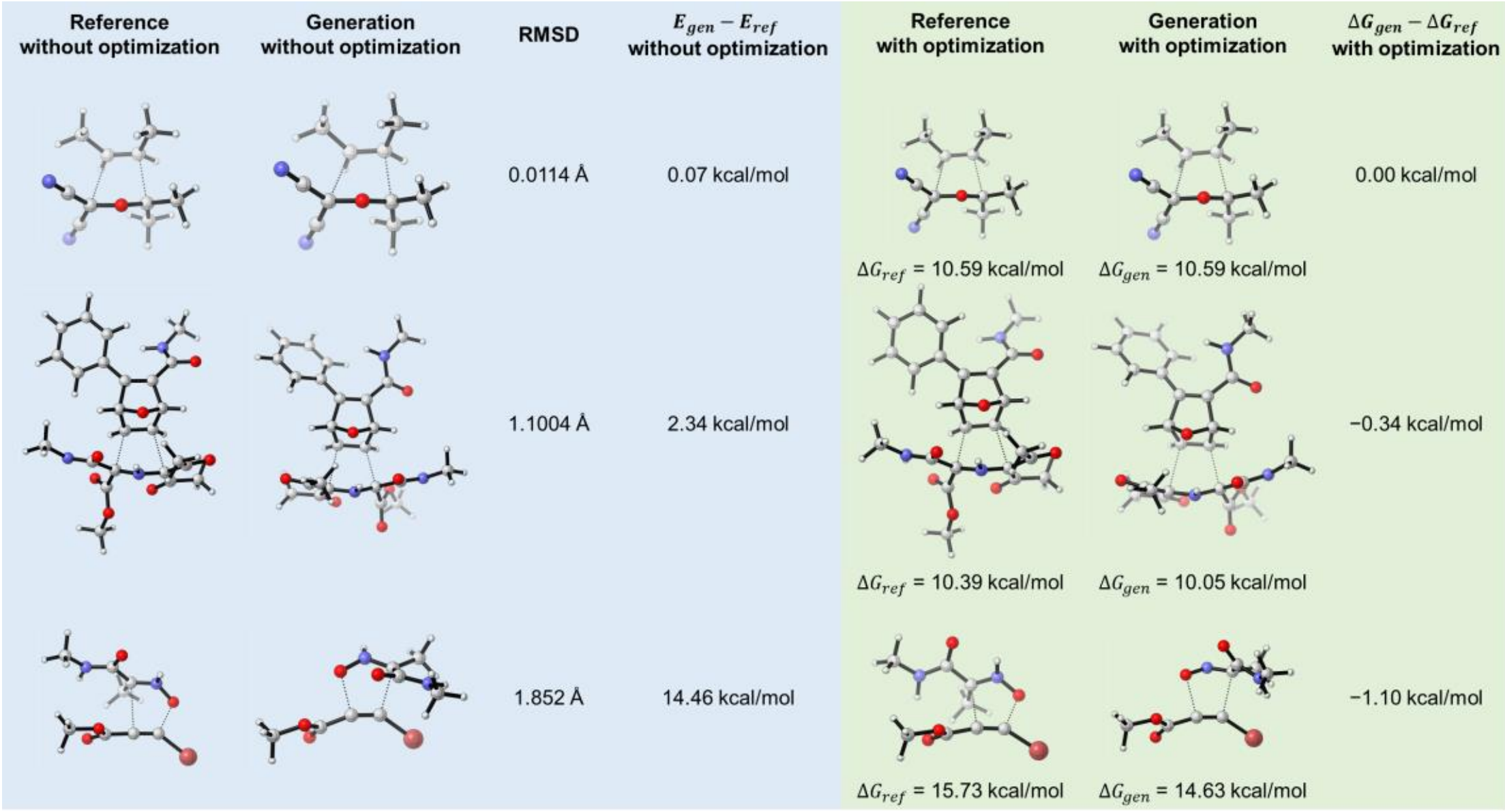

Fig. 2 RMSD and barrier error of representative structures from [3+2] cycloaddition dataset.

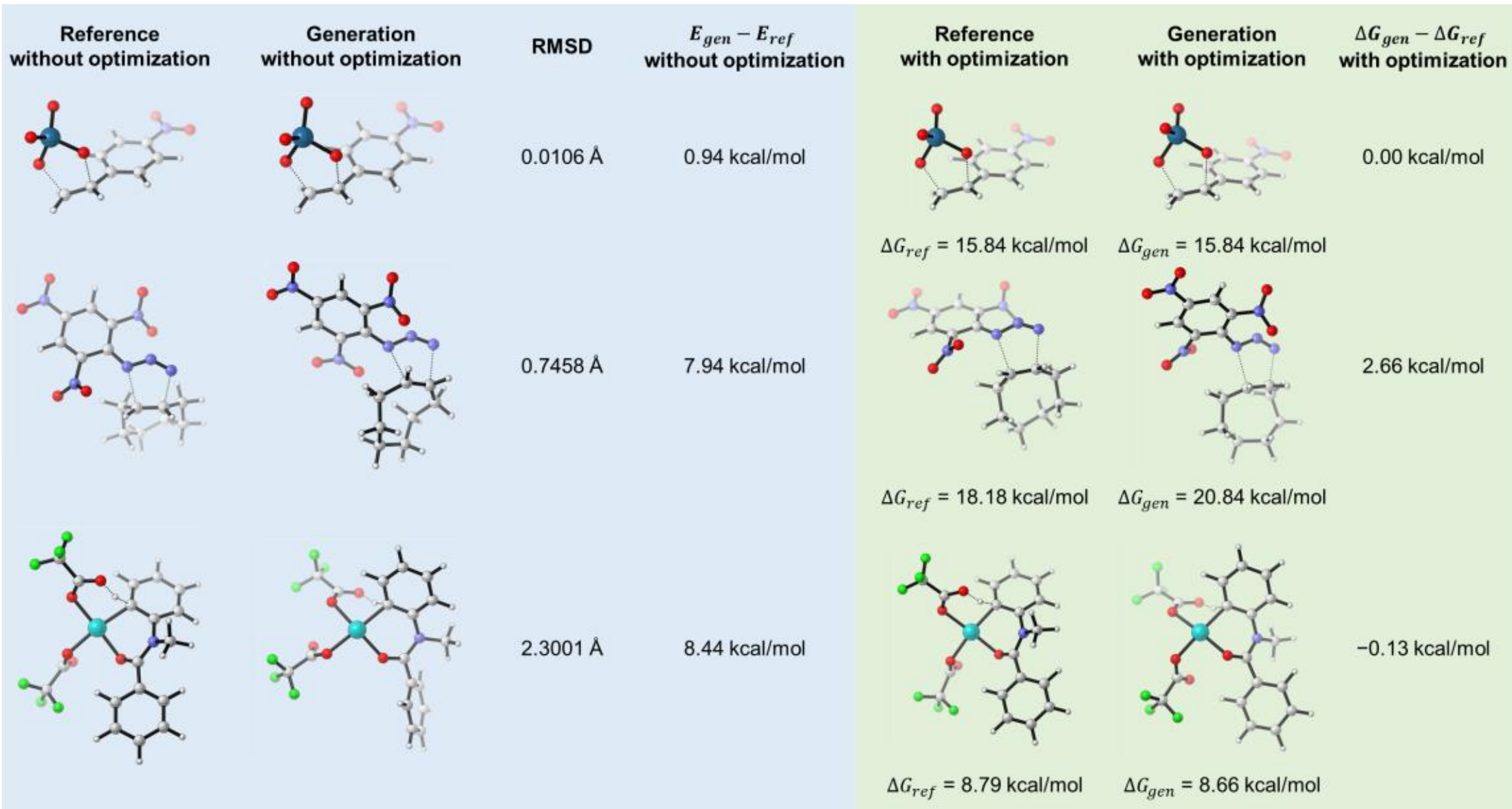

Fig. 3 RMSD and barrier error of representative structures from UniTS-Lib.

For the more complex [3+2] cycloaddition and UniTS-Lib datasets, the medium- and high-RMSD structures exhibit considerably larger single-point energy differences relative to the references (Figures 2 and 3, second and third rows). This is mainly because, in these complex systems, small changes in bond lengths and angles related to bond-forming/-breaking events can lead to significant energy variations. Nevertheless, after DFT optimization, the barrier differences between the

generated and reference structures are substantially reduced.

These cross-dataset analyses linking structural deviations to energy errors provide readers with a clearer and more quantitative understanding. These results have been added into section 12 of the revised Supplementary Information.

Response to Referee #2

Comment: The authors have thoroughly and convincingly addressed all of this reviewer's concerns. In particular, they demonstrate that their model can successfully handle ambimodal transition-state scenarios, which are inherently complex, previously unexplored, and highly sensitive to subtle bond-length variations within the reactive region. This represents a particularly compelling validation of the robustness and generality of the approach.

Importantly, the study highlights the potential of the model to address challenging mechanistic problems that are difficult to analyze using conventional intuition alone, making the work highly relevant to both synthetic and mechanistic chemists. Overall, this work demonstrates substantial conceptual and methodological significance, and I strongly support its publication in Nature Communications

Response: We sincerely thank the reviewer for the very positive and encouraging assessment of our work. We are especially grateful for the reviewer's suggestion in the previous revision to apply our model to complex ambimodal transition state scenarios, which significantly enriched the depth and scope of this study.

Response to Referee #1

Comment: I thank the authors for adding the barrier error examples, which give us an estimation about the relationship between RMSD and barrier errors. I recommend its publication now.

Response: We sincerely thank the reviewer for the positive evaluation of our work and for recommending our manuscript for publication. We greatly appreciate the reviewer's time, constructive comments, and support throughout the review process.
